# Functional Verification of Two Genes Related to Stripe Rust Resistance in the Wheat-*Leymus mollis* Introgression Line M8664-3

**DOI:** 10.3389/fpls.2021.754823

**Published:** 2021-10-25

**Authors:** Pengfei Jin, Kaixiang Chao, Juan Li, Zihao Wang, Peng Cheng, Qiang Li, Baotong Wang

**Affiliations:** ^1^State Key Laboratory of Crop Stress Biology for Arid Areas, College of Plant Protection, Northwest A&F University, Yangling, China; ^2^College of Chemistry, Biology and Environment, Yuxi Normal University, Yuxi, China; ^3^Dingxi Plant Protection and Quarantine Station, Dingxi, China

**Keywords:** functional verification, stripe rust, resistance, wheat-*Leymus mollis*, *YrM8664-3*

## Abstract

Stripe rust, caused by *Puccinia striiformis* f. sp. *tritici* (*Pst*), is one of the most widespread and destructive fungal diseases of wheat worldwide. The cultivation and growth of resistant wheat varieties are the most economical, effective, and environmental friendly methods to control stripe rust. Therefore, it is necessary to use new resistance genes to breed resistant wheat varieties. A single dominant gene temporarily designated as *YrM8664-3*, from a wheat-*Leymus mollis* introgression line M8664-3 highly resistant to Chinese predominant *Pst* races, is a potentially valuable source of stripe rust resistance for breeding. Herein, based on previous *YrM8664-3* chromosome location results (bin 4AL13-0.59-0.66 close to 4AL12-0.43-0.59) and expression change information of candidate genes and bioinformatics analysis, several candidate genes with significantly different expression changes were then selected and verified by virus-induced gene silencing (VIGS). Two of the candidate genes temporarily designated as *TaFBN* [containing plastid lipid-associated proteins (PAP)_fibrillin domain in its protein] and *Ta_Pes_BRCT* [containing Pescadillo and breast cancer tumour suppressor protein C-terminus (BRCT) domain in its protein], produced the most significant resistance changes in the wheat-*Pst* interaction system after silencing. These two genes were further verified by *Agrobacterium*-mediated wheat genetic transformation technology. According to the identification of disease resistance, the resistance function of the candidate gene *TaFBN* was further verified. Then, the expression of *TaFBN* under hormone treatment indicated that *TaFBN* may be related to the salicylic acid (SA) and abscisic acid (ABA) signaling pathways. Combined with the expression of *TaFBN* in response to environmental stress stimulation, it can be reasonably speculated that *TaFBN* plays an important role in the resistance of wheat to *Pst* and is involved in abiotic stress pathways.

## Introduction

Wheat (*Triticum aestivum* L.) is one of the most important food crops in the world. Wheat stripe rust, caused by *Pst*, is one of the most important diseases that affects wheat production. It has the characteristics of rapid outbreaks that cause regional epidemics and subsequent extensive and severe harm to wheat crops, and it has been found in almost all major wheat-producing areas in the world. When wheat is susceptible to *Pst* infection, the yield loss is ~10–20%, but it can exceed 50% or even result in no harvest in severe cases (Wan et al., 2007). Since the 1950s, several nationwide stripe rust epidemics have been recorded in China, among which, a yield loss of 6, 3.2, 2.65, and 1.3 billion kg of wheat occurred in 1950, 1964, 1990, and 2002, respectively (Wan et al., 2004). In 2017, wheat stripe rust was once again epidemic throughout China, affecting an area of 5.56 million hm^2^, which was the largest annual occurrence since 2002 (Huang et al., 2018). The most effective, economical, and environmental friendly method to control stripe rust is to breed and grow disease-resistant wheat varieties.

Disease resistance in wheat varieties, like all plants, often depends on pattern-triggered immunity (PTI) brought about by microbial patterns *via* pattern-recognition receptors (PRRs) localized on cell surfaces, and effector-triggered immunity (ETI) activated by pathogen effector proteins *via* predominantly intracellular localized receptors called nucleotide-binding, leucine-rich repeat receptors (NLRs) (Jones and Dangl, 2006; Cui et al., 2015; Yu et al., 2017; Yuan et al., 2021). The disease resistance reaction produced by plants is a complex and orderly process, and is also regulated by multiple genes, especially resistance (R) genes (Jia et al., 2000; Dodds et al., 2006; Luo et al., 2011).

By analyzing protein sequences encoded by cloned R genes, it was found that R genes targeted at different sources and pathogens possessed similar characteristic domains, such as nucleotide binding site (NBS), leucine-rich repeat (LRR), toll-interleukin-1 receptor (TIR), coiled-coil (CC), protein kinase (PK), and the transmembrane domain (TM) (Jones et al., 2016; Monteiro and Nishimura, 2018; Ma et al., 2020). In addition, plant disease resistance is closely related to hormone signal transduction and environmental stress stimulation (Denance et al., 2013; Derksen et al., 2013). At present, salicylic acid (SA), jasmonic acid (JA), ethylene (ET), abscisic acid (ABA), and other signaling molecules involved in plant disease resistance have been extensively studied (Schenk et al., 2000; Denance et al., 2013; Derksen et al., 2013). Therefore, studies on plant disease resistance genes will assist in expanding our understanding of the mechanism of resistance at a deeper level, and will provide some reference for disease control.

Histopathological studies on the interaction between wheat and *Pst* are the basis for revealing the detailed process of *Pst* infection and host resistance. Kang et al. (2002) found that the growth of a pathogenic fungus was inhibited in resistant varieties compared with that in susceptible varieties. Reactive oxygen species (ROS) bursting is one of the fastest and most effective disease resistance reactions in the interaction between plants and their pathogens. By dyeing leaf tissues with diaminobenzidine (DAB), Wang et al. (2007) found that ROS were produced in guard cells of both compatible and incompatible combinations after the interaction. In terms of biochemistry, the resistance of wheat varieties to *Pst* is mainly reflected in protective enzymes, such as superoxide dismutase (SOD) and phenylalanine ammonia-lyase (PAL), as well as the increase in activities of defensive enzymes, such as antimicrobial hydrolase and chitinase, and the increase in resistant proteins and lignin in host cell walls (Asthir et al., 2011; Zheng et al., 2020).

Virus-induced gene silencing is an RNA interference-based technology that transiently knocks down a target gene expression using modified plant viral genomes. When a targeted gene is inserted into a viral genome and a plant is inoculated with viruses, plant cells recognize the threat of the invading viruses and use protective defense mechanisms to destroy any foreign genes carried by viruses and viral vectors. Loss of function phenotype or decreased expression activity of the target gene occurs, and then the function of the target gene can be identified according to phenotypic changes (Scofield et al., 2005; Feng et al., 2015).

The identification of wheat disease-resistance genes and studies on disease-resistance mechanisms are the basis of wheat disease-resistance breeding and disease control. M8664-3 is the hybrid offspring of common wheat cultivar 7182 and wheat-related species *Leymus mollis* (Trin) Hara. Our previous studies have shown that the dominant gene *YrM8664-3* in M8664-3 confers all-stage resistance to Chinese prevalent *Pst* race CYR33. *YrM8664-3* was located in bin 4AL13-0.59-0.66 close to 4AL12-0.43-0.59 on chromosome 4AL and flanked by single-nucleotide polymorphism markers *AX111655681* and *AX109496237* with genetic distances of 5.3 and 2.3 centimorgans, respectively (Chao et al., 2018). However, because of the alien chromosome fragment that may exist in the *YrM8664-3* region, it is difficult to further finely map and clone the gene. The sequencing of the entire genome of wheat variety Chinese Spring has been completed, making it possible to identify candidate genes and perform functional verification analysis of *YrM8664-3* in the reference genome region corresponding to the located chromosome interval.

To identify the candidate genes involved in stripe rust resistance in M8664-3, in this study, the resistance of M8664-3 to CYR33 was investigated and then analyzed. Combined with histological and histochemical techniques, the invasion and infection processes of CYR33 on M8664-3 were studied in detail, and the resistance process of M8664-3 to CYR33 was comprehensively analyzed. Based on the physical location of *YrM8664-3*, the genes related to disease resistance were selected from the annotated database of the Chinese Spring genome according to the structure domains related to disease resistance and function prediction information. Then, a functional validation analysis was carried out to identify the disease-resistant genes. The finding in this study could provide a basis for wheat stripe rust resistance breeding.

## Materials and Methods

### Plants and Pathogens

The wheat-*Leymus mollis* introgression line M8664-3 used in this study was provided by Professor Jie Fu, College of Agronomy, Northwest A&F University, Yangling, China.

Chinese predominate *Pst* race CYR33 was used for seedling tests. After the identification on differential hosts of Chinese *Pst*, CYR33 was increased in susceptible variety Mingxian169.

### Seedling Tests, RNA Extraction

Seedling tests were conducted under controlled greenhouse conditions according to Wan et al. (2007) and Bansal et al. (2017). M8664-3 and Mingxian 169 were planted in 7 7 7 cm pots with 15–20 seeds per pots. When the first leaves fully expanded, fresh CYR33 urediniospores were inoculated onto wheat leaves by the smear method, and sterile water was used as MOCK-inoculation control (Roelfs et al., 1992). Approximately 14–16 days post inoculation (dpi), when obvious uredinia were observed on the leaves of Mingxian169, the types of infection types were recorded according to a 0–9 scale. Also, leaf samples were collected at 0, 12, 24, 48, 72, and 96 h post inoculation (hpi). RNA extraction was performed using a Magen plant total RNA extraction kit (Magen Biotech, Guangzhou, China). The concentration and purity of each RNA sample were evaluated using a micro-ultraviolet spectrophotometer (NanoDrop 2000; Thermo Fisher Scientific, Wilmington, DE, United States), and the integrity was determined by 1% agarose gel electrophoresis. The first strand of DNA was synthesized with HiScript II Q-RT SuperMix for qPCR (+ gDNA wiper) (Vazyme Biotech, Nanjing, China).

### Candidate Gene Selection and Quantitative Real-Time PCR (Qrt-PCR) Analysis

The sequence of the linked markers of *YrM8664-3* was blasted against the genome sequence of Chinese Spring IWGSC RefSeq v1.0 Genome (IWGSC et al., 2018) (https://wheat-urgi.versailles.inra.fr/Seq-Repository/Assemblies), and the gene was located in the range of 41.6 × 10^7^-63.9 × 10^7^ bp (base pair) on wheat chromosome 4AL (Chao et al., 2018). Referring to the IWGSC RefSeq v1.0 annotation (https://urgi.versailles.inra.fr/download/iwgsc/IWGSC_RefSeq_Annotations/v1.0/), genes near the linked markers containing conserved domains of disease resistance, such as NBS, LRR, and PK, or genes hit by the linked markers were selected.

To measure the transcriptional expression level of selected genes by qRT-PCR, specific primers (Supplementary Table 1) were designed using the Primer Premier 5.0 software (Li et al., 2011). Using *TaEF-1*α (GenBank accession number Q03033) as an internal reference gene, the relative transcription expression level of target genes was determined. All the qRT-PCR reactions were performed in a 20-μl reaction mixture containing 10 μl Cham Q^TM^ SYBR® qPCR Master Mix (Vazyme Biotech, Nanjing, China), 0.2 μl each of the forward and reverse gene-specific primers (10 μM), and 2 μl of diluted cDNA (1:10). A Bio-Rad iQ5 Real Time PCR (Bio-Rad, Hercules, CA, United States) system was used to generate cycle threshold (CT) values for the quantification of relative gene expression using the comparative 2^−ΔΔCt^ method (Livak and Schmittgen, 2001). All the samples were analyzed in three biological replications, and all the PCR analyses were replicated three times.

### Sequence Amplification, Identification, and Bioinformatics Analysis

According to the primers (Supplementary Table 2) at the positive and negative ends of the open reading frame (ORF) region, the cDNA of M8664-3 was used as the template for amplification with gene-specific primers as follows: pre-denaturation at 94°C for 2 min, followed by 35 cycles of 94°C for 30 s, 60°C for 30 s, and 72°C for 1 min, with a final incubation at 72°C for 2 min. The PCR-amplified products were examined by 1.2% agarose gel electrophoresis. The specific target bands were recovered and sequenced by Tsingke (Xi'an, China).

The online BLAST^1^ program from the National Center for Biotechnology Information (NCBI) was used to analyze the cDNA sequence. The amino acid sequence was analyzed with ProtParam^2^ and TMPred^3^ to detect the primary structure and position information of the polypeptide transmembrane region, respectively. TargetP-2.0^4^ and Plant-mPLoc^5^ in Cell-PLoc 2.0 were used to predict the subcellular location of the possible protein structure of the amino acid sequence. The conserved domain was identified by Pfam^6^ and simple modular architecture research tool (SMART^7^). Multiple sequence alignments were performed using DNAMAN8.0 (Lynnon BioSoft, San Ramon, CA, United States). To reveal phylogenetic relationships and potential functional characteristics of targeted proteins from different species, corresponding proteins were collected from different species according to the domain, namely *Arabidopsis thaliana, Oryza sativa, Zea mays, Brachypodium virgatum*, and *Hordeum vulgare*. The phylogenetic relationship was inferred with the neighbor-joining (NJ) method, and a midpoint rooted base tree was drawn in MEGA 7.0 with 1,000 bootstrap iterations (Kumar et al., 2016).

### Virus-Induced Gene Silencing in Wheat

Virus-induced gene silencing, mediated by barley stripe mosaic virus (BSMV), was performed to reveal the function of nine candidate genes during the interaction between wheat M8664-3 and CYR33 (Fitzmaurice et al., 2002; Scofield et al., 2005). The fragment of target gene with *Pac*I and *Not*I was derived from its coding sequence and amplified by RT-PCR (Supplementary Table 1) to construct the BSMV:γ plasmid for gene silencing. After amplification of the target gene fragment and vector linearization, the target gene and linearized vector were recombined, connected, linearized with a restriction enzyme, and transcribed *in vitro* (RiboMAX TM Large-Scale RNA Production System-T7 and Ribo m7G Cap Analog; Promega, Madison, WI, United States) to form a recombinant virus containing the target gene (Petty and Jackson, 1990). BSMV:γ was used as a blank control and γ-PDS as a positive control by friction inoculation, and the second leaf of wheat seedlings was infected with BSMV, with three biological replicates for each gene.

After 24-h incubation in the dark in an artificial climate incubator, the seedlings were placed in a growth chamber at 25°C with 60–80% humidity. When the photo-bleaching phenotype was observed on the BSMV: γ-PDS plants at ~10 dpi, the fourth leaves of γ-gene silencing plants were inoculated with CYR33. Leaf samples were collected at 0, 24, 48, 96, and 120 hpi for qRT-PCR analysis and histological observation (Li et al., 2011). The infection phenotype of *Pst* was observed at ~14 dpi.

### Histological Observation and Statistical Analysis

Inoculated and control leaves of both *Pst* seedling test and VIGS-induced gene silencing were sampled at different time points for histological observation. For each time point, ~15 blades were sampled (Ayliffe et al., 2011). The H_2_O_2_ burst for ROS was observed by DAB (MP Biomedicals, Solon, OH, United States) staining (Xiao et al., 2003; Zou et al., 2018). WGA-Alexa 488 (Invitrogen, Carlsbad, CA, United States) was used to fluorescently stain the *Pst* infestation structure in wheat leaf tissue. The infestation site was determined by the production of germ tubes by *Pst* and the formation of substomatal vesicle in the stomata. For each treatment, 50 infection sites were randomly examined using an Olympus BX-53 microscope (Olympus Corporation, Tokyo, Japan), and the area of ROS, hypha branches, hypha length, and necrotic areas were observed and measured.

The standard error of deviation was calculated using Microsoft Excel. The statistical significance was evaluated by Student's *t* test (*P* < 0.05) using the SPSS software (SPSS, Inc., Chicago, IL, United States).

### Wheat Transformation and Functional Verification

*Agrobacterium tumefaciens* strain EHA105 harboring binary vector, pCAMBIA3301, was used to optimize the transformation system, with ubiquitin promoter and targeted gene replacing cauliflower mosaic virus *35S* promoter and *GUS* gene encoding β-glucuronidase. The ORF of *TaFBN* and *Ta_Pes_BRCT* was amplified separately and inserted into the frame of an expression cassette within the T-DNA region of the pCAMBIA3301 vector digested with *BamHI* and *SpeI*. The construct was verified by DNA sequencing and introduced into *Agrobacterium* EHA105. Then, the *Agrobacterium*-mediated transformation method was applied to genetically transform the target gene (Li et al., 2019). The seeds of 14-day-pollinated immature wheat variety Fielder (scutellum size 1 mm) were treated with 75% alcohol for 30 s and 0.1% HgCl for 10 min, and the immature embryos were then removed with a dissecting needle on an aseptic work table. The immature embryos were infected with the obtained *Agrobacterium*, and then placed on the screening medium and co-cultured in darkness at 23°C for 3 days. Under a microscope, hypocotyls were excised from the contact between the hypocotyl and scutellum of the seeds, and the obtained scutellums were cultured on the screening medium for 14 days. After cutting the callus, a second selection was performed, and the tissue was cultured for 14 days. The healthy callus was transferred to a regeneration medium, and regenerated plantlets were obtained after 7 days. The 786-bp genomic fragment of *TaFBN* and the 1,791-bp genomic fragment of *Ta_Pes_BRCT* were introduced into wheat cultivar Fielder, and the T_0_ generation plants were screened with gene-specific primers by PCR amplification under the follow conditions: 1 min pre-denaturation at 94°C, followed by 30 cycles of denaturation at 94°C for 1 min, annealing at 60°C for 1 min, elongation at 72°C for 45 s, and a final extension step of 10 min at 72°C. Then, the T_2_ generation plants derived from the positive T_0_ plant progeny were inoculated with CYR33 for functional analysis of the target genes.

### Abiotic and Hormone Stress Treatments

In order to evaluate the expression level of *TaFBN* in wheat M8664-3 under hormone perception and environmental stress conditions, seedlings of M8664-3 were divided into eight groups. The first four groups were sprayed with methyl jasmonate (MeJA), ethylene (ET), salicylic acid (SA), or abscisic acid (ABA), at 100 mM each as a hormone treatment and cultivated at 16°C. The last four groups were treated with low temperature (4°C), high temperature (37°C), salt (200 mM NaCl), and drought (15% PEG6000) to experience environmental stress, and sterile water was sprayed at 16°C as a blank control. Leaf samples were collected for the expression level analysis of *TaFBN* 0, 1, 3, 6, 12, and 24 h after the different stimulus treatments.

## Results

### ROS Is Involved in Wheat Cultivar M8664-3 Resistance Against CYR33

**Figure 1:**
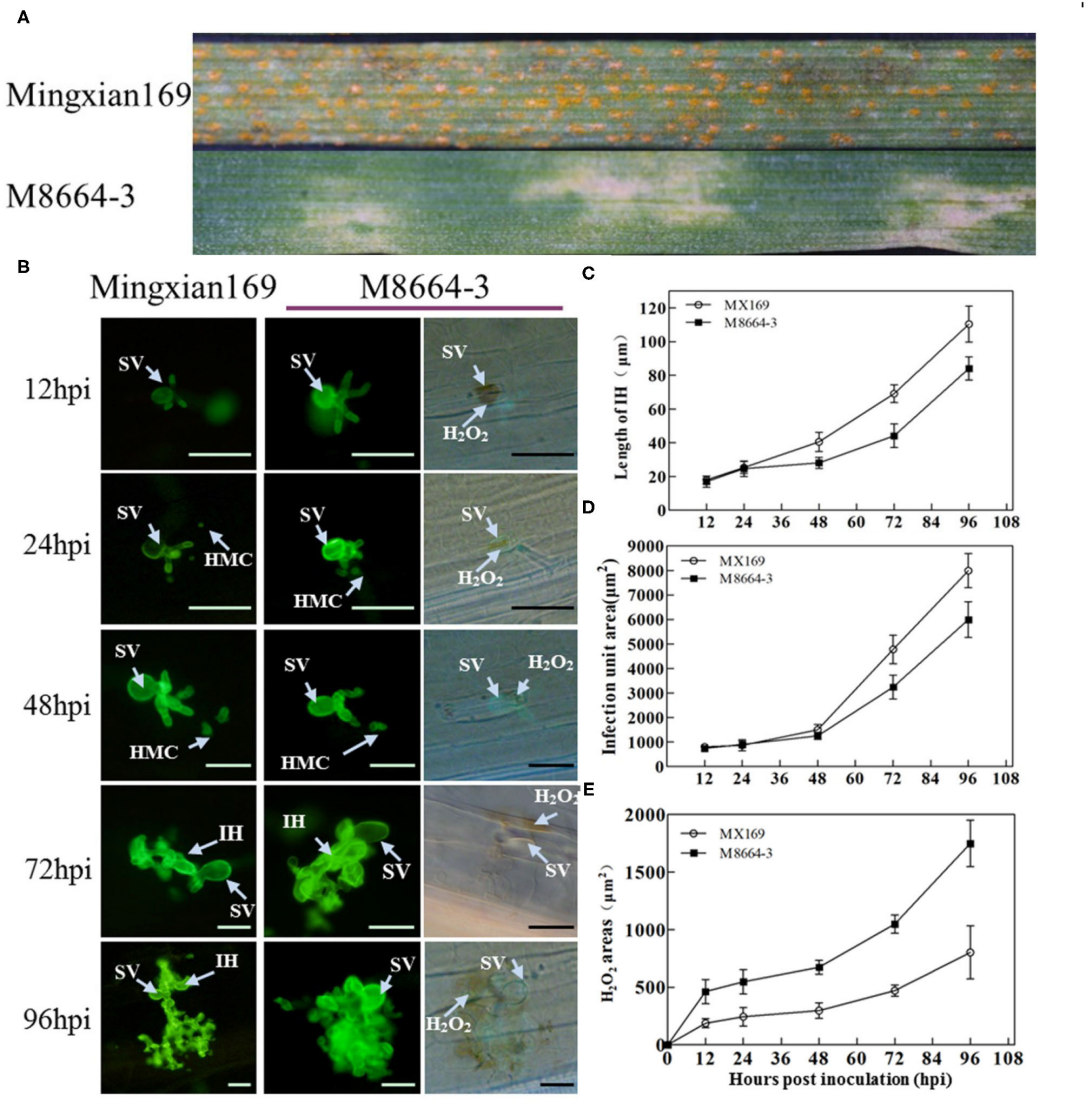
**(A)** Symptoms of wheat-*Leymusmollis* introgression line M8664-3 and susceptible control cultivar Mingxian169 after inoculation with *Puccinia striiformis* f. sp. *tritici* (*Pst*) *race* CYR33. **(B)** Wheat germ agglutinin (WGA) was used to stain the leaves to visualize pathogens. SV, substomatal vesicle; IH, infection hyphae; HMC, haustorial mother cell; ROS, reactive oxygen species. **(C)** Hyphal lengths were measured at different hpi. **(D)** Infection unit area at different hpi. **(E)** H_2_O_2_ area at different hpi. Error bars represent the standard deviations of three independent samples. Bar, 50 μm.

The infection type (IT) of wheat M8664-3 to CYR33 was 2 with large areas of necrosis on the leaves, whereas the IT of susceptible wheat Mingxian169 was 9 with fully expanded uredinium covered on the leaves at 15 dpi (Figure 1A).

Histological observation showed that significant DAB staining appeared at the leaf infection sites at 12 and 96 hpi (Figure 1B). At 12 hpi, strong DAB staining appeared in the guard cells directly contacted by the substomatal vesicle, and there was a significant difference in the H_2_O_2_ staining area of the infestation sites between M8664-3 and Mingxian 169. Between 24 and 48 hpi, the H_2_O_2_ staining area of the M8664-3 and Mingxian169 infection sites continuously increased. Between 72 and 96 hpi, the staining area of the M8664-3 infection site expanded to include guard cells and surrounding areas. In the mesophyll cells of Mingxian169, the DAB staining expansion area was far smaller than that of M8664-3 (Figure 1).

### Expression Analysis of Selected Candidate Genes

**Figure 2:**
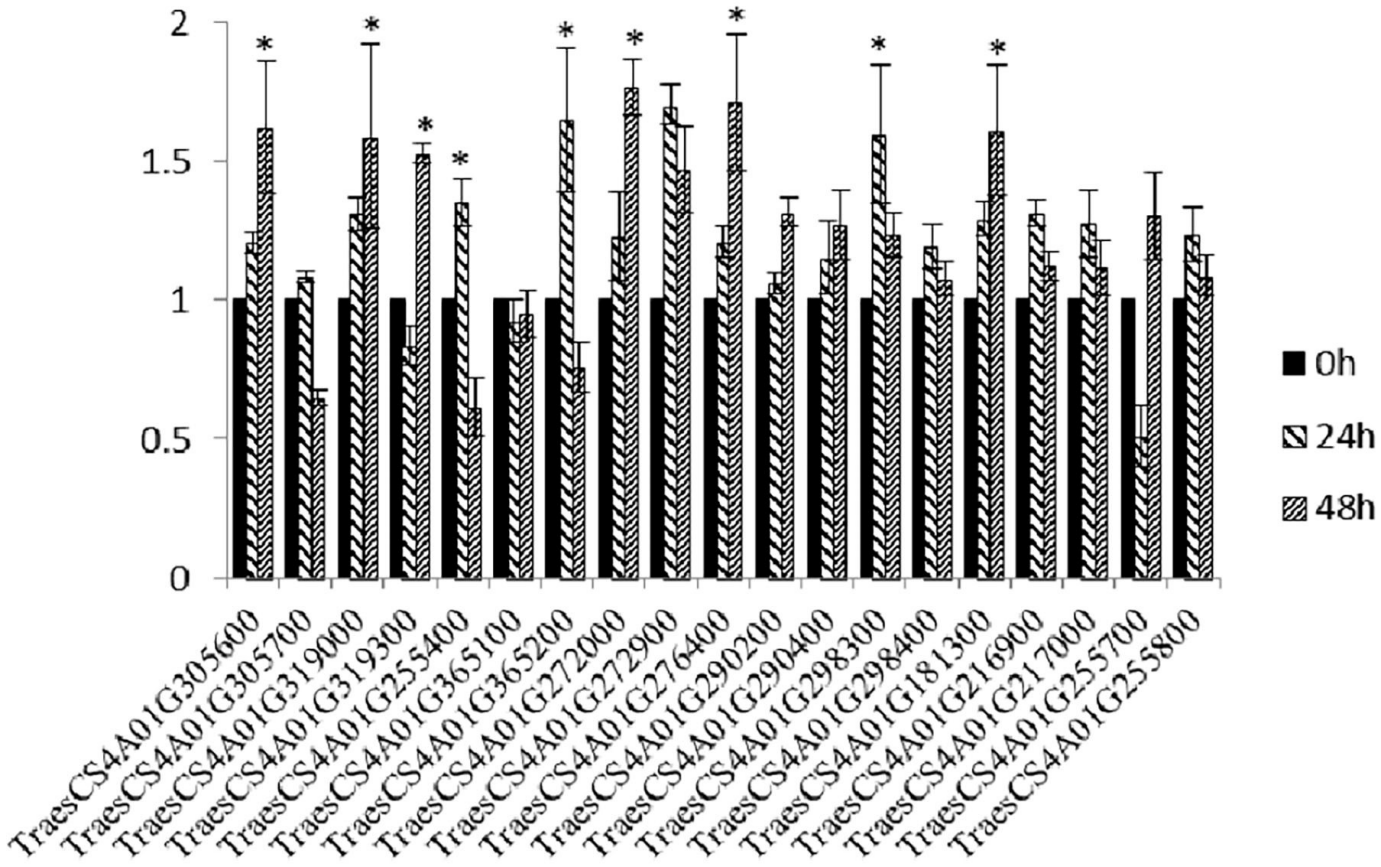
Relative expression levels of 19 candidate genes at 24 and 48 h in M8664-3 after inoculation with *Pst* race CYR33. The data were normalized to the wheat *TaEF-1*α gene. Wheat leaves treated with distilled water were included as a control. Error bars represent the standard deviations of three independent samples. The significance of differences is indicated by asterisks and tested using Student's *t*-test (*P* < 0.05).

Through annotation analysis, 19 genes near the linked markers and containing disease resistance-related domains, or hit by the linked markers of *YrM8664-3* were selected (Supplementary Figure 1; Supplementary Table 2). Among these, nine genes were significantly upregulated after inoculation with CYR33, as determined by qRT-PCR (Figure 2), namely, *TraesCS4A01G305600* (Leucine-rich repeat receptor-like protein kinase family protein), *TraesCS4A01G319000* (PGR5-like protein 1A, chloroplastic), *TraesCS4A01G319300* (disease-resistance protein (NBS-LRR class) family), *TraesCS4A01G365200* (Sn1-specific diacylglycerol lipase alpha), *TraesCS4A01G272000* (plastid-lipid associated protein PAP/fibrillin family protein, which encodes *TaFBN*), *TraesCS4A01G272900* (beta-glucosidase, which encodes *Ta_Pes_BRCT*), *TraesCS4A01G276400* (Pescadillo homolog), *TraesCS4A01G298300* (nonspecific phospholipase C), and *TraesCS4A01G181300* (AP2-like ethylene-responsive transcription factor) (Table 1).

**Table 1:** Nineteen candidate genes and markers linked to resistance gene *YrM86664-3* on chromosome 4AL.

**Position (bp)**	**No. of selected genes/Markers of *YrM8664-3***	**Gene accession**	**Annotation**
415683496–415683891	*BE446584*		
456290064–456292784	C15	TraesCS4A01G181300	AP2-like ethylene-responsive transcription factor
456292321–456292391	*AX111655681*		
515779130–515782750	C16	TraesCS4A01G216900	ABC transporter G family member
516343042–516343722	C17	TraesCS4A01G217000	Late embryogenesis abundant (LEA) hydroxyproline-rich glycoprotein
567479603–567482101	C5	TraesCS4A01G255400	Serine/threonine-protein kinase
567493169–567495275	C18	TraesCS4A01G255700	Ethylene-dependent gravitropism-deficient and yellow-green-like 2
567646583–567646825	*AX109496237*		
567650074–567650374	*AX109001562*		
567650160–567654340	C19	TraesCS4A01G255800	Basic helix-loop-helix (bHLH) DNA-binding superfamily protein
583754800–583757140	C8	TraesCS4A01G272000	Plastid-lipid associated protein PAP / fibrillin family protein
583756221–583756371	*BE403251*		
583949402–583953409	C9	TraesCS4A01G272900	Beta-glucosidase
583949552–583949670	*BE403721*		
584817688–584823187	C10	TraesCS4A01G276400	Pescadillo homolog
584818692–584818992	*AX86179210*		
594162606–594167272	C11	TraesCS4A01G290200	Ankyrin repeat protein-like
594165370–594165403	*BE406959*		
594212051–594212608	*BE591356*		
594213511–594217122	C12	TraesCS4A01G290400	Phosphomethylpyrimidine synthase
597023208–597024827	C13	TraesCS4A01G298300	Non specific phospholipase C
597024543–597025097	*BE637642*		
597052406–597054154	C14	TraesCS4A01G298400	Cation calcium exchanger
600913413–600914954	C1	TraesCS4A01G305600	Leucine-rich repeat receptor-like protein kinase family protein
600917368–600917668	*AX109895154*		
600918067–600918777	C2	TraesCS4A01G305700	Leucine-rich repeat receptor-like protein kinase family protein
607633308–607635652	C3	TraesCS4A01G319000	PGR5-like protein 1A, chloroplastic
607888036–607888131	*BV211529*		
608267651–608271046	C4	TraesCS4A01G319300	Disease resistance protein (NBS-LRR class) family
638240325–638241099	C6	TraesCS4A01G365100	SHAGGY-like kinase
638661695–638661817	*Xgpw2331*		
638791957–638796803	C7	TraesCS4A01G365200	Sn1-specific diacylglycerol lipase alpha

### Silencing of *TaFBN* and *Ta_Pes_BRCT* Weakens Wheat Resistance Against CYR33

**Figure 3:**
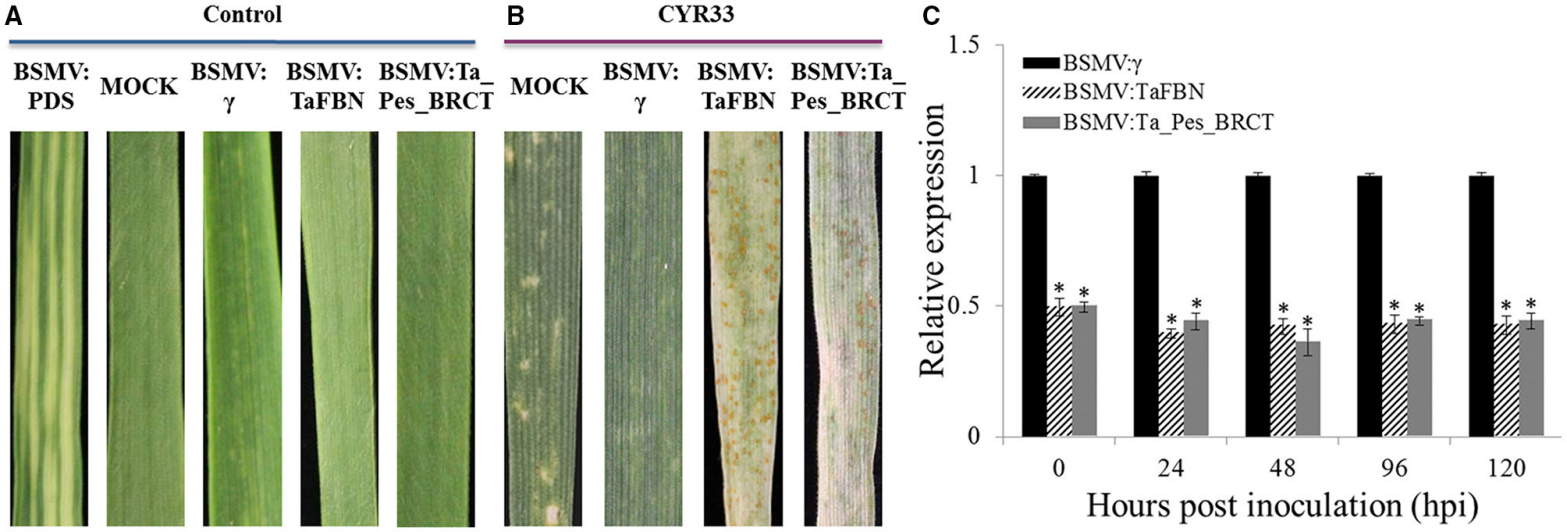
Transient silencing of *TaFBN* and *Ta_Pes_BRCT* with the BSMV-VIGS method. **(A)** Wheat leaves treated with 1× Fes buffer (MOCK) show no phenotypic changes. Mild chlorotic mosaic symptoms were detected on the plants inoculated with BSMV:γ, BSMV:PDS, BSMV:TaFBN, or BSMV:Ta_Pes_BRCT. **(B)** Phenotypes of the fourth leaves infected with uredospores of *Pst* race CYR33 at 10 days post inoculation. **(C)** Silencing efficiency assessment of *TaFBN* in the TaFBN-knockdown plants and *Ta_Pes_BRCT* in the Ta_Pes_BRCT-knockdown plants. Error bars represent the standard deviations of three independent samples. The significance of differences is indicated by asterisks and tested using Student's *t*-test (*P* < 0.05).

Unique fragments were designed to knock down these nine candidate genes using primers specified in Supplementary Table 1. All of the BSMV-inoculated plants displayed chlorotic mosaic symptoms at 10 dpi, but there were no obvious defects in further leaf growth, while the leaves inoculated with BSMV: TaPDS exhibited photobleaching (Figure 3A), indicating that the BSMV induced gene silencing system functions well. After knocking down these genes with the VIGS method, M8664-3 became susceptible in the *TaFBN-* and *Ta_Pes_BRCT-*silenced system (Figure 3B), which indicated that these two genes may be involved in the resistance of M8664-3 to *Pst*. To determine the efficiency of VIGS, qRT-PCR was performed to examine the relative transcript levels of *TaFBN* and *Ta_Pes_BRCT* in the fourth leaves of infected plants. Compared with control inoculations, transcript levels of *TaFBN-*knockdown plants were reduced by 49, 39, 42, 43, and 42% at 0, 24, 48, 96, and 120 hpi, and *Ta_Pes_BRCT* knockdown plants also showed a stable efficiency by reducing to 49, 44, 36, 44, and 44% at 0, 24, 48, 96, and 120 hpi with CYR33, respectively (Figure 3C).

**Figure 4:**
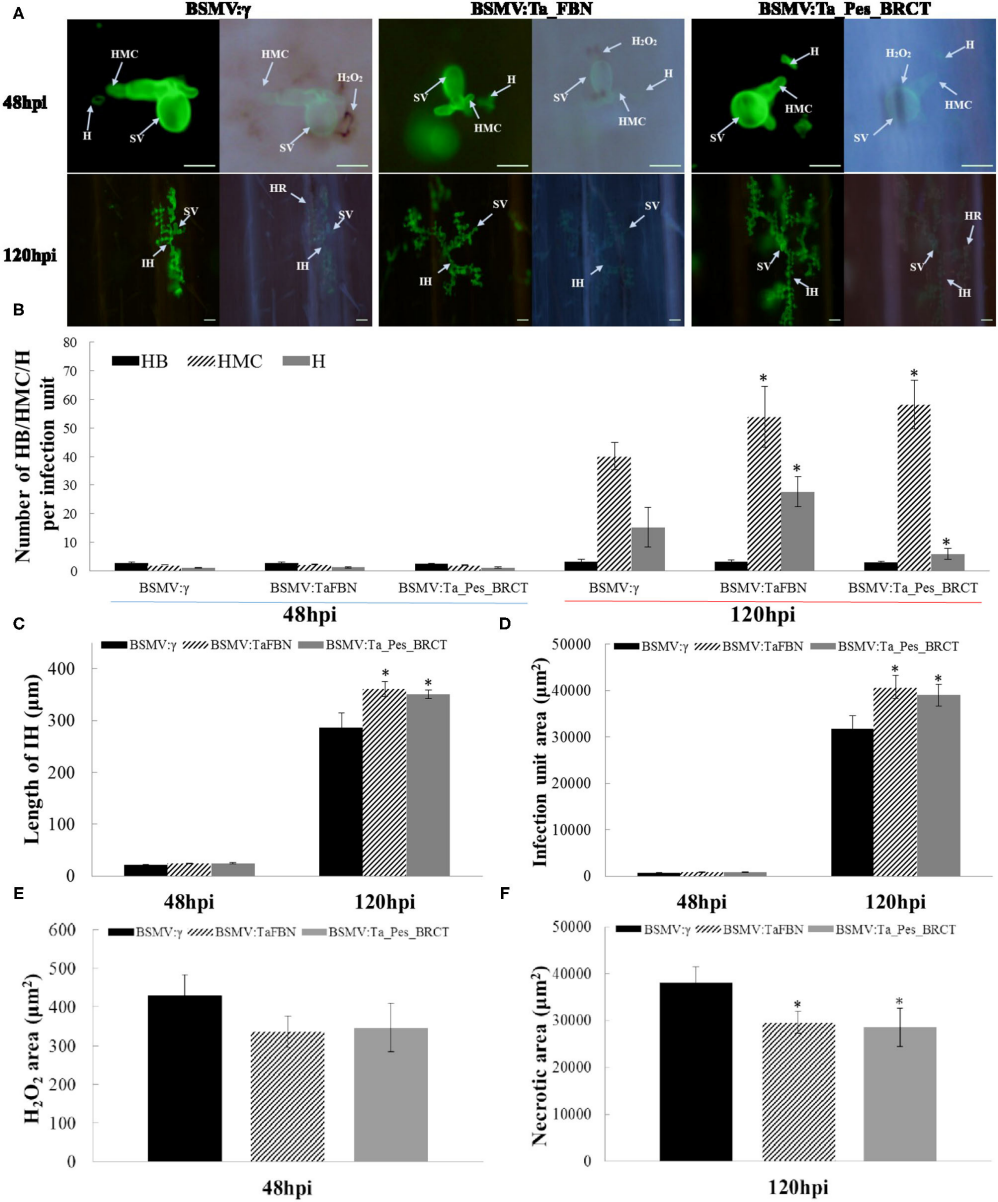
Histological observations of *Pst* race CYR33 infection in TaFBN-knockdown and Ta_Pes_BRCT-knockdown wheat plants. **(A)** WGA was used to stain the leaves to visualize pathogens. **(B)** Number of HB/HMC/H; **(C)** length of IH; **(D)** infection unit area; **(E)** H_2_O_2_ area; and **(F)** necrotic area staining by 3,3′-diaminobenzidine (DAB) were measured with DP-BSW software in *TaFBN*-knockdown plants at 48 and 120 hpi after inoculation. SV, substomatal vesicle; IH, initial hyphae; HMC, haustorial mother cell; SH, secondary hyphae. Error bars represent the standard deviations of three independent samples. The significance of differences is indicated by asterisks and tested using Student's *t*-test (*P* < 0.05). Bar, 50 μm.

Histological observations showed that the number of haustorial mother cells, hypha length, and *Pst* growth area in *TaFBN-* and *Ta_Pes_BRCT*-silenced leaves all slightly increased as compared with that of the unsilenced treatment 48 hpi, but the DAB-reactive oxygen staining area did not significantly change, and no necrotic cells were found. At 120 hpi, the area of *Pst* growth, length of hyphae, and number of hyphae branches, haustorial mother cells, and haustorium in *TaFBN*- and *Ta_Pes_BRCT*-silenced leaves were all increased, and the difference was extremely significant. DAB-reactive oxygen staining decreased to 0, and the area of necrotic cells also significantly decreased compared with the unsilenced treatment at 120 hpi (Figure 4). In summary, the silencing of *TaFBN* and *Ta_Pes_BRCT* reduced disease resistance and made plants more susceptible to *Pst*.

### *TaFBN* or *Ta_Pes_BRCT* Encodes Proteins Related to Defense Response

**Figure 5:**
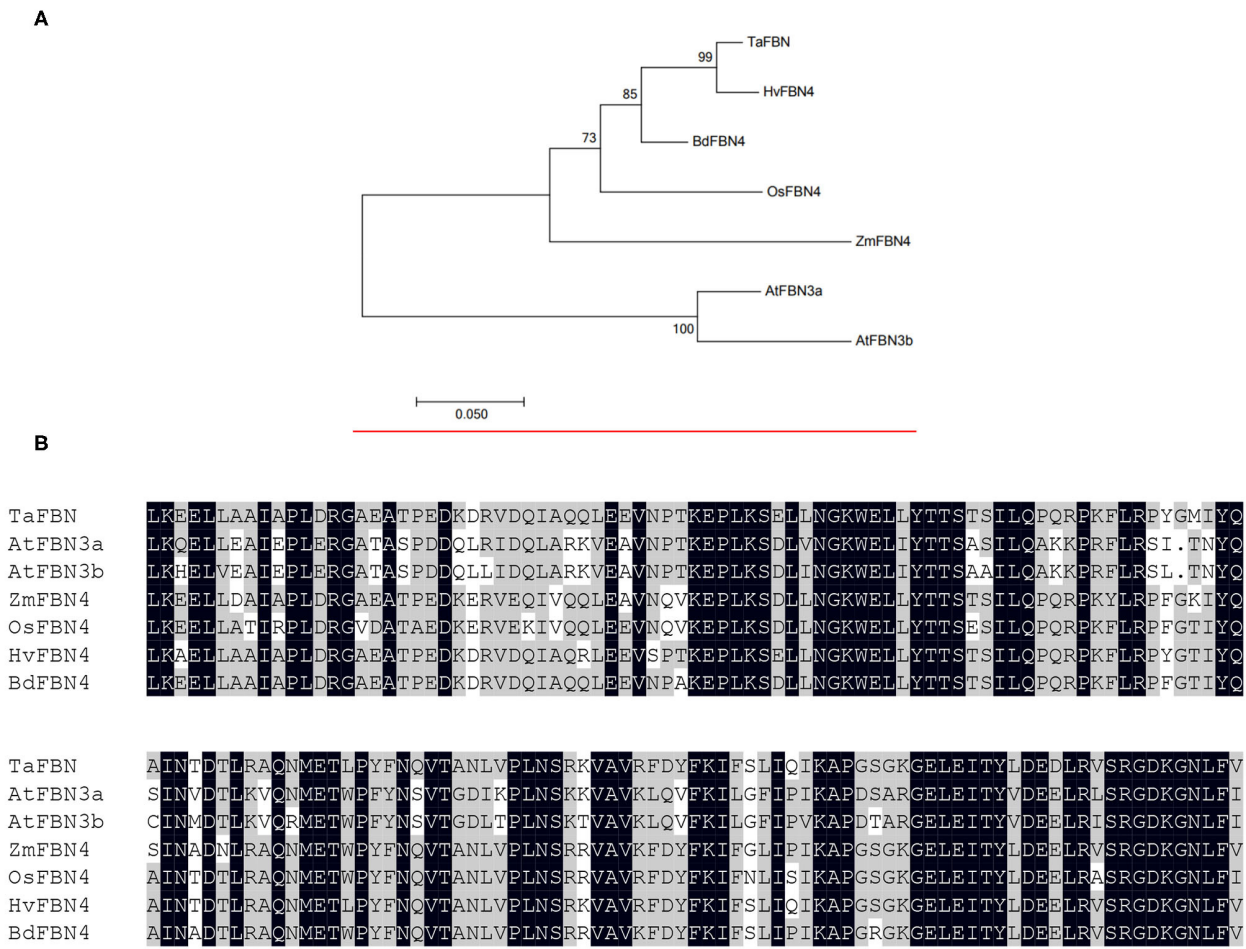
**(A)** Phylogenetic analysis of fibrillin (FBN) proteins. A neighbor-joining tree of FBN in *Triticuma estivum* (TaFBN, KAF7095220), *Hordeum vulgare* (HvFBN4, KAE8820120)*, Brachypodium distachyon* (BdFBN4, XP_003560711), *Oryza sativa* (OsFBN4, XP_015632312), *Arabidopsis thaliana* (AtFBN3a/AT3G26070, NM_113511; AtFBN3b/At3g26080, BT020596), and *Zea mays* (ZmFBN4, ACG27798). **(B)** Multiple amino acid sequence alignments of TaFBN with OsFBN4, ZmFBN4, AtFBN3a, AtFBN3b, and HvFBN4. Amino acid identity (black boxes) and similarity (gray boxes) are shown within the protein kinase domain.

*TaFBN* encodes a protein composed of 261 amino acids, with a molecular weight of 28.59 kDa, an isoelectric point (PI) of 9.34, and an average hydrophobicity of −0.330, which suggested that it may be a hydrophilic protein. There was a PAP_fibrillin domain at positions 90–251 of the amino acid; therefore, it was temporarily named *TaFBN*. The phylogenetic analysis of *TaFBN* with *H. vulgare* (*HvFBN4*, KAE8820120)*, Brachypodium distachyon* (*BdFBN4*, XP_003560711), *O. sativa* (*OsFBN4*, XP_015632312), *A. thaliana* (*AtFBN3a*, NM_113511; *AtFBN3b*, BT020596), and *Z. mays* (*ZmFBN4*, ACG27798) resulted in the clustering of *TaFBN* with *HvFBN4, BdFBN4, OsFBN4*, and Zm*FBN4*, all of which are members of FBN4 proteins in monocotyledons (Figure 5A). A nucleic acid sequence analysis revealed that *TaFBN* shared 96.55% identity with *HvFBN4* from *H. vulgare*. Multiple amino acid sequence alignments of *TaFBN* with *HvFBN4, BdFBN4, OsFBN4*, and *ZmFBN4* showed that *TaFBN4* is predicted to encode proteins with the unique conserved domains of PAP complex FBN4 (Figure 5B). Therefore, it was determined that *TaFBN* and FBN4 were clustered together, and that the functional annotations of the latter on the UniProt website were related to resistance to bacterial diseases and ozone.

*Ta_Pes_BRCT* encodes a protein composed of 596 amino acids, with a molecular weight of 68.32 kDa, PI of 7.68, and an average hydrophobicity of −0.615, which indicated that it may be a hydrophilic protein. The amino acid coded *by Ta_Pes_BRCT* has a Pescadillo (PES) domain at positions 9–277, and the 339–417 amino acids contain a BRCT domain. The predicted function may be related to ribosomes; therefore, it was temporarily named *Ta_Pes_BRCT*. The phylogenetic analysis and multiple amino acid sequence alignment of Pes_BRCT proteins indicated that *Ta_Pes_BRCT* is predicted to encode proteins with conserved domains of the Pes_BRCT complex (Supplementary Figure 2). The functional annotations of BRCT on the UniProt website were related to DNA repair under stress. As predicted by the TMpred program, *TaFBN* and *Ta_Pes_BRCT* have no transmembrane domain. As predicted by TargetP-2.0 and Plant-mPLoc, the location of the protein encoded by *Ta_FBN* was predicted in Chloroplast; the location of the protein encoded by *Ta_Pes_BRCT* was predicted in Nucleus.

### Functional Verification of Transgenic Plants

**Figure 6:**
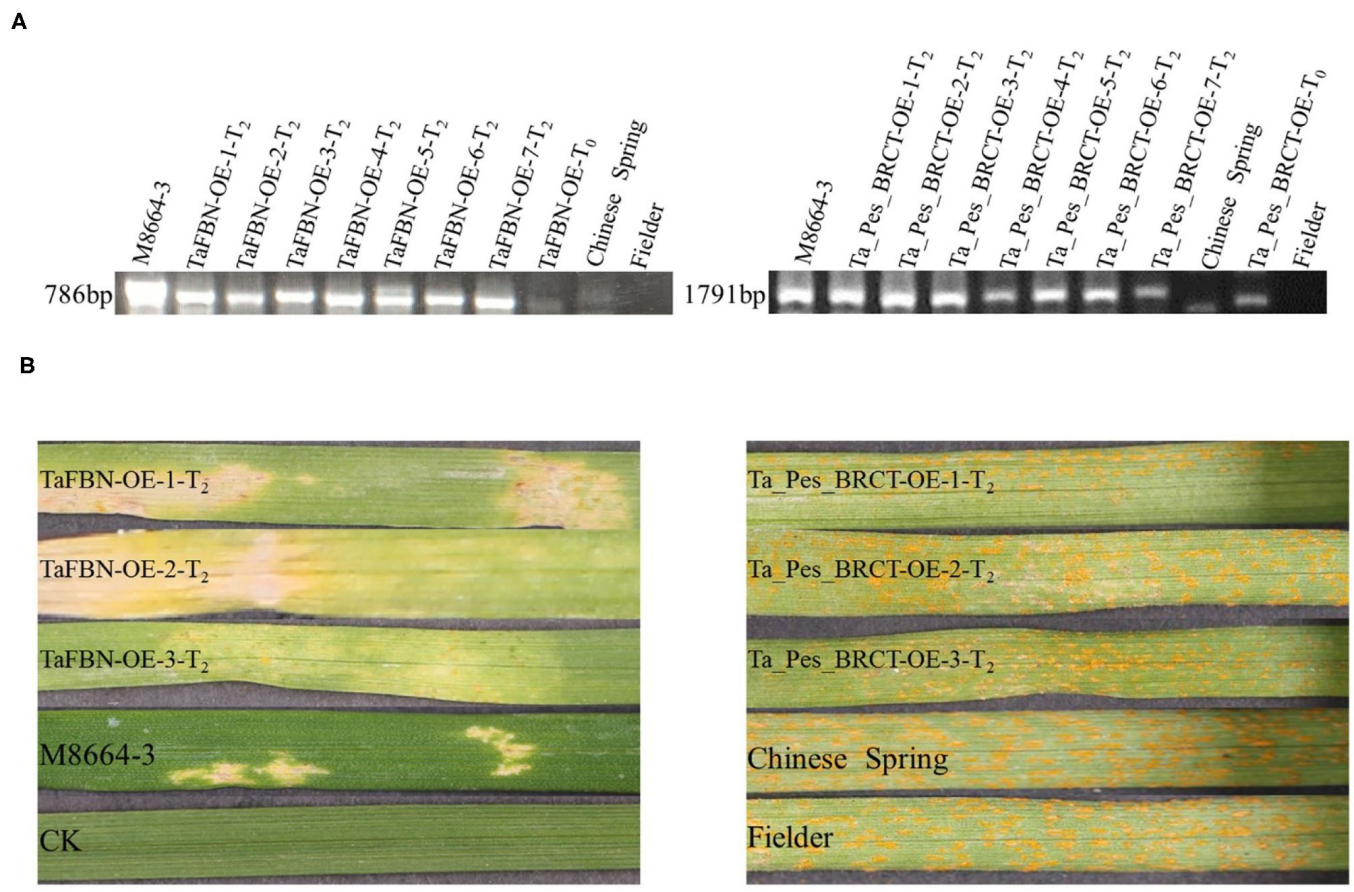
Stable transformation of the susceptible bread wheat cultivar Fielder with *TaFBN* confers resistance to *Pst* race CYR33. **(A)** Identification by PCR of individuals transformed with *TaFBN* and *Ta_Pes_BRCT*. Wild-type Fielder was used as a negative control; M8664-3 containing the full sequence of *TaFBN* and *Ta_Pes_BRCT* was used as a positive control. **(B)** Fielder T_2_ plants transformed with *TaFBN* were resistant to CYR33 but those transformed with *Ta_Pes_BRCT* were susceptible to CYR33. Wild-type Fielder, Chinese Spring, and M8664-3 were used as susceptible and resistant controls.

In order to further analyze the function of *TaFBN* and *Ta_Pes_BRCT* in stripe rust resistance, the two genes were introduced into the susceptible bread wheat variety Fielder by an *Agrobacterium*-mediated transformation method. The individuals of T_0_ generation were identified by PCR analysis (Figure 6A), and seven independent T_2_ lines obtained from the positive T_0_ progeny were further used to conduct disease resistance tests and PCR detection procedures. Three replicates were tested for each line. The T_2_ generation of *TaFBN* transgenic plants was resistant to CYR33, while all of the *Ta_Pes_BRCT* transgenic plants were susceptible to CYR33 (Figure 6B), which indicated that *TaFBN* confers more important resistance to CYR33.

### *TaFBN* Responds to Abiotic Stress and Hormone Treatments

**Figure 7:**
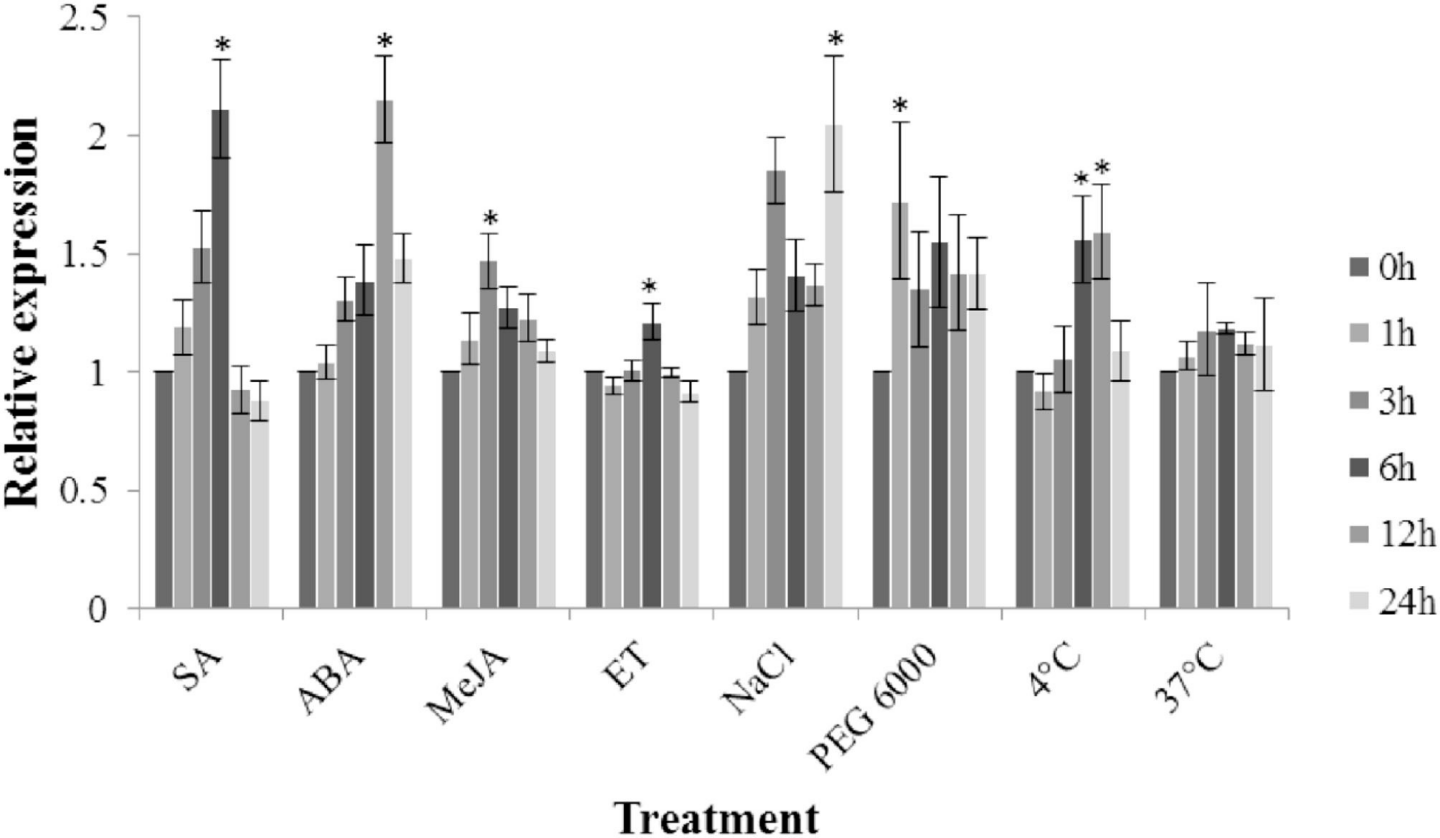
Expression analysis of *TaFBN* under different hormones and abiotic stresses. SA, salicylic acid; ABA, abscisic acid; MeJA, methyl jasmonate; ET, ethylene. Error bars represent the variations among three independent replicates. The data were normalized to the TaEF-1α gene. Wheat leaves treated with distilled water were included as a control. Error bars represent the standard deviations of three independent samples. The significance of differences is indicated by asterisks and tested using Student's *t*-test (*P* < 0.05).

Under different hormone treatments (MeJA, ET, SA, and ABA), the transcription level of *TaFBN* in the leaves of M8664-3 wheat seedlings was determined (Figure 7). The expression level of *TaFBN* significantly increased under SA and ABA treatments, and peaked (more than twice) at 6 and 12 hpi, respectively. The expression of *TaFBN* also slightly increased after MeJA and ET treatment. Under abiotic stress, the expression of *TaFBN* significantly increased after NaCl, PEG6000, and 4°C treatment, but its expression in 37°C-high temperature treatment did not significantly increase. In summary, *TaFBN* might be induced by SA, ABA, high salt, drought, and low temperature to increase its expression.

## Discussion

In our previous study, *YrM8644-3* was located in bin 4AL13-0.59-0.66 near 4AL12-0.43-0.59 on wheat chromosome 4A. However, it was challenging to clone this gene because of *YrM8664-3* derived from the wheat-*L. mollis* introgression line M8664-3 and the complexity of the wheat hexaploid genome. Fortunately, the continuous improvement in the whole genome sequencing of wheat “Chinese Spring” provided great convenience for gene cloning and functional analysis (Avni et al., 2017; IWGSC et al., 2018).

In this study, based on the chromosome location of *YrM8664-3* in our previous study, the sequences of the linked markers of *YrM8664-3* were assigned against the Chinese Spring IWGSC RefSeq V1.0 Reference Genome (IWGSC et al., 2018), and then qRT-PCR was performed to analyze the expression level of candidate genes under *Pst* infection. Among the 19 selected genes that are near the linked markers and containing resistance domains or just hit by linked markers of *YrM8664-3*, the expression level of nine genes were significantly enhanced after inoculation with CYR33. The VIGS system was used to characterize gene function, and only two candidate genes, *TaFBN* and *Ta_Pes_BRCT*, silencing plants were found to be significantly weakened in disease resistance to CYR33. VIGS is a technique for rapid gene function analysis based on the principle of specific degradation of endogenous mRNA sequences caused by post-transcriptional gene silencing (PTGS). BSMV-VIGS is widely used in the rapid analysis of gene function of monocotyledonous plants, particularly barley and wheat. Feng et al. (2015) selected six unigenes from transcriptome analysis and achieved transient silencing of the six unigenes individually through VIGS using the BSMV vector. The results showed that the six unigenes inhibited the vernalization of wheat, and that during silencing or down-regulation, the genes promoted flower development in wheat. Liu et al. (2020) used transient expression and BSMV-mediated *TabHLH49* gene silencing, and discovered that *TabHLH49* positively regulated WZY2 dehydrogenase expression and increased the resistance of wheat to drought.

The most direct and effective verification method for gene function is transgenic technology. By transferring the *HvBADH1* gene from *H. vulgare* into *T. aestivum via* traditional *Agrobacterium tumefaciens*-mediated transformation, Li et al. (2019) found that the overall salt tolerance of target plants was significantly improved, and that the damaging effect of high salt was significantly reduced after overexpression of the *HvBADH1* gene. In cereals, *Agrobacterium*-mediated transgenic sites are generally considered to be cleaner, with fewer copies and rearrangements than biologically generated transgenic sites (Wu et al., 2006). Horvath et al. (2003) subjected the stem-rust-susceptible barley cv. Golden Promise to *Agrobacterium*-mediated transformation with the *Rpg1* gene, and characterized their seedling infection response to pathotype *Pgt*-MCC of the stem rust fungus. This demonstrated that susceptible barley can become resistant by transformation with a cloned resistant gene. In this study, the *TaFBN* and *Ta_Pes_BRCT* genes were transformed into the susceptible variety Fielder by *Agrobacterium*-mediated transformation. The *TaFBN* transgenic plants exhibited obvious resistance after inoculation with CYR33, which indicates that *TaFBN* may be involved in stripe rust resistance in M8664-3.

TaFBN has a conserved fibrillin (FBN) domain that was named fibrils, because those related proteins were first detected in fibrils in the chromoplasts of *Rosa rugosa* and *Capsicum annuum* fruit (Newman et al., 1989; Deruere et al., 1994; Kim et al., 2015). FBN proteins participate in a variety of important biological functions, in addition to photosynthesis and structural roles, which also respond to numerous abiotic and biotic stresses, especially oxidative stress (Youssef et al., 2010; Kim et al., 2015). Leitner-Dagan et al. (2006) showed that the Chrc (FBN1) in cucumber leaves was induced by *Sphaerotheca fuliginea* and *LeChrc* (FBN1) in tomato plants infected with *Botrytis cinerea*. *LeChrc* expression is a necessary condition for resistance to *B. cinerea*. Using the tomato plant system, transgenic plants with *LeChrc* inhibition were more susceptible to infection both *in vitro* with isolated leaves and in growth chambers with intact leaves and stems (Cooper et al., 2003; Leitner-Dagan et al., 2006). Similarly, studies (Singh et al., 2010; Jiang et al., 2020) have shown that both Arabidopsis and apple *FBN4* gene T-DNA insertion mutants are more sensitive to bacterial infections *Pseudomonas syringae* pathovar tomato and *Erwinia amylovora*, respectively. In Arabidopsis, pathogen-associated molecular pattern (PAMP) induces the phosphorylation of *FBN4*, and it is speculated that *FBN4* may be involved in plant disease resistance response.

During plant growth and development stages, when plants are under abiotic stresses (such as drought, cold, heat, bright light, and wound management) or hormone induction (with gibberellin, jasmonic acid, and abscisic acid), the expression of fibrillins is varied and complex (Pruvot et al., 1996; Kuntz et al., 1998; Langenkamper et al., 2001; Leitner-Dagan et al., 2006; Simkin et al., 2008). When red pepper fruits were treated with gibberellic acid, FBN1 mRNA and protein levels decreased (Deruere et al., 1994). Conversely, the FBN1 and FBN2 proteins are involved in the jasmonate biosynthesis pathway in Arabidopsis in response to light and cold stress (Youssef et al., 2010). In addition, when tomato flacca mutant plants were subjected to drought stress, ABA biosynthesis was defective, and FBN protein accumulation decreased, because ABA treatment can induce FBN protein levels (Gillet et al., 1998). Herein, we observed an induction of *TaFBN* upon SA and ABA treatment, suggesting that *TaFBN* may be an effector associated with the SA and ABA signaling pathways. Taken together with our observation of enhancement of *TaFBN* expression in response to environmental stress stimuli (high salt, drought, cold, heat), it is reasonable to hypothesize that *TaFBN* functions at the nexus of biotic and abiotic stress pathways.

BRCT motifs were originally identified in the breast cancer tumor suppressor protein BRCA1 by Koonin et al. (1996), and now have been identified in numerous proteins involved in DNA repair and cell cycle checkpoints (Mathilde et al., 2003). Roy et al. (2015) indicated the importance of BRCT in regulating the stability of proteins under genotoxic stress in plants. *Pst*-infected wheat may produce oxygen-free radicals, such as O^2−^ and H_2_O_2_, that damage cells, which subsequently produce metabolic byproducts that cause DNA base damage. *Ta_Pes_BRCT* is a BRCT-domain-containing protein that may be involved in DNA repair and that could be induced to up-regulate by *Pst*. However, after transgenic verification, it was found that *Ta_Pes_BRCT* is a related gene in the process of wheat resistance to *Pst* that plays less important role than *TaFBN*.

In summary, this study selected candidate genes of *YrM8664-3* by bioinformatics analysis, and verified the resistant function of the candidate genes *TaFBN* and *Ta_Pes_BRCT* by qRT-PCR, BSMV-VIGS, and genetic transformation. Finally, it was validated that *TaFBN* may be involved in *YrM8664-3* stripe rust resistance as an important gene *via* the SA and ABA signaling pathways.

## Data Availability Statement

The original contributions presented in the study are included in the article/Supplementary Materials, further inquiries can be directed to the corresponding author/s.

## Author Contributions

BW and QL designed the experiments. PJ, KC, JL, and ZW performed the experiments and analyzed the data. PJ, JL, KC, ZW, PC, QL, and BW wrote the manuscript. All authors contributed to the article and approved the submitted version.

## Funding

This research was supported by the National Key R&D Program of China (Grant Nos: 2018YFD0200403 and 2018YFD0200501), the Open Project Program of State Key Laboratory of Crop Stress Biology for Arid Areas, NWAFU, Yangling, Shaanxi, 712100, China (CSBAA2019007), the Technical Guidance Project of Shaanxi Province (Grant No: 2017CGZH-HJ-01), the National Science Foundation of China (Grant No: 31501620), and the China Ministry of Education 111 Project (Grant No: B07049).

## Conflict of Interest

The authors declare that the research was conducted in the absence of any commercial or financial relationships that could be construed as a potential conflict of interest.

## Publisher's Note

All claims expressed in this article are solely those of the authors and do not necessarily represent those of their affiliated organizations, or those of the publisher, the editors and the reviewers. Any product that may be evaluated in this article, or claim that may be made by its manufacturer, is not guaranteed or endorsed by the publisher.

## Footnotes

^1^ https://blast.ncbi.nlm.nih.gov/Blast.cgi

^2^ https://web.expasy.org/protparam/

^3^ http://sbcb.bioch.ox.ac.uk/TM_noj/TM_noj.html

^4^ http://www.cbs.dtu.dk/services/TargetP-2.0/

^5^ http://www.csbio.sjtu.edu.cn/bioinf/plant-multi/

^6^ http://pfam.xfam.org/

^7^ http://smart.embl-heidelberg.de/
