# Corrigendum: Functional Verification of Two Genes Related to Stripe Rust Resistance in the Wheat-*Leymus mollis* Introgression Line M8664-3

**DOI:** 10.3389/fpls.2021.803911

**Published:** 2021-11-19

**Authors:** Pengfei Jin, Kaixiang Chao, Juan Li, Zihao Wang, Peng Cheng, Qiang Li, Baotong Wang

**Affiliations:** ^1^State Key Laboratory of Crop Stress Biology for Arid Areas, College of Plant Protection, Northwest A&F University, Yangling, China; ^2^College of Chemistry, Biology and Environment, Yuxi Normal University, Yuxi, China; ^3^Dingxi Plant Protection and Quarantine Station, Dingxi, China

**Keywords:** functional verification, stripe rust, resistance, wheat-*Leymus mollis*, *YrM8664-3*

In the original article, the fourth image in Figure 3B (BSMV:Ta_Pes_BRCT) was incorrect when published. The corrected version of Figure 3 appears below.

**Figure 3:**
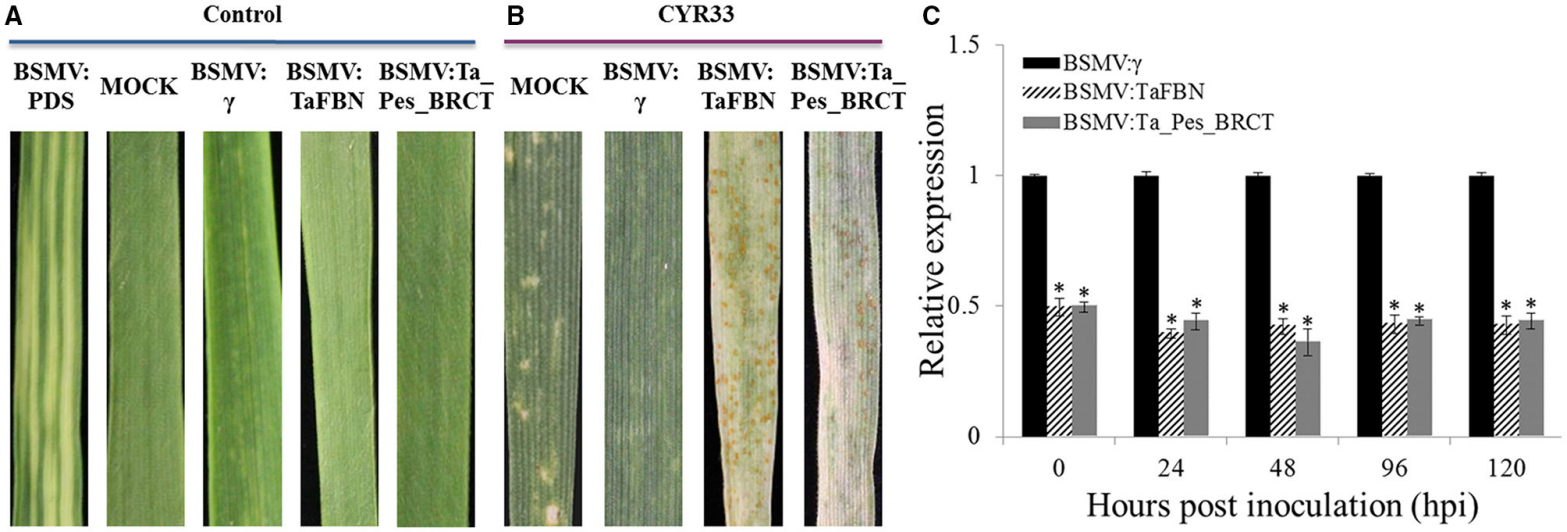
Transient silencing of *TaFBN* and *Ta_Pes_BRCT* with the BSMV-VIGS method. **(A)** Wheat leaves treated with 1× Fes buffer (MOCK) show no phenotypic changes. Mild chlorotic mosaic symptoms were detected on the plants inoculated with BSMV:γ, BSMV:PDS, BSMV:TaFBN, or BSMV:Ta_Pes_BRCT. **(B)** Phenotypes of the fourth leaves infected with uredospores of *Pst* race CYR33 at 10 days post inoculation. **(C)** Silencing efficiency assessment of *TaFBN* in the TaFBN-knockdown plants and *Ta_Pes_BRCT* in the Ta_Pes_BRCT-knockdown plants. Error bars represent the standard deviations of three independent samples. The significance of differences is indicated by asterisks and tested using Student's *t*-test (*P* < 0.05).

The authors apologize for this error and state that this does not change the scientific conclusions of the article in any way. The original article has been updated.

## Publisher's Note

All claims expressed in this article are solely those of the authors and do not necessarily represent those of their affiliated organizations, or those of the publisher, the editors and the reviewers. Any product that may be evaluated in this article, or claim that may be made by its manufacturer, is not guaranteed or endorsed by the publisher.

