# Supplementary material for: Functional Verification of Two Genes Related to Stripe Rust Resistance in the Wheat-Leymus mollis Introgression Line M8664-3
Source: Front Plant Sci. 2021 Oct 25;12:754823. doi: 10.3389/fpls.2021.754823 (PMC8574815; doi:10.3389/fpls.2021.754823)
Supplement: Supplementary file 1 [file Data_Sheet_1.pdf]

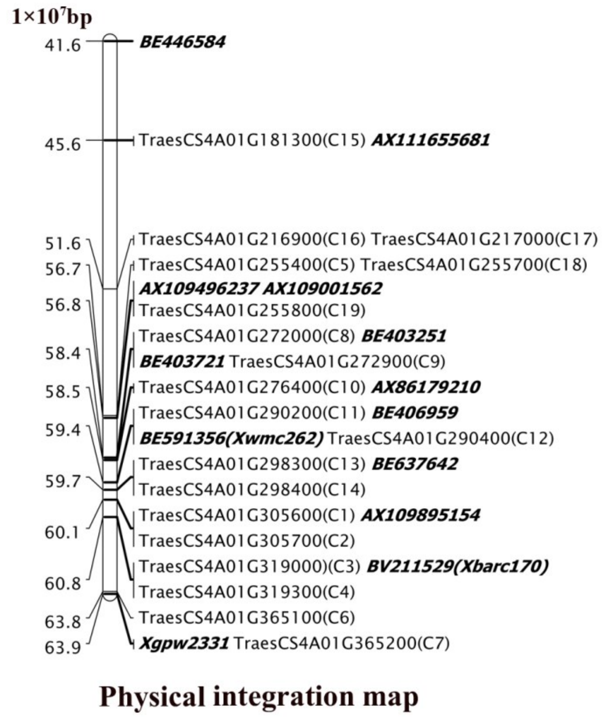

Supplementary Figure S1. Physical integration map of candidate genes and markers linked to resistance gene *YrM86664-3*. Markers and candidate genes are shown on the right and their position on chromosome 4A of the IWGSC-RefSeq database are shown on the left. The numeric unit is 1 × 10^7 bp.

A

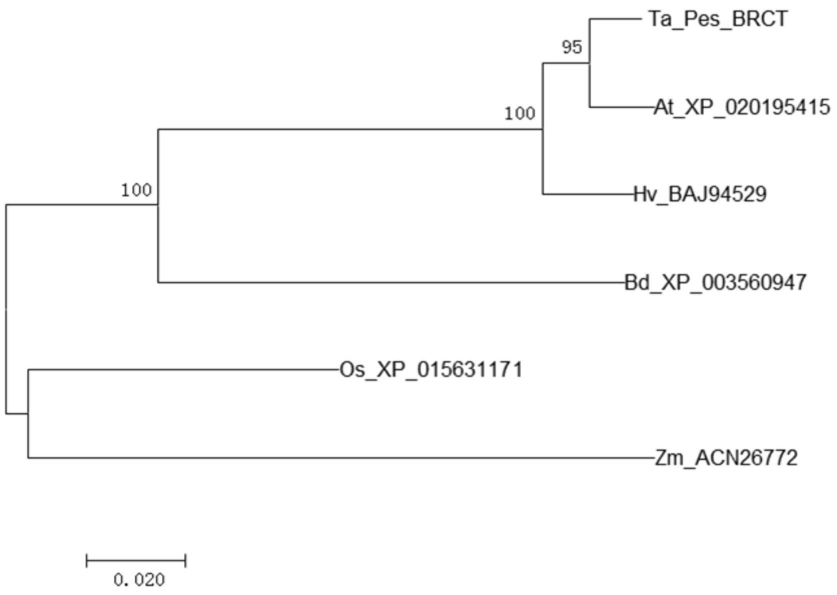

B

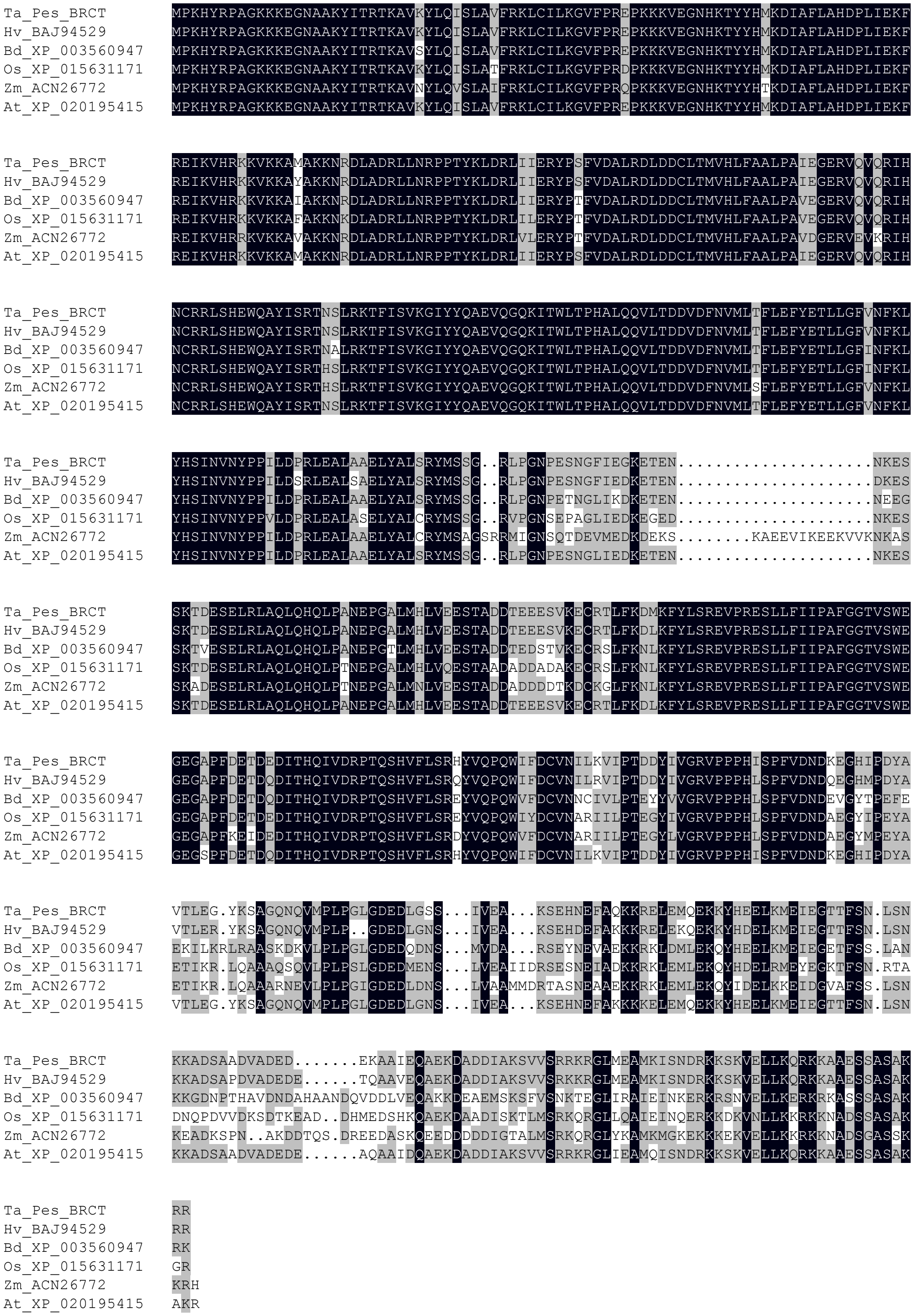

Supplementary Figure S2. (A) Phylogenetic analysis of Pes_BRCT proteins. A neighbor-joining tree of Pes_BRCT in *Triticum aestivum* (Ta_Pes_BRCT, KAF7044273), *Arabidopsis thaliana* (At_XP_020195415, XP_020195415), *Hordeum vulgare* (Hv_BAJ94529, BAJ94529), *B. distachyon*

(Bd_XP_003560947, XP_003560947), *Oryza sativa* (Os_XP_015631171, XP_015631171), and *Zea mays* (Zm_ACN26772, ACN26772). (B) Multiple amino acid sequence alignment of Ta_Pes_BRCT with At_XP_020195415, Hv_BAJ94529, Bd_XP_003560947, Os_XP_015631171, and Zm_ACN26772. Amino acid identity (black boxes) and similarity (gray boxes) are shown within the protein kinase domain.

2.2 Supplementary Tables

Supplementary Table S1. Primers used in this study.

	**Gene**	qPCR_F	qPCR_R	VIGS_F	VIGS_R	complete sequence_F	complete sequence_R
**C1**	TraesCS4A01G305600	GTCTCGGCAATAATAAACT	CTAAGCCACTCTGGAAAA	TTCCCAACTCGGTAATCT	TTCGGTATCAAACCCACA	AAGACTTGGGAATAGCAT	AAGAAGATAATCAATGCG
**C2**	TraesCS4A01G305700	TTTCCGTAACCAAATCTC	TTGTAAAGTTGCATGTCTT				
**C3**	TraesCS4A01G319000	ATGGAAGCGACATCGTGAC	CGGAACTGGAATACATTGACCT	CGAGCAGCAAGGGCAGGT	GCAGGAACATCTTCAAGTAGTCAA		
**C4**	TraesCS4A01G319300	ATCAGCAGTTATCCAGCGT	ATCAGCGACCTCGGTTATG	TGCTTCGCTTTCTGTTCG	GCATATTTGTCAGCACCTC		
**C5**	TraesCS4A01G255400	TGCGAGGCCAAGAAGAGCG	CGACCAGTACCCGTCCACCA				
**C6**	TraesCS4A01G365100	TACAGTAATGCCAAACAGG	GACTCCAGGAACAGTATGAA				
**C7**	TraesCS4A01G365200	TCACAAGGGGATGTTGAAAGC	AGGGAAAAGTTGGCGCAGT	CGTTCTCATCCACAAGTCTA	TCATACAGGCACAGGACC		
**C8**	TraesCS4A01G272000	GCCATACGGGATGATTTAC	GCCACTTCCAGGTGCTTT	GCCGCCTCCTCGTCTTCC	GCTCCCACTTGCCGTTGA	ATGGCGCTGGTCTCGCTC	TTACAACGGAACTCGGTATAATGG
**C9**	TraesCS4A01G272900	AACTCAGCAACAGAGCCTTACA	AGTGAACCTGGGCAACCT	ACCAACTCAACTGAAGACCAAGCA	ACATCCCCGACGGCACGA		
**C10**	TraesCS4A01G276400	GGAGGAACTGTTTCGTGGG	GGTTGAACATAATGCCTGGAG	CGCCGTCTTCAGAAACTA	ATGTGGAGTAAGCCAAGTG	ATGCCGAAGCACTACCGCC	TCAGCGACGTTTGGCAGAA
**C11**	TraesCS4A01G290200	GACAGTGATTCGTCGGATAACAA	CCACCGCACAAGCCAAGGG				
**C12**	TraesCS4A01G290400	AGTAGCAGAGCCAGCAAAACC	GCTCCGTGGGAAGCAATCT				
**C13**	TraesCS4A01G298300	GGCAAGGACGGGTCTCACA	CGCAAAGCAAGCCAGCATC	AAAGCCGTCCCCTTCCGC	GGTAATAGACCCCGAAGGAATG		
**C14**	TraesCS4A01G298400	TGGTGGGGCTGGGGCTAT	AAGAAGGTGAGGTAGATGACGATG				
**C15**	TraesCS4A01G181300	GCTCGCACCTATGATCTCGC	CCTATCCTCGCCTCCCACC	CGGCGTCTGTTTCGCTTCC	CGCTACCGCAGCCTATCC		
**C16**	TraesCS4A01G216900	CGGTGCCGTCGTCCAAGAT	ACCACCACCCGAGTGCGATAT				
**C17**	TraesCS4A01G217000	GGCGTCCTCGGGGCTCTTT	GCGGTCATGTCGTCGTCCA				
**C18**	TraesCS4A01G255700	CTCCTCACGCTCTTTCCCA	TAGATGCCGCCCCTGAAG				
**C19**	TraesCS4A01G255800	TGCCAGAGCAAGAAGGAATC	AGGGTGTCTCGGACGCTTA				

Supplementary Table S2. Candidate genes and markers linked to resistance genes *YrM86664-3* on chromosome 4AL.

Position (bp)	No. of selected genes / Markers of *YrM8664-3*	Gene accession	Annotation
415683496-415683891	*BE446584*		
456290064-456292784	C15	TraesCS4A01G181300	AP2-like ethylene-responsive transcription factor
456292321-456292391	*AX111655681*		
456293850-456294616		TraesCS4A01G181400	Calcium-binding protein
456336315-456336770		TraesCS4A01G181500	Calcium-binding protein
456436432-456437241		TraesCS4A01G181600	Germin-like protein
456449294-456449968		TraesCS4A01G181700	Germin-like protein 1-1
456454952-456456006		TraesCS4A01G181800	Germin-like protein
456459540-456460466		TraesCS4A01G181900	Germin-like protein 1-1
456479864-456482398		TraesCS4A01G182000	transmembrane protein, putative (Protein of unknown function, DUF538)
456483993-456489034		TraesCS4A01G182100	tRNA (Ile)-lysidine synthase
457568303-457568758		TraesCS4A01G182200	Calcium-binding protein
457826318-457836526		TraesCS4A01G182300	Clathrin heavy chain
458233726-458261311		TraesCS4A01G182400	Proline-, glutamic acid-and leucine-rich protein 1

458517792-458534396	TraesCS4A01G182500	Proline-, glutamic acid-and leucine-rich protein 1
458856406-458858932	TraesCS4A01G182600	60S ribosomal protein L44
459627628-459631283	TraesCS4A01G182700	Omega-3 fatty acid desaturase
459631134-459637226	TraesCS4A01G182800	Chaperone ClpB
460229896-460238212	TraesCS4A01G182900	E3 SUMO-protein ligase SIZ1
460238966-460240171	TraesCS4A01G183000	Kinase family protein
460448898-460449233	TraesCS4A01G183100	Protein transport protein Sec61 subunit beta, putative
460451001-460454968	TraesCS4A01G183200	RNA-directed DNA methylation 4
460459288-460460764	TraesCS4A01G183300	Alpha/beta-Hydrolases superfamily protein, putative
460897370-460902867	TraesCS4A01G183400	transcription factor-like protein
460903866-460904732	TraesCS4A01G183500	Membrane-associated phospholipid phosphatase
461353961-461355490	TraesCS4A01G183600	Cytochrome P450
462201615-462203147	TraesCS4A01G183700	RING/U-box superfamily protein
462202745-462205391	TraesCS4A01G183800	caspase-6 protein
462204109-462205056	TraesCS4A01G183900	DUF868 family protein
462210624-462211955	TraesCS4A01G184000	Seipin
462212695-462216068	TraesCS4A01G184100	2,3-bisphosphoglycerate-dependent phosphoglycerate mutase

462216501-462219266	TraesCS4A01G184200	ATP-dependent RNA helicase
462219982-462224642	TraesCS4A01G184300	myosin-4 protein (DUF641)
462309235-462309875	TraesCS4A01G184400	SKP1-interacting partner 15
462825330-462834056	TraesCS4A01G184500	Nucleosome-remodeling factor subunit BPTF
463543053-463543944	TraesCS4A01G184600	stress response NST1-like protein
463923382-463925340	TraesCS4A01G184700	Heavy metal transport/detoxification superfamily protein
464110898-464112499	TraesCS4A01G184800	R3H domain protein
464549148-464568089	TraesCS4A01G184900	Long-Chain Acyl-CoA Synthetase
464685913-464691515	TraesCS4A01G185000	Protein Red
464692585-464694075	TraesCS4A01G185100	Structure-specific endonuclease subunit slx1
464696741-464696962	TraesCS4A01G185200	Ubiquitin-like protein 5
464762884-464764368	TraesCS4A01G185300	Glycosyltransferase
464767703-464771611	TraesCS4A01G185400	DNA-directed RNA polymerase II, putative
464989058-464989620	TraesCS4A01G185500	RING/U-box superfamily protein, putative
464989922-464994481	TraesCS4A01G185600	Phosphatidylinositol-4-phosphate 5-kinase 4, putative
465006134-465006929	TraesCS4A01G185700	RING/U-box superfamily protein, putative
465223300-465223743	TraesCS4A01G185800	RING/U-box superfamily protein

465226193-465228911	TraesCS4A01G185900	Scarecrow-like transcription factor 3 family protein
465275101-465276978	TraesCS4A01G186000	Pentatricopeptide repeat-containing family protein
465326992-465330134	TraesCS4A01G186100	Calcium-binding EF-hand protein
465403704-465408630	TraesCS4A01G186200	RNA-binding protein
465408869-465411396	TraesCS4A01G186300	50S ribosomal protein L24
465523379-465524129	TraesCS4A01G186400	Invertase/pectin methylesterase inhibitor family protein
465526094-465533623	TraesCS4A01G186500	Alpha-L-arabinofuranosidase 1
465535456-465536736	TraesCS4A01G186600	Ubiquitin carboxyl-terminal hydrolase family protein
465542897-465545035	TraesCS4A01G186700	Serine/threonine-protein kinase
465790367-465790657	TraesCS4A01G186800	Phytosulfokines 3
465794366-465799828	TraesCS4A01G186900	Heme oxygenase 1 protein
465890222-465892744	TraesCS4A01G187000	Serine/threonine-protein kinase
465893542-465899342	TraesCS4A01G187100	Glutamine--fructose-6-phosphate aminotransferase [isomerizing]
466107721-466111852	TraesCS4A01G187200	Rhamnogalacturonate lyase
466571843-466575387	TraesCS4A01G187300	Non-specific serine/threonine protein kinase
466632749-466636729	TraesCS4A01G187400	WD-repeat protein, putative
466638350-466644033	TraesCS4A01G187500	Protein ZINC INDUCED FACILITATOR-LIKE 1

466648899-466656351	TraesCS4A01G187600	Protein ZINC INDUCED FACILITATOR-LIKE 1
466910566-466916544	TraesCS4A01G187700	Protein ZINC INDUCED FACILITATOR-LIKE 1
466922580-466924670	TraesCS4A01G187800	4'-phosphopantetheinyl transferase family protein
466925867-466928007	TraesCS4A01G187900	Calcium-dependent protein kinase
466929045-466934390	TraesCS4A01G188000	S-locus lectin protein kinase family protein
466938805-466941669	TraesCS4A01G188100	Auxin efflux carrier component
467142674-467145702	TraesCS4A01G188200	weak chloroplast movement under blue light protein (DUF827)
467245403-467251748	TraesCS4A01G188300	Alpha-L-arabinofuranosidase 1
467258814-467260581	TraesCS4A01G188400	Mitochondrial import inner membrane translocase subunit
467264938-467266728	TraesCS4A01G188500	Pentatricopeptide repeat-containing protein
467311125-467312751	TraesCS4A01G188600	Lactation elevated protein 1
467516912-467538652	TraesCS4A01G188700	Regulation of nuclear pre-mRNA domain-containing protein 1B
467539358-467539961	TraesCS4A01G188800	Nuclear-interacting partner of ALK
467543882-467547234	TraesCS4A01G188900	ELMO domain containing protein
467681716-467686145	TraesCS4A01G189000	NAD(P)-binding Rossmann-fold superfamily protein
468147360-468160559	TraesCS4A01G189100	Methyl esterase
468207784-468209254	TraesCS4A01G189200	50S ribosomal protein L2

468210429-468210704	TraesCS4A01G189300	B3 domain-containing protein
468214233-468214691	TraesCS4A01G189400	Serine/threonine-protein phosphatase 4 regulatory subunit 3
468247494-468247931	TraesCS4A01G189500	Retrotransposon protein, putative, unclassified
468248620-468251401	TraesCS4A01G189600	Type I inositol-1,4,5-trisphosphate 5-phosphatase
468603186-468604307	TraesCS4A01G189700	Myb factor
468624255-468625997	TraesCS4A01G189800	Transposon Ty3-G Gag-Pol polyprotein
468627132-468628964	TraesCS4A01G189900	Cell division control protein 45-like protein
468652211-468652723	TraesCS4A01G190000	Ubiquitin-like-specific protease ESD4-like protein
468752522-468754917	TraesCS4A01G190100	Exostosin family protein
468757496-468758122	TraesCS4A01G190200	30S ribosomal protein S9
468849301-468849665	TraesCS4A01G190300	Ribonuclease H-like superfamily protein
469101407-469106508	TraesCS4A01G190400	Zeaxanthin epoxidase, chloroplastic
469101882-469102650	TraesCS4A01G190500	30S ribosomal protein S9
469240692-469243251	TraesCS4A01G190600	2-C-methyl-D-erythritol 4-phosphate cytidylyltransferase
469244809-469246370	TraesCS4A01G190700	Dirigent protein
469512189-469515279	TraesCS4A01G190800	RNA-binding domain CCCH-type zinc finger protein
469829765-469839951	TraesCS4A01G190900	33 kDa chaperonin

470097916-470100678	TraesCS4A01G191000	Pentatricopeptide repeat-containing protein
470136300-470138148	TraesCS4A01G191100	Calcium uniporter protein, mitochondrial
470568648-470572941	TraesCS4A01G191200	NAD(P)-binding Rossmann-fold superfamily protein
470687270-470689755	TraesCS4A01G191300	Scarecrow transcription factor family protein
471695861-471712108	TraesCS4A01G191400	Ethanolamine-phosphate cytidylyltransferase
471917815-471921872	TraesCS4A01G191500	Kinase family protein
471929454-471929858	TraesCS4A01G191600	P-loop containing nucleoside triphosphate hydrolases superfamily protein
471930743-471933286	TraesCS4A01G191700	Bis(5'-adenosyl)-triphosphatase
471937946-471938830	TraesCS4A01G191800	Retrotransposon protein, putative, unclassified
472194252-472198000	TraesCS4A01G191900	S-acyltransferase
472398571-472399305	TraesCS4A01G192000	LURP-one-like protein
472613393-472616378	TraesCS4A01G192100	DUF936 family protein
472648853-472651264	TraesCS4A01G192200	GH3 family protein
472918003-472921049	TraesCS4A01G192300	Protein kinase
472922679-472923350	TraesCS4A01G192400	Late embryogenesis abundant hydroxyproline-rich glycoprotein family, putative
472963183-472965902	TraesCS4A01G192500	Cytochrome P450-like protein

473496793-473508086	TraesCS4A01G192600	Coiled-coil domain-containing protein SCD2
473686557-473689766	TraesCS4A01G192700	heat-inducible transcription repressor
473765103-473767432	TraesCS4A01G192800	DUF1191 superfamily protein
473940082-473942130	TraesCS4A01G192900	Phototropic-responsive NPH3 family protein
473945535-473946623	TraesCS4A01G193000	BTB/POZ domain containing protein, expressed
474494136-474495065	TraesCS4A01G193100	Ethylene-responsive transcription factor, putative
474740916-474742519	TraesCS4A01G193200	Methyl esterase 17
474742975-474744402	TraesCS4A01G193300	Methyl esterase 17
474932400-474935597	TraesCS4A01G193400	Wall-associated receptor kinase 5
475389649-475390570	TraesCS4A01G193500	Polynucleotidyl transferase, ribonuclease H-like superfamily protein
475396081-475398440	TraesCS4A01G193600	WRKY transcription factor
475418550-475423746	TraesCS4A01G193700	GPI transamidase component PIG-T
475579120-475579921	TraesCS4A01G193800	AMP phosphorylase
475902731-475906968	TraesCS4A01G193900	Vacuolar sorting receptor family protein
475910551-475914614	TraesCS4A01G194000	Chloroplastic outer envelope membrane protein, putative, expressed
475914952-475919117	TraesCS4A01G194100	Elongation factor P
476065837-476066397	TraesCS4A01G194200	Ring finger protein, putative

476142148-476142510	TraesCS4A01G194300	Non-specific lipid-transfer protein
476145519-476146036	TraesCS4A01G194400	Non-specific lipid-transfer protein
476337610-476343287	TraesCS4A01G194500	MACPF domain protein
476345073-476346656	TraesCS4A01G194600	Pentatricopeptide repeat-containing protein
476744781-476752651	TraesCS4A01G194700	Poly (A) RNA polymerase cid14
476979044-476980393	TraesCS4A01G194800	Non-specific serine/threonine protein kinase
477019716-477022706	TraesCS4A01G194900	NBS-LRR resistance protein
477024828-477029268	TraesCS4A01G195000	ARM repeat superfamily protein
477026271-477026606	TraesCS4A01G195100	Class II aaRS and biotin synthetases superfamily protein
477032809-477034972	TraesCS4A01G195200	Methyltransferase-related protein, putative
477057713-477066814	TraesCS4A01G195300	Reticulocyte-binding protein 2 like a
477307630-477313109	TraesCS4A01G195400	Reticulocyte-binding protein 2 like a
477833503-477834478	TraesCS4A01G195500	Lipid transfer protein
477878202-477884489	TraesCS4A01G195600	ADP,ATP carrier protein
477886282-477888721	TraesCS4A01G195700	Saposin B domain-containing family protein
478191459-478192551	TraesCS4A01G195800	Ribosomal RNA apurinic site specific lyase-like
478209590-478216829	TraesCS4A01G195900	F-box family protein

478217881-478219705	TraesCS4A01G196000	Peroxidase
478227516-478228331	TraesCS4A01G196100	DNA topoisomerase
478228425-478228727	TraesCS4A01G196200	Cortactin-binding protein 2
478300560-478302737	TraesCS4A01G196300	Peroxidase
478338363-478340040	TraesCS4A01G196400	Peroxidase
478742792-478743707	TraesCS4A01G196500	type one serine/threonine protein phosphatase 6
478746523-478747467	TraesCS4A01G196600	Carbonic anhydrase
478751090-478752731	TraesCS4A01G196700	F-box family protein
478866899-478868300	TraesCS4A01G196800	BTB/POZ domain-containing protein
479400478-479402257	TraesCS4A01G196900	Serine/threonine-protein kinase Haspin, putative
479411679-479419052	TraesCS4A01G197000	XH/XS domain-containing family protein
479429746-479430999	TraesCS4A01G197100	F-box family protein
479623255-479625385	TraesCS4A01G197200	F-box family protein
480556438-480557244	TraesCS4A01G197300	F-box family protein
481063475-481064905	TraesCS4A01G197400	F-box family protein
481089393-481091981	TraesCS4A01G197500	Transposase
481409030-481415020	TraesCS4A01G197600	Signal recognition particle 54 kDa protein

481416936-481420804	TraesCS4A01G197700	Signal recognition particle 54 kDa protein
482132676-482133512	TraesCS4A01G197800	Fantastic four-like protein
482134696-482138841	TraesCS4A01G197900	Rho GTPase-activating protein
482391745-482392520	TraesCS4A01G198000	Cholesterol 7-alpha-monooxygenase
483174530-483177374	TraesCS4A01G198100	Transcription factor
483382511-483383497	TraesCS4A01G198200	F-box protein-like
483950328-483951403	TraesCS4A01G198300	B3 domain-containing protein
484097808-484099293	TraesCS4A01G198400	Alpha-1,4 glucan phosphorylase
484234292-484236962	TraesCS4A01G198500	Lysosomal Pro-X carboxypeptidase
484672134-484673187	TraesCS4A01G198600	transcription repressor
484731624-484732719	TraesCS4A01G198700	transcription repressor
485278938-485284016	TraesCS4A01G198800	HVA22-like protein
485284330-485285110	TraesCS4A01G198900	ATP binding microtubule motor family protein
485458851-485461751	TraesCS4A01G199000	Chaperone protein ClpB
485708491-485709243	TraesCS4A01G199100	Basic blue copper family protein
486124572-486126014	TraesCS4A01G199200	UDP-glucose 6-dehydrogenase
486638806-486643365	TraesCS4A01G199300	Cyclin family protein

486646174-486648440	TraesCS4A01G199400	Pentatricopeptide repeat-containing protein
487554653-487555210	TraesCS4A01G199500	Phospholipid-transporting ATPase
487862149-487866232	TraesCS4A01G199600	Cyclin family protein
487868350-487868991	TraesCS4A01G199700	Late embryogenesis abundant (LEA) hydroxyproline-rich glycoprotein family
487890910-487892157	TraesCS4A01G199800	Late embryogenesis abundant (LEA) hydroxyproline-rich glycoprotein family, putative
487916374-487918245	TraesCS4A01G199900	FBD-associated F-box protein
488110321-488112219	TraesCS4A01G200000	Non-specific serine/threonine protein kinase
488252084-488257189	TraesCS4A01G200100	Exocyst complex component, putative
488528980-488534766	TraesCS4A01G200200	Two-component response regulator-like protein
488535721-488536287	TraesCS4A01G200300	5'-AMP-activated protein kinase beta-2 subunit protein
489083272-489084464	TraesCS4A01G200400	Apoptosis-inducing factor
489090971-489091890	TraesCS4A01G200500	Pentatricopeptide repeat-containing protein family
489096748-489109008	TraesCS4A01G200600	Digalactosyldiacylglycerol synthase 1, chloroplastic
489426392-489431170	TraesCS4A01G200700	Transcription factor-related family protein
489432513-489436674	TraesCS4A01G200800	BEL1-like homeodomain protein 1
489434254-489434619	TraesCS4A01G200900	cysteine-rich RLK (RECEPTOR-like protein kinase) 40

489882900-489889416	TraesCS4A01G201000	Phox-associated domain,Phox-like,Sorting nexin isoform 3
490341785-490342748	TraesCS4A01G201100	B3 domain-containing protein
490356118-490360729	TraesCS4A01G201200	GDP-L-galactose phosphorylase 1
490593952-490604685	TraesCS4A01G201300	Phosphatidylinositol 4-kinase
490770671-490776196	TraesCS4A01G201400	Cysteine desulfurase
490776678-490776968	TraesCS4A01G201500	Retrotransposon protein, putative, unclassified
490919189-490920803	TraesCS4A01G201600	E3 ubiquitin-protein ligase
491452647-491453923	TraesCS4A01G201700	Peroxidase
491687825-491690070	TraesCS4A01G201800	Receptor lectin kinase
491709686-491710078	TraesCS4A01G201900	Fusarium resistance orphan protein
491712891-491713484	TraesCS4A01G202000	Expressed protein-RZ53
491715851-491719316	TraesCS4A01G202100	Alcohol dehydrogenase
491914988-491917720	TraesCS4A01G202200	Alcohol dehydrogenase
492030110-492032620	TraesCS4A01G202300	Alcohol dehydrogenase
492352265-492355446	TraesCS4A01G202400	HXXXD-type acyl-transferase family protein
492527492-492528949	TraesCS4A01G202500	Pentatricopeptide repeat-containing protein
492531159-492534557	TraesCS4A01G202600	Carboxypeptidase

492888236-492891745	TraesCS4A01G202700	Transport Sec24 family protein
492893652-492893990	TraesCS4A01G202800	Polynucleotidyl transferase, ribonuclease H-like superfamily protein
492938661-492943177	TraesCS4A01G202900	Sentrin-specific protease
493614079-493614654	TraesCS4A01G203000	Dirigent protein
493982436-493983254	TraesCS4A01G203100	Ta11-like non-LTR retrotransposon
494001389-494001703	TraesCS4A01G203200	cytochrome P450, family 705, subfamily A, polypeptide 1
494002781-494009702	TraesCS4A01G203300	Pyruvate kinase
494009956-494010940	TraesCS4A01G203400	Chaperone protein dnaJ
494044874-494045656	TraesCS4A01G203500	Tapetum determinant 1
494144197-494144595	TraesCS4A01G203600	Protein Ycf2 A
494457303-494457896	TraesCS4A01G203700	Protein tapetum determinant 1
494552792-494558815	TraesCS4A01G203800	ABC1-like kinase
494640795-494643747	TraesCS4A01G203900	DAG protein, chloroplastic
495078320-495087074	TraesCS4A01G204000	Methylthioribose-1-phosphate isomerase
495101081-495124433	TraesCS4A01G204100	mRNA-capping enzyme
495106845-495107264	TraesCS4A01G204200	hAT transposon superfamily
495796296-495799843	TraesCS4A01G204300	Basic-leucine zipper (bZIP) transcription factor family protein

495801483-495803606	TraesCS4A01G204400	DNA-directed RNA polymerase
496062509-496074870	TraesCS4A01G204500	ATP-dependent protease La (LON) domain-containing protein
496113929-496115045	TraesCS4A01G204600	Interferon-induced protein 44
496997901-497003812	TraesCS4A01G204700	ATP-dependent zinc metalloprotease FtsH
497010610-497013169	TraesCS4A01G204800	Ribosomal protein
497024216-497029204	TraesCS4A01G204900	Auxin-responsive protein
497075203-497076970	TraesCS4A01G205000	Auxin-responsive protein
497439926-497442580	TraesCS4A01G205100	Kinase family protein
497494359-497496234	TraesCS4A01G205200	Serpin family protein
497810191-497812673	TraesCS4A01G205300	RNA apurinic site specific lyase
497814133-497815577	TraesCS4A01G205400	XH/XS domain protein
497871084-497872221	TraesCS4A01G205500	ERD (early-responsive to dehydration stress) family protein
497888723-497890741	TraesCS4A01G205600	Protein FRIGIDA
498212114-498213790	TraesCS4A01G205700	glucan synthase-like 10
498675787-498677685	TraesCS4A01G205800	Receptor-like kinase
498693152-498701803	TraesCS4A01G205900	Shortage in chiasmata
498985630-498988773	TraesCS4A01G206000	Receptor-kinase, putative

498993712-498999838	TraesCS4A01G206100	FHA domain containing protein, expressed
499341534-499344935	TraesCS4A01G206200	protein kinase family protein
499351019-499352479	TraesCS4A01G206300	Integrator complex subunit 3-like protein
499354369-499356918	TraesCS4A01G206400	Fructose-bisphosphate aldolase
499953247-499954317	TraesCS4A01G206500	AGC (cAMP-dependent, cGMP-dependent and protein kinase C) kinase family protein
500113292-500118392	TraesCS4A01G206600	CBS domain-containing protein, putative, expressed
500139035-500144802	TraesCS4A01G206700	Filament-like plant protein
500146447-500151023	TraesCS4A01G206800	Glutamyl-tRNA(Gln) amidotransferase subunit A
500349681-500351705	TraesCS4A01G206900	V-type proton ATPase proteolipid subunit
500352424-500357005	TraesCS4A01G207000	Long-Chain Acyl-CoA Synthetase
500412502-500422120	TraesCS4A01G207100	Protein FAR1-RELATED SEQUENCE 5
500431374-500433104	TraesCS4A01G207200	Glyoxal oxidase
500629797-500631119	TraesCS4A01G207300	Hyccin
500635505-500637009	TraesCS4A01G207400	transmembrane protein, putative (DUF247)
500658557-500660041	TraesCS4A01G207500	C2H2-like zinc finger protein
500900825-500902375	TraesCS4A01G207600	Auxin influx transporter

500905280-500912000	TraesCS4A01G207700	Sec1 family domain-containing protein 2
501123018-501125005	TraesCS4A01G207800	Receptor-like kinase
501125644-501129860	TraesCS4A01G207900	Alba DNA/RNA-binding protein
501132294-501134463	TraesCS4A01G208000	Ribosomal protein L3
501138148-501140321	TraesCS4A01G208100	Syntaxin protein
501243616-501244080	TraesCS4A01G208200	Egg cell-secreted protein 1.1
501260011-501260936	TraesCS4A01G208300	Ta11-like non-LTR retrotransposon
501468566-501469147	TraesCS4A01G208400	Abscisic stress-ripening protein
501638027-501640621	TraesCS4A01G208500	Exocyst complex component, putative
501959071-501961411	TraesCS4A01G208600	heat shock protein
501961857-501966950	TraesCS4A01G208700	Meiosis arrest female protein 1
501967261-501972388	TraesCS4A01G208800	Protein FAM135B
501975190-501976419	TraesCS4A01G208900	Plant/F1M20-13 protein
502027925-502030925	TraesCS4A01G209000	Tubby-like F-box protein
502356611-502357930	TraesCS4A01G209100	Transcription factor, putative
502362586-502364491	TraesCS4A01G209200	Transposon protein, putative, Pong sub-class
502900389-502901207	TraesCS4A01G209300	MAU2 chromatid cohesion factor homolog

502901041-502905693	TraesCS4A01G209400	Cytochrome b-c1 complex subunit 6
503212518-503219328	TraesCS4A01G209500	Autophagy-related protein 13
503235890-503236489	TraesCS4A01G209600	Vacuolar iron transporter-like protein
503611371-503612855	TraesCS4A01G209700	NBS-LRR-like resistance protein
503616427-503617851	TraesCS4A01G209800	GRAS family transcription factor containing protein
503629170-503632543	TraesCS4A01G209900	Transcription factor
503683127-503683996	TraesCS4A01G210000	Major cell-surface adhesin PAc
504172557-504173584	TraesCS4A01G210100	Heavy metal transport/detoxification superfamily protein
504183854-504198776	TraesCS4A01G210200	RING/FYVE/PHD zinc finger protein, putative
504199319-504200044	TraesCS4A01G210300	histone acetyltransferase of the CBP family 12
504201715-504202428	TraesCS4A01G210400	DUF241 domain protein, putative (DUF241)
504204317-504205117	TraesCS4A01G210500	DUF241 domain protein, putative (DUF241)
504207555-504208259	TraesCS4A01G210600	DUF241 domain protein, putative (DUF241)
504210903-504211694	TraesCS4A01G210700	DUF241 domain protein, putative (DUF241)
504214309-504214932	TraesCS4A01G210800	DUF241 domain protein (DUF241)
504216896-504217294	TraesCS4A01G210900	DUF241 domain protein, putative (DUF241)
504675538-504676842	TraesCS4A01G211000	ORMDL family protein-like

504789781-504792520	TraesCS4A01G211100	MYB transcription factor
504860925-504875568	TraesCS4A01G211200	Polyamine oxidase-like protein
504875850-504876248	TraesCS4A01G211300	Small conductance calcium-activated potassium channel protein 2
505495653-505496209	TraesCS4A01G211400	Heavy-metal-associated domain-containing protein, putative, expressed
505789086-505790276	TraesCS4A01G211500	exosome complex exonuclease
505790616-505794673	TraesCS4A01G211600	Kinase
506164630-506171334	TraesCS4A01G211700	Transcriptional corepressor SEUSS
506709256-506720722	TraesCS4A01G211800	Transcriptional corepressor SEUSS
507107850-507108270	TraesCS4A01G211900	GTPase Der
507561295-507561837	TraesCS4A01G212000	Coiled-coil domain-containing protein 18, putative isoform 1
507592560-507594188	TraesCS4A01G212100	FAD-binding Berberine family protein, putative
507908870-507910036	TraesCS4A01G212200	External alternative NAD(P)H-ubiquinone oxidoreductase B2, mitochondrial
507959947-507962049	TraesCS4A01G212300	F-box family protein
507964089-507975633	TraesCS4A01G212400	Cysteine synthase
508273954-508274503	TraesCS4A01G212500	Acetyltransferase, GNAT family protein, expressed
508494103-508494603	TraesCS4A01G212600	Acetyltransferase (GNAT) domain protein
510638985-510639524	TraesCS4A01G212700	N-acetyllactosaminide alpha-2,3-sialyltransferase (Sialyltransferase 6)

511022104-511024508	TraesCS4A01G212800	UDP-N-acetylglucosamine--N-acetylmuramyl-(pentapeptide) pyrophosphoryl-undecaprenol N-acetylglucosamine transferase
511036932-511038507	TraesCS4A01G212900	Pentatricopeptide repeat-containing protein, putative
511079422-511083784	TraesCS4A01G213000	F-box family protein
511132296-511133523	TraesCS4A01G213100	NAC domain protein
511187957-511189477	TraesCS4A01G213200	Histone deacetylase
511227620-511229247	TraesCS4A01G213300	Monooxygenase, FAD-binding protein
511230595-511232468	TraesCS4A01G213400	Nucleoside diphosphate kinase
511702496-511703827	TraesCS4A01G213500	Aspartic proteinase nepenthesin-1
511753946-511754482	TraesCS4A01G213600	Eukaryotic translation initiation factor 3 subunit A
511824039-511826026	TraesCS4A01G213700	Dynamin family protein, putative, expressed
512012996-512023533	TraesCS4A01G213800	Uridine kinase
512020435-512021019	TraesCS4A01G213900	aluminum-activated malate transporter 1
512024465-512028065	TraesCS4A01G214000	myosin XI D
512343253-512349340	TraesCS4A01G214100	TOM1-like protein 2
512354009-512357372	TraesCS4A01G214200	Protein disulfide-isomerase
512538021-512539064	TraesCS4A01G214300	Protein BPS1, chloroplastic

512539933-512545282	TraesCS4A01G214400	transmembrane protein (DUF616)
512579358-512581370	TraesCS4A01G214500	DnaJ domain containing protein, expressed
512835458-512836424	TraesCS4A01G214600	Glycosyl hydrolase superfamily protein
513082798-513087480	TraesCS4A01G214700	B3 domain-containing protein
513089416-513089859	TraesCS4A01G214800	Ribonuclease E inhibitor RraA/Dimethylmenaquinone methyltransferase
513120167-513121106	TraesCS4A01G214900	Retrotransposon protein, putative, unclassified
513288348-513292999	TraesCS4A01G215000	magnesium transporter NIPA (DUF803)
513341536-513342366	TraesCS4A01G215100	WEB family protein
513349444-513349725	TraesCS4A01G215200	ABC-2 and Plant PDR ABC-type transporter family protein
513804403-513805973	TraesCS4A01G215300	Amino acid permease
513922366-513922801	TraesCS4A01G215400	Neurogenic differentiation factor 1
513999399-514003377	TraesCS4A01G215500	Rubredoxin
514078413-514081489	TraesCS4A01G215600	50S ribosomal protein L19, putative
514341519-514344118	TraesCS4A01G215700	F-box family protein
514353267-514354960	TraesCS4A01G215800	F-box family protein
514406360-514406623	TraesCS4A01G215900	Pyridoxine/pyridoxamine 5'-phosphate oxidase
514412272-514421374	TraesCS4A01G216000	Cytochrome P450

514429586-514433273		TraesCS4A01G216100	Phytoalexin-deficient 4-1 protein
514556905-514569017		TraesCS4A01G216200	Protein translocase subunit SecA
515000857-515007042		TraesCS4A01G216300	ATP-dependent RNA helicase family protein
515456321-515457192		TraesCS4A01G216400	Holliday junction ATP-dependent DNA helicase RuvA
515461630-515466775		TraesCS4A01G216500	O-fucosyltransferase family protein
515558080-515558421		TraesCS4A01G216600	phospholipase-like protein (PEARLI 4) family protein
515646346-515649454		TraesCS4A01G216700	Cyclic nucleotide-gated channel
515749585-515750250		TraesCS4A01G216800	Late embryogenesis abundant (LEA) hydroxyproline-rich glycoprotein
515779130-515782750	C16	TraesCS4A01G216900	ABC transporter G family member
516343042-516343722	C17	TraesCS4A01G217000	Late embryogenesis abundant (LEA) hydroxyproline-rich glycoprotein
516354856-516355404		TraesCS4A01G217100	Dirigent protein
516369536-516370081		TraesCS4A01G217200	Dirigent protein
516583296-516583874		TraesCS4A01G217300	Dirigent protein
516587843-516608751		TraesCS4A01G217400	Dirigent protein
516890842-516894346		TraesCS4A01G217500	DUF668 family protein
516931760-516937462		TraesCS4A01G217600	Receptor-type tyrosine-protein phosphatase
517400440-517410772		TraesCS4A01G217700	ATP-dependent RNA helicase RhlB

517884383-517885453	TraesCS4A01G217800	MYB transcription factor
518822694-518829375	TraesCS4A01G217900	Transmembrane 9 superfamily member
518970702-518985627	TraesCS4A01G218000	Protein ZINC INDUCED FACILITATOR-LIKE 1
519570632-519574956	TraesCS4A01G218100	MLO-like protein
519630531-519633743	TraesCS4A01G218200	Fe/S biogenesis protein nfuA
519675892-519678306	TraesCS4A01G218300	Short-chain dehydrogenase/reductase family protein
520125235-520127884	TraesCS4A01G218400	Methylsterol monooxygenase 1-2
520954343-520955011	TraesCS4A01G218500	arginine N-methyltransferase, putative (DUF688)
521069473-521078564	TraesCS4A01G218600	WD40 repeat-like protein
521263892-521272090	TraesCS4A01G218700	SWI/SNF complex subunit SWI3C
521300093-521300491	TraesCS4A01G218800	RNA-directed DNA polymerase; Ribonuclease H, related
521538536-521551738	TraesCS4A01G218900	U-box domain-containing protein
521556213-521557786	TraesCS4A01G219000	Replication protein A subunit
521557979-521562568	TraesCS4A01G219100	E3 ubiquitin-protein ligase SINA-like 10
521588243-521589369	TraesCS4A01G219200	Purine nucleoside phosphorylase DeoD-type
521602640-521603779	TraesCS4A01G219300	P-loop containing nucleoside triphosphate hydrolases superfamily protein
521624277-521625872	TraesCS4A01G219400	Heavy metal-associated protein

521812354-521815231	TraesCS4A01G219500	Aspartic proteinase Asp1
521846077-521847275	TraesCS4A01G219600	Eukaryotic aspartyl protease family protein, expressed
521918428-521919907	TraesCS4A01G219700	NAC domain-containing protein, putative
521945567-521946917	TraesCS4A01G219800	Major Facilitator Superfamily with SPX (SYG1/Pho81/XPR1) domain-containing protein
522153951-522157524	TraesCS4A01G219900	Aldehyde dehydrogenase
522293017-522295793	TraesCS4A01G220000	S-adenosyl-L-methionine-dependent methyltransferases superfamily protein
522907855-522921868	TraesCS4A01G220100	DNA polymerase
522972703-522973478	TraesCS4A01G220200	Pre-mRNA splicing factor, putative
523447064-523455580	TraesCS4A01G220300	Kinase family protein
523456273-523458075	TraesCS4A01G220400	Proteasome subunit beta type
524178707-524179452	TraesCS4A01G220500	Pre-mRNA splicing factor, putative
524272281-524273502	TraesCS4A01G220600	DNA ligase
524280168-524287073	TraesCS4A01G220700	Chromatin modification-related protein EAF1 A
525043352-525044383	TraesCS4A01G220800	Transposase
525110484-525111035	TraesCS4A01G220900	RING/U-box superfamily protein
525152818-525153189	TraesCS4A01G221000	Cartilage oligomeric matrix protein

525736772-525737806	TraesCS4A01G221100	GATA transcription factor
526640028-526642987	TraesCS4A01G221200	WD-repeat protein, putative
527001528-527002834	TraesCS4A01G221300	1-aminocyclopropane-1-carboxylate oxidase
527009136-527014811	TraesCS4A01G221400	NAD kinase
527112680-527113582	TraesCS4A01G221500	Sphingoid base hydroxylase 2
527120170-527132709	TraesCS4A01G221600	DNA ligase 1
527495980-527500050	TraesCS4A01G221700	Protein kinase family protein, putative, expressed
527575814-527576790	TraesCS4A01G221800	Myb/SANT-like DNA-binding domain protein
527902974-527913262	TraesCS4A01G221900	Transducin/WD-like repeat-protein
528604540-528605440	TraesCS4A01G222000	F-box family protein
528644556-528645197	TraesCS4A01G222100	F-box domain containing protein
529619251-529626266	TraesCS4A01G222200	Protein TPLATE
529625774-529626034	TraesCS4A01G222300	Homoserine/homoserine lactone efflux protein
529626929-529628377	TraesCS4A01G222400	Pentatricopeptide repeat-containing protein, putative
529629746-529631972	TraesCS4A01G222500	B3 domain-containing protein
529633638-529634432	TraesCS4A01G222600	Serpin family protein
530121870-530123027	TraesCS4A01G222700	Transcription factor, putative

530172977-530179549	TraesCS4A01G222800	RING-finger E3 ubiquitin ligase, putative
530815792-530819262	TraesCS4A01G222900	Neutral/alkaline invertase
530891468-530892193	TraesCS4A01G223000	Beta-1,3-N-Acetylglucosaminyltransferase family protein, putative
530935273-530935944	TraesCS4A01G223100	Harpin inducing protein
530936889-530938446	TraesCS4A01G223200	receptor kinase 1
531073743-531077003	TraesCS4A01G223300	NBS-LRR disease resistance protein-like
531077535-531097706	TraesCS4A01G223400	protein kinase family protein
531236819-531241694	TraesCS4A01G223500	Disease resistance protein RPM1
531247082-531249319	TraesCS4A01G223600	receptor kinase 1
531304423-531309137	TraesCS4A01G223700	Photosystem II reaction center PsbP family protein
531311802-531313877	TraesCS4A01G223800	Receptor-like protein kinase
531325926-531326657	TraesCS4A01G223900	receptor kinase 1
531383913-531386654	TraesCS4A01G224000	NBS-LRR disease resistance protein, putative, expressed
531677814-531680552	TraesCS4A01G224100	NBS-LRR disease resistance protein, putative, expressed
531952996-531955530	TraesCS4A01G224200	Transposon protein, putative, CACTA, En/Spm sub-class
532073805-532075311	TraesCS4A01G224300	Rpr4901.2
532208397-532209491	TraesCS4A01G224400	receptor kinase 1

532216853-532217890	TraesCS4A01G224500	NBS-LRR disease resistance protein, putative, expressed
532223673-532224099	TraesCS4A01G224600	Pentatricopeptide repeat (PPR) superfamily protein
532350376-532353361	TraesCS4A01G224700	Receptor protein kinase, putative
532791942-532797429	TraesCS4A01G224800	Non-specific serine/threonine protein kinase
532833164-532834197	TraesCS4A01G224900	Gibberellin receptor GID1a
532897177-532898552	TraesCS4A01G225000	Gibberellin receptor GID1A
533000510-533006669	TraesCS4A01G225100	Sucrose-phosphate synthase
533010789-533015786	TraesCS4A01G225200	Glycerophosphodiester phosphodiesterase
533032338-533032802	TraesCS4A01G225300	VQ motif-containing protein
533258157-533262230	TraesCS4A01G225400	Protein NRT1/ PTR FAMILY 1.2
533444658-533448029	TraesCS4A01G225500	PHD finger protein
533459478-533463467	TraesCS4A01G225600	B3 domain-containing protein
533843378-533844403	TraesCS4A01G225700	Gibberellin receptor GID1a
534042906-534057343	TraesCS4A01G225800	CCR4-NOT transcription complex subunit 11
534160530-534161546	TraesCS4A01G225900	BPS1-like protein (DUF793)
534167672-534170275	TraesCS4A01G226000	ATP-dependent Clp protease ATP-binding subunit
534229856-534237751	TraesCS4A01G226100	Serine carboxypeptidase family protein, expressed

534258202-534258657	TraesCS4A01G226200	DUF538 family protein, putative (Protein of unknown function, DUF538)
534523568-534526756	TraesCS4A01G226300	Protein phosphatase 2c, putative
534545306-534554553	TraesCS4A01G226400	Non-lysosomal glucosylceramidase
534780326-534790750	TraesCS4A01G226500	DNA polymerase III subunit
534790776-534791913	TraesCS4A01G226600	lysine ketoglutarate reductase trans-splicing-like protein, putative (DUF707)
534811309-534812238	TraesCS4A01G226700	Heat-shock protein, putative
535034849-535036054	TraesCS4A01G226800	Zinc finger-homeodomain protein 1
535242506-535243985	TraesCS4A01G226900	Chlorophyll a-b binding protein, chloroplastic
535244690-535248317	TraesCS4A01G227000	Glycine-rich protein
535425668-535426642	TraesCS4A01G227100	Glucuronoxylan 4-O-methyltransferase
535427916-535431632	TraesCS4A01G227200	Protein kinase-like protein
535464263-535466861	TraesCS4A01G227300	Chlorophyll a/b binding protein domain-containing protein
535819032-535819589	TraesCS4A01G227400	ethylene-responsive transcription factor
535845399-535855523	TraesCS4A01G227500	DNA-(apurinic or apyrimidinic site) lyase
535856321-535874270	TraesCS4A01G227600	ABC transporter, putative
535871696-535873014	TraesCS4A01G227700	Retrovirus-related Pol polyprotein from transposon TNT 1-94
536197440-536198627	TraesCS4A01G227800	ENTH/ANTH/VHS superfamily protein

536202329-536211191	TraesCS4A01G227900	PHD finger alfin-like protein
536327998-536328854	TraesCS4A01G228000	related to vernalization1 1
536770702-536772767	TraesCS4A01G228100	PCTP-like protein
536784520-536785344	TraesCS4A01G228200	Zinc finger MYM-type-like protein
536792875-536795577	TraesCS4A01G228300	Heparan-alpha-glucosaminide N-acetyltransferase
536820396-536820825	TraesCS4A01G228400	UPSTREAM OF FLC-like protein (DUF966)
536826088-536829987	TraesCS4A01G228500	E3 ubiquitin-protein ligase SINA-like 10
537182717-537184053	TraesCS4A01G228600	F-box/kelch-repeat protein
537231330-537232890	TraesCS4A01G228700	Transposon protein, putative, Mutator sub-class
537272548-537274101	TraesCS4A01G228800	F-box/kelch-repeat protein
537433962-537440469	TraesCS4A01G228900	Serine protease
537530140-537533999	TraesCS4A01G229000	Ankyrin repeat protein SKIP35
537543678-537544010	TraesCS4A01G229100	Argos-like protein
537592342-537596508	TraesCS4A01G229200	Hemolysin A family protein, expressed
537598043-537599866	TraesCS4A01G229300	At2g43990/F6E13.12
537949589-537957582	TraesCS4A01G229400	La
538212252-538215714	TraesCS4A01G229500	Retrovirus-related Pol polyprotein from transposon 297 family

538839324-538840786	TraesCS4A01G229600	Pectin lyase-like superfamily protein
538905249-538906763	TraesCS4A01G229700	Polygalacturonase
539126535-539129949	TraesCS4A01G229800	Receptor-like kinase
539147640-539153101	TraesCS4A01G229900	NADP-dependent alkenal double bond reductase
539156556-539160442	TraesCS4A01G230000	Acetolactate synthase small subunit
539288267-539295938	TraesCS4A01G230100	Protein transport protein SEC23
539297585-539299560	TraesCS4A01G230200	Pentatricopeptide repeat-containing protein
539299786-539302328	TraesCS4A01G230300	RNA polymerase sigma factor RpoD
539428704-539429399	TraesCS4A01G230400	Subtilisin-like protease
539583987-539585231	TraesCS4A01G230500	Calcineurin-like metallo-phosphoesterase superfamily protein
539751161-539754738	TraesCS4A01G230600	F-box/LRR protein
539755046-539761346	TraesCS4A01G230700	Plant regulator RWP-RK family protein
540014447-540017248	TraesCS4A01G230800	Transmembrane protein, putative
540058987-540060777	TraesCS4A01G230900	Glycosyltransferase family 92 protein
540416072-540417322	TraesCS4A01G231000	Glycosyltransferase family 92 protein
540433150-540437968	TraesCS4A01G231100	ER membrane protein complex subunit 10
540441364-540444518	TraesCS4A01G231200	COBRA-like protein

540600328-540604473	TraesCS4A01G231300	Homeobox-leucine zipper protein
540957727-540959091	TraesCS4A01G231400	Aspartic proteinase nepenthesin-1
541172421-541172911	TraesCS4A01G231500	cleavage and polyadenylation specificity factor 160
541337582-541340608	TraesCS4A01G231600	BSD domain containing protein, expressed
541357800-541358522	TraesCS4A01G231700	Protein MIZU-KUSSEI 1
541361828-541366225	TraesCS4A01G231800	Protein ApaG
541426996-541430264	TraesCS4A01G231900	DUF936 family protein
541672086-541679832	TraesCS4A01G232000	Dehydrogenase/reductase SDR family member
541680317-541686269	TraesCS4A01G232100	RNA-binding ASCH domain protein
541784613-541786988	TraesCS4A01G232200	Receptor-kinase, putative
541890982-541892848	TraesCS4A01G232300	NAD(P)H dehydrogenase subunit 48
541987324-541988376	TraesCS4A01G232400	Tetrapyrrole-binding protein, chloroplastic
541988817-541994144	TraesCS4A01G232500	UPF0454 protein C12orf49
542020316-542024151	TraesCS4A01G232600	Eukaryotic translation initiation factor 3 subunit B
542026261-542033155	TraesCS4A01G232700	Endonuclease III homolog
542033451-542038903	TraesCS4A01G232800	Chloroplast ATP-dependent Clp protease chaperone protein
542039991-542040407	TraesCS4A01G232900	ATP-dependent Clp protease, ATP-binding subunit

542045910-542047863	TraesCS4A01G233000	F-box protein-like protein
542248874-542250602	TraesCS4A01G233100	Kinesin-1 heavy chain
542261581-542263453	TraesCS4A01G233200	Pre-mRNA-processing protein 40A
542264619-542266973	TraesCS4A01G233300	40S ribosomal protein S27
542269885-542280729	TraesCS4A01G233400	Exocyst complex component SEC3A
542476351-542481624	TraesCS4A01G233500	ATP-dependent (S)-NAD(P)H-hydrate dehydratase
542499165-542500039	TraesCS4A01G233600	DUF1685 family protein
542532836-542533493	TraesCS4A01G233700	B3 domain-containing protein
542553417-542555908	TraesCS4A01G233800	B3 domain-containing protein
542617981-542621127	TraesCS4A01G233900	B3 domain-containing protein
542827942-542829242	TraesCS4A01G234000	Dof zinc finger protein
543332002-543333307	TraesCS4A01G234100	2-oxoglutarate (2OG) and Fe(II)-dependent oxygenase superfamily protein
543340553-543341184	TraesCS4A01G234200	Glutamine dumper, putative
543347199-543348170	TraesCS4A01G234300	Mitochondrial transcription termination factor family protein
543365697-543369614	TraesCS4A01G234400	N-acetyl-gamma-glutamyl-phosphate reductase
543374607-543375910	TraesCS4A01G234500	basic helix-loop-helix (bHLH) DNA-binding superfamily protein
543608520-543610470	TraesCS4A01G234600	Cyclin family protein

543735662-543740221	TraesCS4A01G234700	Calcium-transporting ATPase
543740661-543746036	TraesCS4A01G234800	DNA polymerase IV
544031261-544032679	TraesCS4A01G234900	F-box family protein
544033032-544036361	TraesCS4A01G235000	50S ribosomal protein L25
544046934-544047800	TraesCS4A01G235100	LOB domain family protein
544055775-544058074	TraesCS4A01G235200	Protein OSB1, mitochondrial
544058447-544060908	TraesCS4A01G235300	Oxidoreductase, aldo/keto reductase family protein, expressed
544062199-544067990	TraesCS4A01G235400	Surface presentation of antigens protein SpaR
544174235-544177981	TraesCS4A01G235500	Oxidoreductase, aldo/keto reductase family protein, expressed
544197932-544201050	TraesCS4A01G235600	Kinase family protein
544201959-544204242	TraesCS4A01G235700	Serpin family protein
544380953-544382209	TraesCS4A01G235800	Peroxidase
544389388-544390826	TraesCS4A01G235900	Serpin family protein
544397231-544397845	TraesCS4A01G236000	Zinc finger protein, putative
544887198-544891670	TraesCS4A01G236100	BTB/POZ domain-containing protein
544953084-544954778	TraesCS4A01G236200	LOB domain-containing protein, putative
545105716-545106960	TraesCS4A01G236300	Ethylene-responsive transcription factor

545114629-545117369	TraesCS4A01G236400	Fumarylacetoacetate hydrolase, putative
545124466-545124741	TraesCS4A01G236500	SWAP (Suppressor-of-White-APricot)/surp domain-containing protein / ubiquitin family protein
545192213-545193592	TraesCS4A01G236600	2-oxoglutarate (2OG) and Fe(II)-dependent oxygenase superfamily protein
545235570-545237367	TraesCS4A01G236700	Cyclin
545601815-545606948	TraesCS4A01G236800	MAR-binding filament-like protein
545761956-545762903	TraesCS4A01G236900	CCR4-NOT transcription complex subunit 7
545770348-545777493	TraesCS4A01G237000	Methionine S-methyltransferase
546005400-546005792	TraesCS4A01G237100	RNA-directed DNA polymerase; Ribonuclease H, related
546017711-546020208	TraesCS4A01G237200	Metal transporter
546210168-546212042	TraesCS4A01G237300	glutamyl-tRNA (Gln) amidotransferase subunit A (DUF620)
546256267-546259001	TraesCS4A01G237400	RING/U-box superfamily protein
546296550-546298853	TraesCS4A01G237500	Subtilisin-like protease
546477269-546483413	TraesCS4A01G237600	Ribosomal RNA small subunit methyltransferase H
546484423-546487035	TraesCS4A01G237700	A-kinase anchor protein 9, putative isoform 1
546795496-546797396	TraesCS4A01G237800	Transcriptional adapter 1
547044672-547047206	TraesCS4A01G237900	UDP-glucose 6-dehydrogenase

547200426-547201448	TraesCS4A01G238000	Zinc finger protein, putative
547203176-547204576	TraesCS4A01G238100	Presenilin
547228876-547233151	TraesCS4A01G238200	Pentatricopeptide repeat-containing protein
547300820-547303249	TraesCS4A01G238300	Mitochondrial import receptor subunit TOM5
547516971-547518642	TraesCS4A01G238400	Remorin
547928686-547930299	TraesCS4A01G238500	Adenine/guanine permease
547978932-547980325	TraesCS4A01G238600	Cysteine proteinase
547982698-547983024	TraesCS4A01G238700	Leucine-rich repeat protein kinase family protein
548053473-548056171	TraesCS4A01G238800	4-coumarate--CoA ligase like
548066534-548067145	TraesCS4A01G238900	Rho GTPase activation protein (RhoGAP) with PH domain-containing protein
548274831-548282171	TraesCS4A01G239000	4-coumarate--CoA ligase like
548341734-548344564	TraesCS4A01G239100	4-coumarate-CoA ligase
548515335-548515670	TraesCS4A01G239200	Dolichol phosphate-mannose biosynthesis regulatory protein
548518832-548527672	TraesCS4A01G239300	Exosome complex component
548523978-548525431	TraesCS4A01G239400	Retrovirus-related like polyprotein
548529652-548542423	TraesCS4A01G239500	ATP-dependent RNA helicase, putative
548774590-548779050	TraesCS4A01G239600	Receptor protein kinase-like protein

548779766-548784167	TraesCS4A01G239700	WD repeat protein-like
548819782-548822077	TraesCS4A01G239800	T-box transcription factor, putative (DUF863)
549670861-549672254	TraesCS4A01G239900	O-methyltransferase
549799338-549800138	TraesCS4A01G240000	Thioredoxin
549803298-549807400	TraesCS4A01G240100	Cycloeucalenol cycloisomerase
549816755-549820868	TraesCS4A01G240200	Receptor-like kinase
549879176-549882741	TraesCS4A01G240300	Haloacid dehalogenase-like hydrolase family protein
549922700-549924088	TraesCS4A01G240400	Phospholipase a1-chloroplastic-like
550082585-550083910	TraesCS4A01G240500	Phospholipase a1-chloroplastic-like
550324968-550328176	TraesCS4A01G240600	Mitochondrial substrate carrier family protein
550464945-550467229	TraesCS4A01G240700	Amino acid transporter family protein, putative
550476231-550480359	TraesCS4A01G240800	Thioredoxin-like protein 4B
550481163-550487426	TraesCS4A01G240900	Beta-xylosidase, putative
550488935-550489096	TraesCS4A01G241000	Ribosomal protein L2
550495384-550500502	TraesCS4A01G241100	Histidine triad nucleotide-binding protein
550501738-550508742	TraesCS4A01G241200	UBX domain containing protein, expressed
551132424-551133863	TraesCS4A01G241300	B3 domain-containing protein

551137905-551138171	TraesCS4A01G241400	O-acetylserine dependent cystathionine beta-synthase
551217304-551220548	TraesCS4A01G241500	B3 domain-containing protein
551493368-551493711	TraesCS4A01G241600	Thymidylate kinase
551764828-551772796	TraesCS4A01G241700	Eukaryotic aspartyl protease family protein
552145824-552146396	TraesCS4A01G241800	NADH dehydrogenase subunit 9
552147635-552152386	TraesCS4A01G241900	B3 domain-containing protein
552195896-552197461	TraesCS4A01G242000	B3 domain-containing protein
552214597-552214935	TraesCS4A01G242100	Retrotransposon protein, putative, unclassified
552221693-552227631	TraesCS4A01G242200	Deoxyuridine 5'-triphosphate nucleotidohydrolase
552254537-552258366	TraesCS4A01G242300	B3 domain-containing protein
552407850-552409300	TraesCS4A01G242400	B3 domain-containing protein
552409708-552413672	TraesCS4A01G242500	caspase-6 protein
552500448-552506097	TraesCS4A01G242600	Myb family transcription factor-like protein
552698535-552700166	TraesCS4A01G242700	NAC domain protein,
552816857-552818257	TraesCS4A01G242800	WD40-repeat protein
552831656-552833113	TraesCS4A01G242900	WD40-repeat protein
552877480-552877854	TraesCS4A01G243000	Transposon protein, putative, unclassified

552893219-552905734	TraesCS4A01G243100	Exocyst complex component SEC3A
553310632-553313968	TraesCS4A01G243200	RING/U-box superfamily protein
553316322-553317573	TraesCS4A01G243300	Metacaspase-1
553406089-553408630	TraesCS4A01G243400	DNA-directed RNA polymerase III subunit RPC4
553446992-553451942	TraesCS4A01G243500	Acetyltransferase component of pyruvate dehydrogenase complex
553535056-553535277	TraesCS4A01G243600	Chaperone protein dnaK
554542730-554543626	TraesCS4A01G243700	CCR4-NOT transcription complex subunit 7
554558280-554559514	TraesCS4A01G243800	CCR4-NOT transcription complex subunit 7
554629644-554630886	TraesCS4A01G243900	Polynucleotidyl transferase, ribonuclease H-like superfamily protein
554632753-554637781	TraesCS4A01G244000	WD40 repeat-like protein
554699174-554699974	TraesCS4A01G244100	Ubiquitin-conjugating enzyme E2
554840090-554850060	TraesCS4A01G244200	RING/U-box superfamily protein
554853298-554854887	TraesCS4A01G244300	UDP-glycosyltransferase
554920214-554920384	TraesCS4A01G244400	Mitochondrial import receptor subunit TOM6-like protein
554924159-554925889	TraesCS4A01G244500	Type I inositol-1,4,5-trisphosphate 5-phosphatase
554926632-554929378	TraesCS4A01G244600	Interferon-activable protein
554939208-554939585	TraesCS4A01G244700	lectin protein kinase family protein

554949269-554949565	TraesCS4A01G244800	Tetratricopeptide repeat (TPR)-like superfamily protein
554959780-554960157	TraesCS4A01G244900	lectin protein kinase family protein
554973466-554973886	TraesCS4A01G245000	K-box region and MADS-box transcription factor family protein
555440212-555441343	TraesCS4A01G245100	Dormancy-associated protein/auxin-repressed protein
555510132-555514424	TraesCS4A01G245200	Protein DA1-related 1
555515686-555523901	TraesCS4A01G245300	Protein DETOXIFICATION
555559271-555564187	TraesCS4A01G245400	Protein DETOXIFICATION
555746061-555750257	TraesCS4A01G245500	Calcineurin B-like protein
556009735-556010004	TraesCS4A01G245600	Methionine import ATP-binding protein MetN 1
556236782-556246954	TraesCS4A01G245700	Cytosolic Fe-S cluster assembly factor NBP35
556250003-556258435	TraesCS4A01G245800	RNA-binding KH domain-containing protein
556373092-556381517	TraesCS4A01G245900	p-loop containing nucleoside triphosphate hydrolases superfamily protein
556537589-556539531	TraesCS4A01G246000	WRKY transcription factor
556752351-556756416	TraesCS4A01G246100	Serine hydroxymethyltransferase
556822844-556825902	TraesCS4A01G246200	WD40 repeat-containing protein
556826154-556829503	TraesCS4A01G246300	BAT2 domain-containing protein 1
556830580-556842543	TraesCS4A01G246400	Vps51 vps67 family (Components of vesicular transport) protein

557245005-557247884	TraesCS4A01G246500	Kinase PfkB
557250646-557253918	TraesCS4A01G246600	Pentatricopeptide repeat-containing protein
557662346-557663145	TraesCS4A01G246700	Ubiquitin-conjugating enzyme E2
557729041-557733748	TraesCS4A01G246800	Poly(A) polymerase
557868542-557870242	TraesCS4A01G246900	Peptidyl-prolyl cis-trans isomerase
558210083-558213670	TraesCS4A01G247000	Cyclin-like protein
558214466-558217058	TraesCS4A01G247100	Magnesium-dependent phosphatase 1
558218404-558227155	TraesCS4A01G247200	E3 ubiquitin-protein ligase HOS1
558414331-558418509	TraesCS4A01G247300	CBS domain containing protein, expressed
558547016-558548074	TraesCS4A01G247400	Sorghum bicolor protein targeted either to mitochondria or chloroplast proteins T50848
558648418-558649786	TraesCS4A01G247500	GEM-like protein 1
558650444-558651111	TraesCS4A01G247600	ATP synthase delta-subunit protein
558655417-558656367	TraesCS4A01G247700	Syntaxin, putative
558667947-558669694	TraesCS4A01G247800	Plasma membrane ATPase
558676008-558676367	TraesCS4A01G247900	Cysteine proteinase inhibitor
558679245-558679673	TraesCS4A01G248000	Alpha amylase inhibitor protein

558845272-558848862	TraesCS4A01G248100	Methyltransferase
559044696-559047664	TraesCS4A01G248200	Endoglucanase
559048582-559049823	TraesCS4A01G248300	Pentatricopeptide repeat-containing protein
559067713-559069352	TraesCS4A01G248400	Glycerol-3-phosphate acyltransferase
559162733-559167933	TraesCS4A01G248500	Protein ZINC INDUCED FACILITATOR-LIKE 1
559486187-559487829	TraesCS4A01G248600	transmembrane protein, putative (DUF247)
559494215-559496292	TraesCS4A01G248700	Cell wall invertase
559894244-559897294	TraesCS4A01G248800	Alba DNA/RNA-binding protein
559945250-559946956	TraesCS4A01G248900	Eukaryotic translation initiation factor 3 subunit G
559949681-559950979	TraesCS4A01G249000	B3 domain-containing protein
560313977-560315500	TraesCS4A01G249100	Pentatricopeptide repeat-containing protein
560317324-560319518	TraesCS4A01G249200	RING finger and CHY zinc finger protein
560411162-560416428	TraesCS4A01G249300	Transducin/WD-like repeat-protein
560901278-560905179	TraesCS4A01G249400	GATA transcription factor
560905758-560906845	TraesCS4A01G249500	U-box domain-containing protein 57
560907883-560912041	TraesCS4A01G249600	Mitochondrial carrier protein, expressed
560951669-560951959	TraesCS4A01G249700	Alkyl hydroperoxide reductase subunit C

560964956-560965249	TraesCS4A01G249800	piezo-type mechanosensitive ion channel component
560970450-560971840	TraesCS4A01G249900	F-box domain containing protein
560994640-560996318	TraesCS4A01G250000	50S ribosomal protein L7/L12
560998060-560998512	TraesCS4A01G250100	Discoidin, CUB and LCCL domain-containing protein 1
561071827-561072185	TraesCS4A01G250200	SNF2 domain-containing protein / helicase domain-containing protein / zinc finger protein-like protein
561102971-561103327	TraesCS4A01G250300	GDP-Man:Man(3)GlcNAc(2)-PP-Dol alpha-1,2-mannosyltransferase
561105686-561106025	TraesCS4A01G250400	Protein phosphatase 2C family protein
561111934-561112400	TraesCS4A01G250500	Vacuolar protein sorting/targeting protein 10
561368126-561370971	TraesCS4A01G250600	GRF1-interacting factor-like protein
562280813-562281608	TraesCS4A01G250700	pumilio 2
562282443-562286220	TraesCS4A01G250800	Endoplasmic reticulum oxidoreductin-1
562290028-562291481	TraesCS4A01G250900	Dehydrin
562403111-562407137	TraesCS4A01G251000	Zinc finger BED domain-containing protein DAYSLEEPER
562408921-562413963	TraesCS4A01G251100	Pollen Ole e 1 allergen/extensin
562450576-562454617	TraesCS4A01G251200	Zinc finger BED domain-containing protein DAYSLEEPER
562458236-562458793	TraesCS4A01G251300	Pathogenesis-related protein 1

562482754-562484927	TraesCS4A01G251400	Kinase, putative
562573394-562574089	TraesCS4A01G251500	Protein MIZU-KUSSEI 1
563049929-563052786	TraesCS4A01G251600	4-hydroxy-3-methylbut-2-enyl diphosphate reductase
563268803-563269742	TraesCS4A01G251700	Pectinesterase
563511457-563512448	TraesCS4A01G251800	Homeobox transcription factor KN2
563677948-563679541	TraesCS4A01G251900	Sugar transporter family protein
563678635-563679387	TraesCS4A01G252000	1,4-alpha-glucan branching enzyme GlgB
564148415-564157977	TraesCS4A01G252100	Homeobox protein bel1-like protein
564163571-564167730	TraesCS4A01G252200	DNA double-strand break repair rad50 ATPase
564639556-564640280	TraesCS4A01G252300	Vacuolar sorting receptor family protein
564750385-564751806	TraesCS4A01G252400	ARM repeat superfamily protein
564951290-564953002	TraesCS4A01G252500	Peptide-N4-(N-acetyl-beta-glucosaminyl)asparagine amidase A
564954817-564956321	TraesCS4A01G252600	Glycosyltransferase
564971277-564974497	TraesCS4A01G252700	Cathepsin B-like cysteine protease
565040539-565043592	TraesCS4A01G252800	Cathepsin B-like cysteine protease
565302085-565305616	TraesCS4A01G252900	Cathepsin B-like cysteine protease
565306504-565317251	TraesCS4A01G253000	Leishmanolysin-like peptidase

565430040-565431392	TraesCS4A01G253100	F-box protein
565432182-565433195	TraesCS4A01G253200	Photosystem I reaction center subunit N
565433856-565434847	TraesCS4A01G253300	Photosystem I reaction center subunit N
565435085-565437902	TraesCS4A01G253400	Core-2/I-branching beta-1,6-N-acetylglucosaminyltransferase family protein
565699355-565725725	TraesCS4A01G253500	Calcium-transporting ATPase
565732767-565742099	TraesCS4A01G253600	Serine carboxypeptidase family protein, expressed
565747737-565750618	TraesCS4A01G253700	Serine carboxypeptidase family protein, expressed
565758829-565762061	TraesCS4A01G253800	Serine carboxypeptidase family protein, expressed
565841629-565843317	TraesCS4A01G253900	Lecithin-cholesterol acyltransferase-like 1
566289983-566291447	TraesCS4A01G254000	Lecithin-cholesterol acyltransferase-like 1
566293430-566300451	TraesCS4A01G254100	Ribosomal RNA small subunit methyltransferase F
566522605-566526929	TraesCS4A01G254200	BSD domain containing protein
566527387-566529939	TraesCS4A01G254300	ACT domain containing protein, expressed
566589200-566589644	TraesCS4A01G254400	Glutaredoxin, putative
566609902-566610346	TraesCS4A01G254500	Glutaredoxin, putative
566828841-566830249	TraesCS4A01G254600	Thioredoxin-like protein AAED1, chloroplastic
566905182-566907692	TraesCS4A01G254700	Zinc finger MYM-type-like protein

567036537-567037063		TraesCS4A01G254800	Carboxypeptidase
567037277-567038089		TraesCS4A01G254900	CVP2 like 1
567181746-567185221		TraesCS4A01G255000	Growth-regulating factor
567192867-567193136		TraesCS4A01G255100	B3 domain-containing protein
567470959-567471646		TraesCS4A01G255200	Imidazole glycerol phosphate synthase subunit HisH
567476443-567479410		TraesCS4A01G255300	Ribosomal RNA small subunit methyltransferase E
567479603-567482101	C5	TraesCS4A01G255400	Serine/threonine-protein kinase
567484301-567487389		TraesCS4A01G255500	Thioredoxin-like protein AAED1, chloroplastic
567489300-567492747		TraesCS4A01G255600	transmembrane protein
567493169-567495275	C18	TraesCS4A01G255700	ethylene-dependent gravitropism-deficient and yellow-green-like 2
567646583-567646825	*AX109496237*		
567650074-567650374	*AX109001562*		
567650160-567654340	C19	TraesCS4A01G255800	basic helix-loop-helix (bHLH) DNA-binding superfamily protein
567667591-567675909		TraesCS4A01G255900	ATP dependent RNA helicase
567676134-567678643		TraesCS4A01G256000	tRNA pseudouridine synthase
567682081-567685775		TraesCS4A01G256100	Pentatricopeptide repeat-containing protein
567686918-567691656		TraesCS4A01G256200	Chaperone protein DnaJ

567696212-567701939	TraesCS4A01G256300	GTPase obg
567706804-567708066	TraesCS4A01G256400	F-box family protein
568192908-568194707	TraesCS4A01G256500	1-aminocyclopropane-1-carboxylate synthase 1
568507891-568509412	TraesCS4A01G256600	Digeranylgeranylglyceryl phosphate synthase
569477449-569484409	TraesCS4A01G256700	homeobox-1
569866440-569868731	TraesCS4A01G256800	E3 ubiquitin-protein ligase FANCL
569869977-569874614	TraesCS4A01G256900	Alpha/beta-hydrolase superfamily protein
569877552-569878043	TraesCS4A01G257000	Epidermal patterning factor-like protein
569879972-569882429	TraesCS4A01G257100	GDP-mannose transporter
569886103-569892384	TraesCS4A01G257200	AMSH-like ubiquitin thioesterase 1
570189917-570190999	TraesCS4A01G257300	pleiotropic drug resistance 13
570262263-570267332	TraesCS4A01G257400	Sulfite exporter TauE/SafE family protein
570268678-570269961	TraesCS4A01G257500	myb-like protein X
570461424-570464905	TraesCS4A01G257600	ERD (Early-responsive to dehydration stress) family protein
570466398-570471904	TraesCS4A01G257700	Inositol-tetrakisphosphate 1-kinase
570478051-570480540	TraesCS4A01G257800	Tubulin alpha chain
570910048-570912528	TraesCS4A01G257900	basic helix-loop-helix (bHLH) DNA-binding superfamily protein

570973087-570978939	TraesCS4A01G258000	WD repeat-containing protein
570981432-570985759	TraesCS4A01G258100	Lariat debranching enzyme
570988372-570998526	TraesCS4A01G258200	Pre-mRNA polyadenylation factor Fip1
571031282-571031596	TraesCS4A01G258300	50S ribosomal protein L6, putative
571040698-571042292	TraesCS4A01G258400	Maf-like protein, expressed
571045891-571047818	TraesCS4A01G258500	Maf-like protein, expressed
571212794-571214353	TraesCS4A01G258600	dipeptide transport ATP-binding protein
571297190-571297483	TraesCS4A01G258700	Polynucleotidyl transferase, ribonuclease H-like superfamily protein
571359780-571364276	TraesCS4A01G258800	Glycine-rich family protein
571483446-571484903	TraesCS4A01G258900	Glycosyltransferase
571586153-571588969	TraesCS4A01G259000	dipeptide transport ATP-binding protein
571777303-571781968	TraesCS4A01G259100	Receptor kinase
571971770-571972432	TraesCS4A01G259200	Ethylene-responsive transcription factor
572302274-572303653	TraesCS4A01G259300	Anthocyanin 5-aromatic acyltransferase
572307556-572308866	TraesCS4A01G259400	Anthocyanin 5-aromatic acyltransferase
572535158-572539686	TraesCS4A01G259500	Sec14p-like phosphatidylinositol transfer family protein
572541723-572543895	TraesCS4A01G259600	Sec14p-like phosphatidylinositol transfer family protein

572549945-572551882	TraesCS4A01G259700	Sec14p-like phosphatidylinositol transfer family protein
572562465-572571488	TraesCS4A01G259800	Alanine--tRNA ligase
572641045-572642412	TraesCS4A01G259900	Glycosyltransferase
572649414-572650088	TraesCS4A01G260000	Glucans biosynthesis glucosyltransferase H
572702108-572705852	TraesCS4A01G260100	BTB/POZ domain-containing protein
572830903-572834487	TraesCS4A01G260200	Carbohydrate kinase-like
572836118-572837902	TraesCS4A01G260300	Pentatricopeptide repeat-containing protein
572845556-572846014	TraesCS4A01G260400	Histone H2B
572881559-572882628	TraesCS4A01G260500	late embryogenesis abundant protein
573172063-573173700	TraesCS4A01G260600	GRAS transcription factor
573316263-573318272	TraesCS4A01G260700	tetratricopeptide repeat (TPR)-containing protein
573321338-573325678	TraesCS4A01G260800	Adenylyl cyclase-associated protein
573326180-573328349	TraesCS4A01G260900	Glucan endo-1,3-beta-glucosidase
573358608-573365399	TraesCS4A01G261000	ATP-dependent DNA helicase DDM1
573367445-573367663	TraesCS4A01G261100	Myosin-J heavy chain
573665312-573669541	TraesCS4A01G261200	Short-chain dehydrogenase/reductase family protein
573670487-573671688	TraesCS4A01G261300	Phox domain-containing protein, putative isoform 1

573672265-573673733	TraesCS4A01G261400	tRNA modification GTPase MnmE
573878926-573880014	TraesCS4A01G261500	Kinase, putative
573886653-573891925	TraesCS4A01G261600	SWI/SNF complex subunit SWI3D
574158150-574159454	TraesCS4A01G261700	Protein with RNI-like/FBD-like domain
574167455-574168856	TraesCS4A01G261800	Complement decay-accelerating factor transmembrane isoform
574195994-574198659	TraesCS4A01G261900	tRNA (Ile)-lysidine synthase
574382652-574383662	TraesCS4A01G262000	Hyccin protein
574389413-574392229	TraesCS4A01G262100	actin cross-linking protein, putative (DUF569)
574524909-574526893	TraesCS4A01G262200	vacuolar protein sorting-associated protein (DUF946)
574527501-574551311	TraesCS4A01G262300	Translational activator GCN1
574765952-574766490	TraesCS4A01G262400	Heat stress transcription factor A-3
574840594-574842011	TraesCS4A01G262500	MYB transcription factor
574868896-574872931	TraesCS4A01G262600	Glutamate decarboxylase
575006274-575010252	TraesCS4A01G262700	Peptide transporter
575011484-575013303	TraesCS4A01G262800	Kinase family protein
575027251-575031340	TraesCS4A01G262900	Phytochrome
575183261-575191036	TraesCS4A01G263000	Inactive peptidyl-prolyl cis-trans isomerase shutdown

575423699-575430254	TraesCS4A01G263100	Protein kinase, putative, expressed
575735462-575737805	TraesCS4A01G263200	Alpha/beta-Hydrolases superfamily protein
575776753-575781334	TraesCS4A01G263300	Transcription factor Inducer of CBF expression 1
575798573-575801177	TraesCS4A01G263400	Helicase, putative
575802011-575802988	TraesCS4A01G263500	Alpha/beta-Hydrolases superfamily protein
575808456-575809123	TraesCS4A01G263600	Cortical cell-delineating protein
576036387-576043513	TraesCS4A01G263700	transmembrane protein, putative (DUF594)
576050429-576054653	TraesCS4A01G263800	65-kDa microtubule-associated-like protein
576157311-576166116	TraesCS4A01G263900	Dihydroxyacetone kinase family protein
576563119-576569037	TraesCS4A01G264000	Cytochrome c oxidase assembly protein ctaG
576575755-576576465	TraesCS4A01G264100	Zinc finger homeodomain protein
576590321-576596961	TraesCS4A01G264200	Cytosolic Fe-S cluster assembly factor CFD1
576602661-576610351	TraesCS4A01G264300	Ankyrin repeat-containing protein, putative
576702012-576703590	TraesCS4A01G264400	Actin
576922793-576924952	TraesCS4A01G264500	UV-B-induced protein, chloroplastic
576930523-576931965	TraesCS4A01G264600	UDP-glucose 6-dehydrogenase
577042628-577044913	TraesCS4A01G264700	Pentatricopeptide repeat-containing protein

577048828-577056229	TraesCS4A01G264800	Histidine kinase
577109148-577110539	TraesCS4A01G264900	Cytochrome b561 family protein, expressed
577115564-577116547	TraesCS4A01G265000	Expansin-like protein
577227821-577230321	TraesCS4A01G265100	Receptor protein kinase, putative
577468716-577471694	TraesCS4A01G265200	PHD finger protein family
577489413-577499663	TraesCS4A01G265300	Vam6/Vps39-like protein
577542217-577547732	TraesCS4A01G265400	Protein POLLEN DEFECTIVE IN GUIDANCE 1
577727666-577732739	TraesCS4A01G265500	Squamous cell carcinoma antigen recognized by T-cells 3
577756937-577761081	TraesCS4A01G265600	GPN-loop GTPase-like protein
577806459-577806872	TraesCS4A01G265700	Type I inositol-1,4,5-trisphosphate 5-phosphatase CVP2-like protein
577976925-577981919	TraesCS4A01G265800	RNA-binding protein 34
578007015-578008028	TraesCS4A01G265900	Protein kinase
578175175-578176188	TraesCS4A01G266000	Protein kinase
578233584-578234588	TraesCS4A01G266100	Protein kinase
578382450-578383463	TraesCS4A01G266200	protein kinase family protein
578392878-578393918	TraesCS4A01G266300	Protein kinase
578533601-578539054	TraesCS4A01G266400	NADH-ubiquinone oxidoreductase subunit

578539799-578540488	TraesCS4A01G266500	NADH-ubiquinone oxidoreductase 75 kDa subunit, mitochondrial
578540922-578547040	TraesCS4A01G266600	Dynamin
578553411-578559697	TraesCS4A01G266700	Threonine dehydratase
578573271-578574941	TraesCS4A01G266800	Pentatricopeptide repeat-containing protein
579428268-579430952	TraesCS4A01G266900	Glutamine synthetase
579431789-579438751	TraesCS4A01G267000	Phosphoglucosamine mutase
579519072-579523895	TraesCS4A01G267100	IGR motif protein
579526333-579529059	TraesCS4A01G267200	ATP-dependent Clp protease ATP-binding subunit
579609654-579612522	TraesCS4A01G267300	Receptor-like kinase
579848008-579850633	TraesCS4A01G267400	DNA (Cytosine-5-)-methyltransferase
579853617-579857109	TraesCS4A01G267500	Ubiquitin-conjugating enzyme, E2
579860024-579863556	TraesCS4A01G267600	Zinc finger protein VAR3, chloroplastic
579864436-579866269	TraesCS4A01G267700	Zinc-finger domain of monoamine-oxidase A repressor R1, putative
580288521-580301691	TraesCS4A01G267800	Kinase, putative
580302974-580304107	TraesCS4A01G267900	DNA-directed RNA polymerase subunit
580477666-580478491	TraesCS4A01G268000	Histone H1, putative
580485261-580490042	TraesCS4A01G268100	Coatomer subunit alpha

580654977-580661304	TraesCS4A01G268200	Protein kinase
580707381-580729754	TraesCS4A01G268300	Phosphatidylinositol 4-kinase
580730452-580731116	TraesCS4A01G268400	V-type proton ATPase subunit D
580734487-580735937	TraesCS4A01G268500	CONSTANS-like zinc finger protein
580895092-580903002	TraesCS4A01G268600	Protein CASP
580906525-580908057	TraesCS4A01G268700	14-3-3 protein
581142969-581144505	TraesCS4A01G268800	Accelerated cell death 11
581148192-581149603	TraesCS4A01G268900	AT-rich interactive domain protein
581150955-581151197	TraesCS4A01G269000	Acetyl-coenzyme A carboxylase carboxyl transferase subunit alpha
581191895-581195807	TraesCS4A01G269100	Protein kinase-like protein
581196871-581204088	TraesCS4A01G269200	Homologous-pairing protein 2-like protein
581200222-581201286	TraesCS4A01G269300	Regulator of Vps4 activity in the MVB pathway protein, putative
581270007-581270784	TraesCS4A01G269400	Basic blue protein
581274002-581275039	TraesCS4A01G269500	Basic blue protein
581278184-581279746	TraesCS4A01G269600	Microsomal glutathione S-transferase 3
581324889-581326814	TraesCS4A01G269700	Microsomal glutathione S-transferase 3
581329766-581330881	TraesCS4A01G269800	Microsomal glutathione S-transferase 3

581331452-581336479	TraesCS4A01G269900	Zinc finger protein VAR3, chloroplastic
581455498-581456574	TraesCS4A01G270000	DNA-binding storekeeper protein-related transcriptional regulator
581467493-581471226	TraesCS4A01G270100	Myosin-binding protein 2
581481563-581483746	TraesCS4A01G270200	ATP-dependent RNA helicase
581493890-581494297	TraesCS4A01G270300	60S ribosomal protein L5
581749794-581750715	TraesCS4A01G270400	Peptidylprolyl isomerase
581751238-581754972	TraesCS4A01G270500	3-oxo-5-alpha-steroid 4-dehydrogenase
581759713-581760915	TraesCS4A01G270600	F-box family protein
581867995-581871066	TraesCS4A01G270700	Phytanoyl-CoA dioxygenase family protein
581916832-581917610	TraesCS4A01G270800	Phospholipase A2
581934950-581944657	TraesCS4A01G270900	Glutamyl-tRNA(Gln) amidotransferase subunit A
582477716-582479578	TraesCS4A01G271000	GAI-like protein 1
582507114-582510391	TraesCS4A01G271100	Drug/metabolite transporter
582524342-582534665	TraesCS4A01G271200	Transmembrane fragile-X-F-associated protein
582839931-582840989	TraesCS4A01G271300	Transcription factor TEOSINTE BRANCHED 1
583150186-583155216	TraesCS4A01G271400	RuvB-like helicase
583162282-583163565	TraesCS4A01G271500	Fantastic four-like protein

583419318-583422909		TraesCS4A01G271600	Phosphatidylinositol-4-phosphate 5-kinase, putative
583525525-583534431		TraesCS4A01G271700	Oxysterol-binding protein, putative
583702432-583707366		TraesCS4A01G271800	Kinase family protein
583707903-583717668		TraesCS4A01G271900	Histone-lysine N-methyltransferase
583754800-583757140	C8	TraesCS4A01G272000	Plastid-lipid associated protein PAP / fibrillin family protein
583756221-583756371	*BE403251*		
583757696-583759270		TraesCS4A01G272100	30S ribosomal protein S13, putative, expressed
583760272-583763441		TraesCS4A01G272200	SNARE associated Golgi protein family
583867353-583867884		TraesCS4A01G272300	BnaA04g06320D protein
583869145-583869366		TraesCS4A01G272400	50S ribosomal protein L33
583899849-583905113		TraesCS4A01G272500	Protein kinase
583906671-583910180		TraesCS4A01G272600	Mitogen-activated protein kinase kinase kinase 1
583914291-583915891		TraesCS4A01G272700	Zinc finger family protein
583936151-583946383		TraesCS4A01G272800	Beta-glucosidase
583949402-583953409	C9	TraesCS4A01G272900	Beta-glucosidase
583949552-583949670	*BE403721*		
583959345-583960583		TraesCS4A01G273000	Ubiquitin-conjugating enzyme E2 B

583969485-583970798	TraesCS4A01G273100	Eukaryotic peptide chain release factor subunit 1-1
583975054-583980304	TraesCS4A01G273200	Nucleotide-sugar transporter family protein
584040603-584042579	TraesCS4A01G273300	Leucine-rich repeat receptor-like protein kinase family protein
584046355-584046963	TraesCS4A01G273400	S-adenosylmethionine decarboxylase proenzyme
584055111-584056903	TraesCS4A01G273500	Glycosyltransferase
584062945-584068329	TraesCS4A01G273600	Protein-protein interaction regulator
584071841-584072942	TraesCS4A01G273700	Cysteine protease, putative
584108425-584112758	TraesCS4A01G273800	Cysteine protease, putative
584111584-584111871	TraesCS4A01G273900	Cysteine protease, putative
584114748-584115922	TraesCS4A01G274000	Cysteine protease, putative
584117906-584119065	TraesCS4A01G274100	Cysteine protease, putative
584119978-584125481	TraesCS4A01G274200	Phosphatidylinositol-4-phosphate 5-kinase family protein
584272892-584276246	TraesCS4A01G274300	Ethylene receptor
584308413-584308760	TraesCS4A01G274400	transmembrane protein, putative (DUF247)
584308844-584313947	TraesCS4A01G274500	transmembrane protein, putative (DUF247)
584346191-584346772	TraesCS4A01G274600	DUF1764 domain-containing protein
584349415-584352161	TraesCS4A01G274700	RING finger protein

584382161-584387379		TraesCS4A01G274800	Retrotransposon protein, putative, unclassified
584388183-584389166		TraesCS4A01G274900	Elongation of very long chain fatty acids protein 3
584517620-584517790		TraesCS4A01G275000	RING/U-box superfamily protein
584520126-584522528		TraesCS4A01G275100	Pentatricopeptide repeat-containing protein
584523426-584525657		TraesCS4A01G275200	spindle pole body-associated protein
584532753-584535075		TraesCS4A01G275300	Haloacid dehalogenase-like hydrolase
584537677-584539036		TraesCS4A01G275400	Pre-mRNA-splicing factor 18
584543987-584553685		TraesCS4A01G275500	Importin-5
584663429-584669278		TraesCS4A01G275600	Divalent metal cation transporter MntH 2
584672019-584675743		TraesCS4A01G275700	F-box protein
584679839-584680678		TraesCS4A01G275800	Lipoxygenase
584694417-584699590		TraesCS4A01G275900	Lipoxygenase
584735545-584738842		TraesCS4A01G276000	Lipoxygenase
584783153-584785124		TraesCS4A01G276100	Laccase
584797507-584800051		TraesCS4A01G276200	N-acetyltransferase, putative
584810980-584813007		TraesCS4A01G276300	Adenylosuccinate synthetase
584817688-584823187	C10	TraesCS4A01G276400	Pescadillo homolog

584818692-584818992	*AX86179210*		
584882453-584885501		TraesCS4A01G276500	WD-repeat protein, putative
584931338-584934198		TraesCS4A01G276600	Alkaline ceramidase 3
584936954-584939511		TraesCS4A01G276700	Zinc finger CCCH domain-containing protein
584969965-584973282		TraesCS4A01G276800	Protein yippee-like
585027950-585033320		TraesCS4A01G276900	polyketide cyclase/dehydrase/lipid transport superfamily protein
585054611-585060791		TraesCS4A01G277000	Antimicrobial peptide MBP-1
585094397-585099779		TraesCS4A01G277100	Protein kinase superfamily protein
585209875-585211659		TraesCS4A01G277200	Cytokinin riboside 5'-monophosphate phosphoribohydrolase
585376976-585379886		TraesCS4A01G277300	Nuclear transcription factor Y subunit
585395987-585396268		TraesCS4A01G277400	Ran-binding protein 3
585396723-585400936		TraesCS4A01G277500	Late embryogenesis abundant hydroxyproline-rich glycoprotein
585432085-585444318		TraesCS4A01G277600	Chloride channel protein
585456231-585458315		TraesCS4A01G277700	Proteasome subunit beta type
585459229-585463234		TraesCS4A01G277800	Myelin-associated oligodendrocyte basic protein isoform 1
585539981-585541802		TraesCS4A01G277900	Cytochrome P450 family protein, expressed
585556795-585557127		TraesCS4A01G278000	RING/U-box superfamily protein

585646903-585647766	TraesCS4A01G278100	Leucine-rich repeat receptor-like protein kinase
585679342-585679971	TraesCS4A01G278200	Retrovirus-related Pol polyprotein from transposon 297 family
585687148-585693746	TraesCS4A01G278300	Ankyrin repeat family protein
586085800-586087244	TraesCS4A01G278400	Gag-pol polyprotein
586431958-586436984	TraesCS4A01G278500	Lysine--tRNA ligase
586437450-586437794	TraesCS4A01G278600	50S ribosomal protein L18
586439529-586442039	TraesCS4A01G278700	30S ribosomal protein S21
586449768-586452785	TraesCS4A01G278800	Nucleobase ascorbate transporter
586501219-586501902	TraesCS4A01G278900	Germin-like protein 1-1
586674210-586678377	TraesCS4A01G279000	Ankyrin-repeat containing protein, putative
586682057-586682725	TraesCS4A01G279100	Germin-like protein 1-1
586699223-586699897	TraesCS4A01G279200	Germin-like protein 1
586708732-586709403	TraesCS4A01G279300	Germin-like protein
586726830-586728762	TraesCS4A01G279400	Glycosyltransferase
586731120-586731695	TraesCS4A01G279500	DUF538 family protein, putative (Protein of unknown function, DUF538)
586732683-586737962	TraesCS4A01G279600	tRNA (Ile)-lysidine synthase
586857087-586858056	TraesCS4A01G279700	DEAD box RNA helicase family protein

587327316-587331588	TraesCS4A01G279800	Protein ENHANCED DISEASE RESISTANCE 2
587724356-587724601	TraesCS4A01G279900	Cytochrome bc1 complex cytochrome b subunit
588584846-588586918	TraesCS4A01G280000	Glycine-rich family protein
588633613-588643252	TraesCS4A01G280100	Heat stress transcription factor A-9
588658822-588659109	TraesCS4A01G280200	B3 domain-containing protein family
588744270-588744707	TraesCS4A01G280300	Neurogenic differentiation factor 1
588931457-588932492	TraesCS4A01G280400	Phenylalanine--tRNA ligase alpha subunit
588939567-588940103	TraesCS4A01G280500	DUF538 family protein, putative (Protein of unknown function, DUF538)
589011555-589015342	TraesCS4A01G280600	DUF538 family protein, putative (Protein of unknown function, DUF538)
589085090-589089709	TraesCS4A01G280700	Beta-1,3-galactosyltransferase-like protein
589108224-589111769	TraesCS4A01G280800	Protein UPSTREAM OF FLC
589321088-589321744	TraesCS4A01G280900	Disease resistance protein RPM1
589472934-589473664	TraesCS4A01G281000	Clavata3/ESR (CLE) gene family member
589486132-589487151	TraesCS4A01G281100	F-box family protein
589494063-589497494	TraesCS4A01G281200	Phosphatidylinositol N-acetyglucosaminlytransferase subunit P-like protein
589499333-589501718	TraesCS4A01G281300	Core-2/I-branching beta-1,6-N-acetylglucosaminyltransferase family protein
589503363-589507880	TraesCS4A01G281400	Hedgehog-interacting-like protein

589586237-589592352	TraesCS4A01G281500	Nuclease domain-containing protein 1
589600379-589601125	TraesCS4A01G281600	Octicosapeptide/Phox/Bem1p domain-containing family protein
589602948-589607173	TraesCS4A01G281700	BHLH family protein, putative, expressed
589661425-589662527	TraesCS4A01G281800	Thioesterase family protein
589777427-589780505	TraesCS4A01G281900	Receptor kinase
589787393-589791663	TraesCS4A01G282000	Serine/threonine-protein kinase
589812884-589814112	TraesCS4A01G282100	Thioesterase family protein
589835032-589836210	TraesCS4A01G282200	Thioesterase family protein
589842588-589846517	TraesCS4A01G282300	Serine/threonine-protein kinase
589878969-589879889	TraesCS4A01G282400	RNA-binding family protein isoform 1
590088220-590091470	TraesCS4A01G282500	TBC1 domain family member
590092917-590098247	TraesCS4A01G282600	FBD-associated F-box protein
590103056-590105464	TraesCS4A01G282700	Costars family protein
590107460-590112275	TraesCS4A01G282800	Tetratricopeptide repeat protein 38
590112905-590117974	TraesCS4A01G282900	Plasma membrane ATPase
590123388-590126855	TraesCS4A01G283000	B3 domain-containing protein
590180868-590186819	TraesCS4A01G283100	Plasma membrane ATPase

590204451-590236014	TraesCS4A01G283200	Inositol hexakisphosphate and diphosphoinositol-pentakisphosphate kinase
590265720-590267385	TraesCS4A01G283300	Mediator complex, subunit Med7
590277427-590280648	TraesCS4A01G283400	Calcium-dependent protein kinase
590498160-590502931	TraesCS4A01G283500	Glycine-rich family protein
590586089-590588803	TraesCS4A01G283600	Leucine-rich repeat protein kinase family protein
590590295-590593639	TraesCS4A01G283700	Hexosyltransferase
590625494-590626216	TraesCS4A01G283800	60S ribosomal protein L35a-like protein
590656013-590660306	TraesCS4A01G283900	NRT1/PTR family protein 2.2
590661142-590667513	TraesCS4A01G284000	5'-AMP-activated protein kinase subunit beta-2
590900054-590900341	TraesCS4A01G284100	NADH-ubiquinone oxidoreductase subunit
590901321-590912748	TraesCS4A01G284200	Calcineurin-binding protein cabin-1
590935529-590935893	TraesCS4A01G284300	Protein NRT1/ PTR FAMILY 5.1
590942611-590945363	TraesCS4A01G284400	GMP synthase [glutamine-hydrolyzing]
590987939-590989668	TraesCS4A01G284500	rRNA methyltransferase
590993812-590999321	TraesCS4A01G284600	Tesmin/TSO1-like CXC domain-containing protein
591070502-591074036	TraesCS4A01G284700	WAT1-related protein
591283782-591284566	TraesCS4A01G284800	Pollen allergen

591293757-591294554	TraesCS4A01G284900	Pollen allergen Phl p 5
591347606-591348302	TraesCS4A01G285000	Pollen allergen Phl p 5
591505601-591506486	TraesCS4A01G285100	Transcription elongation factor 1
591508137-591509534	TraesCS4A01G285200	Agmatine coumaroyltransferase-2
591522546-591523056	TraesCS4A01G285300	C2 calcium/lipid-binding and GRAM domain containing protein
591558800-591559460	TraesCS4A01G285400	Protein FAR1-RELATED SEQUENCE 5
591674857-591675826	TraesCS4A01G285500	Pollen allergen Phl p 5
591691741-591693220	TraesCS4A01G285600	zinc finger MYM-type-like protein
591696343-591699344	TraesCS4A01G285700	GDSL esterase/lipase
591700622-591709892	TraesCS4A01G285800	E3 ubiquitin-protein ligase
591711407-591715393	TraesCS4A01G285900	GATA transcription factor
591845836-591851939	TraesCS4A01G286000	ARM repeat superfamily protein
591854869-591856904	TraesCS4A01G286100	RecQ-mediated genome instability protein 2
591861715-591862961	TraesCS4A01G286200	Aquaporin
591864044-591866358	TraesCS4A01G286300	RecQ-mediated genome instability protein 2
591868807-591873831	TraesCS4A01G286400	Magnesium transporter MRS2-like protein
591874904-591877475	TraesCS4A01G286500	Exostosin-2

591877897-591880030	TraesCS4A01G286600	Integral membrane HPP family protein
591919611-591921968	TraesCS4A01G286700	Ferredoxin family protein, expressed
592308951-592309850	TraesCS4A01G286800	Pollen allergen Phl p 5
592334826-592335807	TraesCS4A01G286900	Pollen allergen Phl p 5
592361121-592362093	TraesCS4A01G287000	Pollen allergen Phl p 5
592486684-592491531	TraesCS4A01G287100	Hippocampus abundant transcript-like protein 1
592521252-592521762	TraesCS4A01G287200	C2 calcium/lipid-binding and GRAM domain containing protein
592543631-592547201	TraesCS4A01G287300	Peptide transporter
592762587-592763248	TraesCS4A01G287400	Protein FAR1-RELATED SEQUENCE 5
592820500-592821897	TraesCS4A01G287500	Agmatine coumaroyltransferase-2
592926089-592927358	TraesCS4A01G287600	Pollen allergen Phl p 5
593100272-593100766	TraesCS4A01G287700	Agmatine coumaroyltransferase-2
593105774-593106658	TraesCS4A01G287800	Transcription elongation factor 1
593113190-593115887	TraesCS4A01G287900	Peptide transporter
593194498-593201017	TraesCS4A01G288000	Argonaute protein
593204562-593205374	TraesCS4A01G288100	DUF674 family protein
593208070-593208944	TraesCS4A01G288200	DUF674 family protein

593278588-593284719	TraesCS4A01G288300	Argonaute family protein
593288154-593290058	TraesCS4A01G288400	Nodulin-like / Major Facilitator Superfamily protein
593333205-593337537	TraesCS4A01G288500	RNA-binding protein
593349509-593353817	TraesCS4A01G288600	WD40 repeat-like protein
593354895-593357505	TraesCS4A01G288700	Beta-xylosidase, putative
593376570-593380773	TraesCS4A01G288800	Ubiquitin-conjugating enzyme E2
593495080-593495886	TraesCS4A01G288900	rRNA N-glycosidase
593683757-593684092	TraesCS4A01G289000	Acetyl-coenzyme A carboxylase carboxyl transferase subunit beta
593732899-593733846	TraesCS4A01G289100	F-box domain containing protein
593735559-593753492	TraesCS4A01G289200	Golgi-body localization protein domain isoform 1
593761646-593766038	TraesCS4A01G289300	Serine/arginine rich splicing factor, putative
593915422-593918768	TraesCS4A01G289400	Homeobox protein BEL1 like
594002643-594004359	TraesCS4A01G289500	BEL1-like homeodomain protein
594005332-594010128	TraesCS4A01G289600	Ankyrin repeat family protein
594028246-594033990	TraesCS4A01G289700	Ankyrin repeat family protein
594039386-594040785	TraesCS4A01G289800	Leucine-rich repeat-containing protein 40
594129754-594138636	TraesCS4A01G289900	Ankyrin repeat protein-like

594140562-594147562		TraesCS4A01G290000	Ankyrin repeat family protein
594150389-594160413		TraesCS4A01G290100	Ankyrin repeat protein-like
594162606-594167272	C11	TraesCS4A01G290200	Ankyrin repeat protein-like
594165370-594165403	*BE406959*		
594180504-594186598		TraesCS4A01G290300	Ankyrin repeat family protein
594212051-594212608	*BE591356*		
594213511-594217122	C12	TraesCS4A01G290400	Phosphomethylpyrimidine synthase
594269604-594270674		TraesCS4A01G290500	Hexosyltransferase
594283074-594284324		TraesCS4A01G290600	E3 ubiquitin-protein ligase
594350894-594351879		TraesCS4A01G290700	AGAMOUS-like MADS-box transcription factor
594364173-594364763		TraesCS4A01G290800	VQ motif-containing protein
594367077-594367568		TraesCS4A01G290900	Glycine-rich protein
594378156-594378956		TraesCS4A01G291000	Thymocyte nuclear protein 1
594392621-594393043		TraesCS4A01G291100	myosin XI D
594427650-594428144		TraesCS4A01G291200	BZIP transcription factor
594441999-594443029		TraesCS4A01G291300	Neuropeptide Y receptor type 1
594460318-594460773		TraesCS4A01G291400	Fasciclin-like arabinogalactan protein 16

594533060-594535487	TraesCS4A01G291500	Growth-regulating factor
594663909-594668712	TraesCS4A01G291600	DUF21 domain-containing protein
594680010-594686220	TraesCS4A01G291700	ERD (Early-responsive to dehydration stress) family protein
594700662-594700862	TraesCS4A01G291800	double-stranded-RNA-binding protein 4
594706438-594711422	TraesCS4A01G291900	homeobox-1
594730519-594732003	TraesCS4A01G292000	Homeobox transcription factor KN2
594737676-594742642	TraesCS4A01G292100	homeobox-1
595046191-595056136	TraesCS4A01G292200	Homeobox protein knotted-1-like 1
595192015-595194917	TraesCS4A01G292300	Endo-1,4-beta-xylanase, putative, expressed
595216786-595217267	TraesCS4A01G292400	Epidermal patterning factor-like protein
595255785-595257327	TraesCS4A01G292500	Chalcone synthase
595262203-595264949	TraesCS4A01G292600	Transmembrane protein, putative
595282332-595284053	TraesCS4A01G292700	basic helix-loop-helix (bHLH) DNA-binding superfamily protein
595294357-595296747	TraesCS4A01G292800	basic helix-loop-helix (bHLH) DNA-binding superfamily protein
595329657-595352176	TraesCS4A01G292900	ADP-ribosylation factor
595359708-595360304	TraesCS4A01G293000	RNA-binding protein
595374853-595376903	TraesCS4A01G293100	NADH-ubiquinone oxidoreductase chain 5

595382839-595387139	TraesCS4A01G293200	Pentatricopeptide repeat-containing protein
595388538-595396020	TraesCS4A01G293300	Tryptophan--tRNA ligase
595391148-595393497	TraesCS4A01G293400	Hsp70-Hsp90 organizing protein 1
595417616-595419298	TraesCS4A01G293500	Glucan endo-1,3-beta-glucosidase
595422877-595424025	TraesCS4A01G293600	Ubiquitin carboxyl-terminal hydrolase-like protein
595442919-595443945	TraesCS4A01G293700	SBP (S-ribonuclease-binding protein) family protein, putative
595545817-595549141	TraesCS4A01G293800	Acidic leucine-rich nuclear phosphoprotein 32-related protein
595549815-595550617	TraesCS4A01G293900	Deoxyuridine 5'-triphosphate nucleotidohydrolase
595552210-595557131	TraesCS4A01G294000	Guanine nucleotide-binding protein subunit beta
595563706-595571876	TraesCS4A01G294100	Aminopeptidase
595655367-595659110	TraesCS4A01G294200	Zinc transporter
595661422-595663903	TraesCS4A01G294300	Zinc transporter
595817811-595821043	TraesCS4A01G294400	Regulatory protein NPR1
595844117-595846462	TraesCS4A01G294500	Transmembrane protein 56
595847372-595852760	TraesCS4A01G294600	Ras family protein
595873167-595876206	TraesCS4A01G294700	Trihelix transcription factor GT-4-like protein
595978865-595983627	TraesCS4A01G294800	Pleckstrin-like (PH) domain protein

595984431-595989792	TraesCS4A01G294900	Guanine nucleotide exchange family protein
595994746-595997670	TraesCS4A01G295000	Kinesin-like protein
596002078-596002431	TraesCS4A01G295100	DUF1677 family protein
596075859-596076185	TraesCS4A01G295200	UvrABC system protein C
596159177-596161899	TraesCS4A01G295300	senescence-associated family protein, putative (DUF581)
596165884-596166559	TraesCS4A01G295400	Acetyltransferase, GNAT family protein, expressed
596166617-596167246	TraesCS4A01G295500	Acetyltransferase, GNAT family protein, expressed
596214978-596215939	TraesCS4A01G295600	Lipid transfer protein
596221994-596223703	TraesCS4A01G295700	PHP
596238333-596239331	TraesCS4A01G295800	Lipid transfer protein
596297994-596300601	TraesCS4A01G295900	Nuclear transport factor 2 (NTF2) family protein with RNA binding (RRM-RBD-RNP motifs) domain-containing protein
596304904-596307334	TraesCS4A01G296000	Globulin 1
596446060-596448236	TraesCS4A01G296100	Globulin 1
596504054-596509118	TraesCS4A01G296200	Type I inositol-1,4,5-trisphosphate 5-phosphatase CVP2
596523171-596523860	TraesCS4A01G296300	Thaumatin-like protein
596537351-596537656	TraesCS4A01G296400	Thaumatin-like protein

596570488-596571926	TraesCS4A01G296500	Ethylene-responsive nuclear / ethylene-regulated nuclear protein (ERT2)-like protein
596624604-596628189	TraesCS4A01G296600	Tubulin beta chain
596672137-596673917	TraesCS4A01G296700	E3 ubiquitin-protein ligase
596680691-596680954	TraesCS4A01G296800	SAUR-like auxin-responsive protein family
596688333-596688617	TraesCS4A01G296900	SAUR-like auxin-responsive protein family
596713062-596713349	TraesCS4A01G297000	SAUR-like auxin-responsive protein family
596738067-596738378	TraesCS4A01G297100	SAUR-like auxin-responsive protein family
596743214-596743525	TraesCS4A01G297200	SAUR-like auxin-responsive protein family
596758261-596765940	TraesCS4A01G297300	S3 self-incompatibility locus-linked pollen protein
596767433-596769827	TraesCS4A01G297400	PH-response transcription factor pacC/RIM101 isoform 2
596770660-596773730	TraesCS4A01G297500	LOB domain protein
596789012-596792340	TraesCS4A01G297600	Pre-rRNA-processing protein TSR2
596817382-596821918	TraesCS4A01G297700	DWNN domain, a CCHC-type zinc finger
596825257-596827047	TraesCS4A01G297800	Lactoylglutathione lyase
596827940-596828380	TraesCS4A01G297900	Ferredoxin
596835928-596840310	TraesCS4A01G298000	TATA-box-binding protein

596920259-596923717		TraesCS4A01G298100	N utilization substance protein B-like protein
597013077-597015822		TraesCS4A01G298200	Glucan endo-1,3-beta-glucosidase, putative
597023208-597024827	C13	TraesCS4A01G298300	Non specific phospholipase C
597024543-597025097	*BE637642*		
597052406-597054154	C14	TraesCS4A01G298400	Cation calcium exchanger
597668209-597670977		TraesCS4A01G298500	Chaperone protein DnaJ
597671700-597672868		TraesCS4A01G298600	Heat shock family protein
597677918-597682345		TraesCS4A01G298700	5-methyltetrahydropteroyltriglutamate--homocysteine methyltransferase
597688356-597694717		TraesCS4A01G298800	Protein FAR1-RELATED SEQUENCE 5
597714516-597715616		TraesCS4A01G298900	Potassium channel
597724268-597725956		TraesCS4A01G299000	Ankyrin repeat-containing protein
597754246-597754554		TraesCS4A01G299100	Dehydrin family protein, expressed
597826624-597830284		TraesCS4A01G299200	HSP20-like chaperone superfamily protein
597833941-597837160		TraesCS4A01G299300	Membrin
597841374-597845533		TraesCS4A01G299400	Short-chain dehydrogenase/reductase family protein
597855871-597861216		TraesCS4A01G299500	Polyribonucleotide nucleotidyltransferase
597886889-597888409		TraesCS4A01G299600	Glycosyltransferase

597905256-597909732	TraesCS4A01G299700	Transmembrane protein
597912275-597916528	TraesCS4A01G299800	Cationic amino acid transporter, putative
597917105-597928087	TraesCS4A01G299900	Carboxypeptidase
597919165-597920004	TraesCS4A01G300000	nuclear pore anchor
597943888-597946110	TraesCS4A01G300100	Carboxypeptidase
597970675-597971288	TraesCS4A01G300200	Carboxypeptidase
598051227-598059177	TraesCS4A01G300300	CCR4-NOT transcription complex subunit 3
598146082-598149543	TraesCS4A01G300400	C2 calcium/lipid-binding and GRAM domain protein
598156692-598157063	TraesCS4A01G300500	calpain-type cysteine protease family
598236284-598237934	TraesCS4A01G300600	CsAtPR5
598240305-598241658	TraesCS4A01G300700	Phospholipase A1
598245669-598247060	TraesCS4A01G300800	Phospholipase A1
598294664-598296479	TraesCS4A01G300900	Phospholipase A1
598417709-598418041	TraesCS4A01G301000	Transducin/WD40 repeat-like superfamily protein
598435208-598437212	TraesCS4A01G301100	Phosphate transporter
598451057-598451640	TraesCS4A01G301200	Zinc finger protein 512B family
598453191-598455202	TraesCS4A01G301300	UDP-N-acetylmuramate--L-alanine ligase

598516610-598516873	TraesCS4A01G301400	syntaxin of plants 41
598583700-598584248	TraesCS4A01G301500	Formate-dependent phosphoribosylglycinamide formyltransferase
598590265-598592108	TraesCS4A01G301600	Methyltransferase
598601370-598603264	TraesCS4A01G301700	Xylose isomerase 1
598607823-598609741	TraesCS4A01G301800	kinase with adenine nucleotide alpha hydrolases-like domain-containing protein
598712564-598716142	TraesCS4A01G301900	Polyadenylation and cleavage factor
598719419-598726719	TraesCS4A01G302000	Protein kinase family protein
598730760-598732038	TraesCS4A01G302100	DUF868 family protein (DUF868)
598761093-598763505	TraesCS4A01G302200	Histidine-containing phosphotransfer protein
598778564-598781535	TraesCS4A01G302300	Histidine-containing phosphotransfer protein
598791728-598805534	TraesCS4A01G302400	Histidine-containing phosphotransfer protein
598807712-598810145	TraesCS4A01G302500	Histidine-containing phosphotransfer protein
598813075-598815848	TraesCS4A01G302600	Histidine-containing phosphotransfer protein
599082111-599084563	TraesCS4A01G302700	Histidine-containing phosphotransfer protein
599098473-599102373	TraesCS4A01G302800	Histidine-containing phosphotransfer protein
599123334-599125774	TraesCS4A01G302900	Histidine-containing phosphotransfer protein

599156292-599158592	TraesCS4A01G303000	Histidine-containing phosphotransfer protein
599219291-599223173	TraesCS4A01G303100	Histidine-containing phosphotransfer protein
599245371-599247813	TraesCS4A01G303200	Histidine-containing phosphotransfer protein
599318185-599321542	TraesCS4A01G303300	Monodehydroascorbate reductase
599321764-599326557	TraesCS4A01G303400	Aldo-keto reductase/ oxidoreductase
599348646-599351947	TraesCS4A01G303500	Histidine-containing phosphotransfer protein
599467504-599470183	TraesCS4A01G303600	GDSL esterase/lipase
599586672-599586953	TraesCS4A01G303700	50S ribosomal protein L23
599587932-599588347	TraesCS4A01G303800	UDP-N-acetylglucosamine 1-carboxyvinyltransferase
599611950-599613428	TraesCS4A01G303900	3-ketoacyl-CoA synthase
599620398-599621905	TraesCS4A01G304000	HXXXD-type acyl-transferase family protein, putative
599628039-599630576	TraesCS4A01G304100	Histidine-containing phosphotransfer protein
599643580-599646362	TraesCS4A01G304200	Histidine-containing phosphotransfer protein
599729672-599732995	TraesCS4A01G304300	GDSL esterase/lipase
599842221-599846313	TraesCS4A01G304400	Fatty acyl-CoA reductase
599887495-599892991	TraesCS4A01G304500	Protein HIRA
599893273-599897686	TraesCS4A01G304600	GDSL esterase/lipase

600387015-600387904		TraesCS4A01G304700	GDSL esterase/lipase
600465221-600466069		TraesCS4A01G304800	F-box domain containing protein
600515322-600517013		TraesCS4A01G304900	Gag polyprotein
600557899-600558255		TraesCS4A01G305000	acetyl Co-enzyme a carboxylase carboxyltransferase alpha subunit
600562291-600562677		TraesCS4A01G305100	PP2C protein (Clade A protein phosphatases type 2C)
600623641-600624018		TraesCS4A01G305200	Voltage-dependent calcium channel gamma-3 subunit
600898793-600901403		TraesCS4A01G305300	Ribosomal protein
600902966-600906006		TraesCS4A01G305400	R3h domain containing protein, putative
600912003-600913370		TraesCS4A01G305500	Leucine-rich repeat receptor-like protein kinase family protein
600913413-600914954	C1	TraesCS4A01G305600	Leucine-rich repeat receptor-like protein kinase family protein
600917368-600917668	*AX109895154*		
600918067-600918777	C2	TraesCS4A01G305700	Leucine-rich repeat receptor-like protein kinase family protein
600918976-600919929		TraesCS4A01G305800	Leucine-rich repeat receptor-like protein kinase family protein
600973780-600976674		TraesCS4A01G305900	Leucine-rich repeat receptor-like protein kinase family protein
601006374-601007264		TraesCS4A01G306000	Leucine-rich repeat receptor-like protein kinase family protein
601015641-601018036		TraesCS4A01G306100	Leucine-rich repeat receptor-like protein kinase family protein
601032932-601035720		TraesCS4A01G306200	Leucine-rich repeat receptor-like protein kinase family protein

601039945-601041268	TraesCS4A01G306300	Sulfate adenylyltransferase subunit 2
601042166-601044648	TraesCS4A01G306400	Nuclear transcription factor Y subunit B
601059795-601062569	TraesCS4A01G306500	Ubiquitin
601156437-601157280	TraesCS4A01G306600	Germin-like protein
601170067-601170582	TraesCS4A01G306700	Germin-like protein
601178223-601178885	TraesCS4A01G306800	Germin-like protein
601180599-601181212	TraesCS4A01G306900	Germin-like protein
601194129-601194761	TraesCS4A01G307000	Germin-like protein
601196302-601197426	TraesCS4A01G307100	40S ribosomal protein S25, putative
601243050-601248526	TraesCS4A01G307200	Hexosyltransferase
601276423-601279582	TraesCS4A01G307300	Copper ion-binding protein
601283765-601284744	TraesCS4A01G307400	Dirigent protein
601309805-601313807	TraesCS4A01G307500	Receptor protein kinase, putative
601337777-601338814	TraesCS4A01G307600	Cytochrome P450 family protein, expressed
601338817-601339314	TraesCS4A01G307700	Cytochrome P450 family protein, expressed
601356729-601357874	TraesCS4A01G307800	Haloacid dehalogenase-like hydrolase superfamily protein
601364149-601367728	TraesCS4A01G307900	Beta-amylase

601691310-601691909	TraesCS4A01G308000	DNA-directed RNA polymerase subunit
601721962-601724493	TraesCS4A01G308100	Queuine tRNA-ribosyltransferase
602053534-602061759	TraesCS4A01G308200	ABC transporter G family member
602106173-602113523	TraesCS4A01G308300	NBS-LRR disease resistance protein-like protein
602114965-602118639	TraesCS4A01G308400	Sn1-specific diacylglycerol lipase alpha
602131245-602131586	TraesCS4A01G308500	Endosperm transfer cell specific PR9
602248955-602250749	TraesCS4A01G308600	Cytochrome P450 family protein, expressed
602260036-602261169	TraesCS4A01G308700	2-oxoglutarate (2OG) and Fe(II)-dependent oxygenase superfamily protein
602269708-602272129	TraesCS4A01G308800	Cytochrome P450
602272639-602274102	TraesCS4A01G308900	Glycosyltransferase
602306511-602307929	TraesCS4A01G309000	Glycosyltransferase
602379677-602381627	TraesCS4A01G309100	F-box family protein
602621396-602653205	TraesCS4A01G309200	receptor kinase 1
602797611-602798738	TraesCS4A01G309300	Cysteine protease-like protein
602863275-602864408	TraesCS4A01G309400	2-oxoglutarate (2OG) and Fe(II)-dependent oxygenase superfamily protein
602870810-602873131	TraesCS4A01G309500	NBS-LRR-like resistance protein
602873144-602875409	TraesCS4A01G309600	NBS-LRR-like resistance protein

602898874-602900427	TraesCS4A01G309700	Disease resistance protein RGA2
602951973-602952419	TraesCS4A01G309800	nucleolar-like protein
603030535-603031895	TraesCS4A01G309900	Wall-associated receptor kinase 2
603031991-603032776	TraesCS4A01G310000	Receptor-like protein kinase
603034418-603038171	TraesCS4A01G310100	Receptor-like protein kinase
603043878-603048236	TraesCS4A01G310200	Disease resistance protein (NBS-LRR class) family
603124352-603125053	TraesCS4A01G310300	Ta11-like non-LTR retrotransposon
603281751-603283618	TraesCS4A01G310400	ACT domain-containing protein
603340065-603340286	TraesCS4A01G310500	Powder tolerance-related protein
603372855-603375220	TraesCS4A01G310600	PsbP-like protein 1
603377077-603380232	TraesCS4A01G310700	C2H2-like zinc finger protein, putative
603382876-603384074	TraesCS4A01G310800	Protein kinase, putative
603389076-603390446	TraesCS4A01G310900	F-box family protein
603405890-603409736	TraesCS4A01G311000	2-oxoglutarate (2OG) and Fe(II)-dependent oxygenase superfamily protein
603446017-603450038	TraesCS4A01G311100	Poly(A) RNA polymerase GLD2-A
603451508-603455462	TraesCS4A01G311200	RING/U-box superfamily protein
603458896-603462620	TraesCS4A01G311300	Phosphate transporter

603464816-603468137	TraesCS4A01G311400	Short-chain dehydrogenase/reductase family protein
603469204-603469938	TraesCS4A01G311500	F-box family protein
603481988-603482257	TraesCS4A01G311600	Histone H2B
603487771-603490362	TraesCS4A01G311700	ACT domain-containing protein
603497203-603499122	TraesCS4A01G311800	Protein UPSTREAM OF FLC
603504251-603504949	TraesCS4A01G311900	Antimicrobial peptide MBP-1 related (LEM1)
603506946-603507572	TraesCS4A01G312000	TRAF-like superfamily protein
603511086-603513774	TraesCS4A01G312100	Hydroxyproline-rich glycoprotein family protein
603531246-603533012	TraesCS4A01G312200	mesoderm induction early response protein
603533796-603535084	TraesCS4A01G312300	Calcium-dependent lipid-binding domain protein
603539694-603540551	TraesCS4A01G312400	AT hook motif DNA-binding family protein
603634761-603635540	TraesCS4A01G312500	LOB domain-containing protein, putative
603637548-603638907	TraesCS4A01G312600	Peptidyl-prolyl cis-trans isomerase
603666136-603667011	TraesCS4A01G312700	Aquaporin-like protein
603674525-603681868	TraesCS4A01G312800	Disease resistance protein (NBS-LRR class) family
603698121-603703864	TraesCS4A01G312900	Disease resistance protein (NBS-LRR class) family
603705420-603706858	TraesCS4A01G313000	Retrovirus-related Pol polyprotein from transposon TNT 1-94

603726600-603729539	TraesCS4A01G313100	Leucine-rich repeat receptor-like protein kinase family protein
603980200-603981915	TraesCS4A01G313200	Flavin-containing monooxygenase
604055487-604056041	TraesCS4A01G313300	AWPM-19-like membrane family protein
604068519-604069061	TraesCS4A01G313400	AWPM-19-like membrane family protein
604076529-604093392	TraesCS4A01G313500	Myosin
604101314-604103592	TraesCS4A01G313600	Ubiquitin-conjugating enzyme E2
604104116-604105207	TraesCS4A01G313700	2-oxoglutarate (2OG) and Fe(II)-dependent oxygenase superfamily protein, putative
604107899-604111305	TraesCS4A01G313800	B3 domain-containing protein
604208547-604211960	TraesCS4A01G313900	Receptor-like protein kinase
604594773-604595687	TraesCS4A01G314000	2-oxoglutarate (2OG) and Fe(II)-dependent oxygenase superfamily protein
604659700-604661064	TraesCS4A01G314100	Anthocyanin 5-aromatic acyltransferase
604672359-604672679	TraesCS4A01G314200	Receptor-like kinase
604772917-604775106	TraesCS4A01G314300	Disease resistance protein RPM1
604847255-604850213	TraesCS4A01G314400	Disease resistance protein RPM1
604856722-604857722	TraesCS4A01G314500	Ethylene-responsive transcription factor
604940612-604941666	TraesCS4A01G314600	Ethylene-responsive transcription factor

605011157-605019060	TraesCS4A01G314700	BTB/POZ/MATH-domain protein
605024348-605027787	TraesCS4A01G314800	transcription factor, putative (Protein of unknown function, DUF547)
605085385-605087313	TraesCS4A01G314900	Sugar transporter, putative
605135482-605137364	TraesCS4A01G315000	Serine/threonine-protein phosphatase
605559706-605576513	TraesCS4A01G315100	Protein kinase
605640606-605646822	TraesCS4A01G315200	OTU domain-containing protein
605644790-605646059	TraesCS4A01G315300	Trihelix transcription factor
605650689-605651247	TraesCS4A01G315400	Early nodulin-like protein
605656378-605659792	TraesCS4A01G315500	60 kDa chaperonin
605663621-605667985	TraesCS4A01G315600	Mediator of RNA polymerase II transcription subunit 15a
605711438-605714093	TraesCS4A01G315700	RNA polymerase sigma factor
605717729-605733179	TraesCS4A01G315800	Mediator of RNA polymerase II transcription subunit 13
605744972-605749290	TraesCS4A01G315900	ABC transporter G family member
606116328-606116585	TraesCS4A01G316000	Cytoplasmic polyadenylation element-binding protein 4
606339516-606340395	TraesCS4A01G316100	F-box family protein
606344404-606347550	TraesCS4A01G316200	Heavy metal-associated protein
606361307-606365891	TraesCS4A01G316300	La-related protein

606370348-606374153	TraesCS4A01G316400	GDSL esterase/lipase
606397501-606401304	TraesCS4A01G316500	GDSL esterase/lipase
606411251-606412810	TraesCS4A01G316600	Protein DETOXIFICATION
606523492-606525117	TraesCS4A01G316700	Avr9/Cf-9 rapidly elicited protein
606536601-606541208	TraesCS4A01G316800	WD40 repeat-containing protein
606586469-606587515	TraesCS4A01G316900	phosphotransferases/inositol or phosphatidylinositol kinase
606591102-606591425	TraesCS4A01G317000	Bifunctional inhibitor/lipid-transfer protein/seed storage 2S albumin-like protein
606593204-606593985	TraesCS4A01G317100	DNA translocase FtsK
606608746-606609752	TraesCS4A01G317200	Harpin-induced protein 1 (Hin1), putative
606644043-606645527	TraesCS4A01G317300	Galactose-6-phosphate isomerase subunit LacB
606647800-606648237	TraesCS4A01G317400	Mannonate dehydratase
606761195-606779668	TraesCS4A01G317500	Kinase family protein
606796975-606806270	TraesCS4A01G317600	Kinase family protein
606811790-606812212	TraesCS4A01G317700	Oxidoreductase/transition metal ion-binding protein
607045527-607055050	TraesCS4A01G317800	Exportin-1
607177428-607178300	TraesCS4A01G317900	Cytokinin riboside 5'-monophosphate phosphoribohydrolase

607178925-607179542		TraesCS4A01G318000	Plant invertase/pectin methylesterase inhibitor superfamily
607261315-607262061		TraesCS4A01G318100	Cytokinin riboside 5'-monophosphate phosphoribohydrolase
607270018-607270363		TraesCS4A01G318200	F-box only 46
607271201-607274122		TraesCS4A01G318300	Leucine-rich repeat receptor-like protein kinase family protein
607309139-607310644		TraesCS4A01G318400	ATP-dependent zinc metalloprotease FtsH
607374419-607374938		TraesCS4A01G318500	DNA topoisomerase
607378184-607378939		TraesCS4A01G318600	EMBRYO SURROUNDING FACTOR 1-like protein 8
607417310-607417758		TraesCS4A01G318700	Pentatricopeptide repeat-containing protein At1g19720
607427642-607428537		TraesCS4A01G318800	carbohydrate esterase, putative (DUF303)
607432191-607434672		TraesCS4A01G318900	NAD/NADP-dependent betaine aldehyde dehydrogenase
607633308-607635652	C3	TraesCS4A01G319000	PGR5-like protein 1A, chloroplastic
607888036-607888131	*BV211529*		
608043668-608044864		TraesCS4A01G319100	Gibberellin 20 oxidase
608260037-608261317		TraesCS4A01G319200	F-box protein PP2
608267651-608271046	C4	TraesCS4A01G319300	Disease resistance protein (NBS-LRR class) family
608478075-608481479		TraesCS4A01G319400	Disease resistance protein (NBS-LRR class) family
608554446-608556572		TraesCS4A01G319500	Pentatricopeptide repeat-containing protein

608558256-608560511	TraesCS4A01G319600	Coatomer subunit zeta
608560774-608562889	TraesCS4A01G319700	Dehydrogenase/reductase SDR family protein 7-like protein
608602654-608609084	TraesCS4A01G319800	ATP-dependent DNA helicase 2 subunit KU80
608610038-608610918	TraesCS4A01G319900	Acyl carrier protein
608828643-608834674	TraesCS4A01G320000	Sucrose nonfermenting 4-like protein
608836776-608838380	TraesCS4A01G320100	2-oxoglutarate (2OG) and Fe(II)-dependent oxygenase superfamily protein
608866790-608868195	TraesCS4A01G320200	Ribosome hibernation promotion factor
608879224-608880208	TraesCS4A01G320300	DNA-(apurinic or apyrimidinic site) lyase
608881132-608882197	TraesCS4A01G320400	F-box protein PP2
608983186-608987723	TraesCS4A01G320500	Outer envelope pore protein 24B, chloroplastic
608990462-608990752	TraesCS4A01G320600	RADIALIS-like transcription factor
609070959-609072655	TraesCS4A01G320700	F-box protein
609080072-609084634	TraesCS4A01G320800	WRKY transcription factor
609132923-609133702	TraesCS4A01G320900	RING/U-box superfamily protein
609182691-609183530	TraesCS4A01G321000	Protein YIPF
609416867-609417226	TraesCS4A01G321100	Retrotransposon protein, putative, unclassified, expressed
609564170-609566524	TraesCS4A01G321200	RING/U-box superfamily protein, putative

609804687-609807321	TraesCS4A01G321300	Poly [ADP-ribose] polymerase
609815821-609818981	TraesCS4A01G321400	Beta-fructofuranosidase, insoluble protein
609917868-609922743	TraesCS4A01G321500	Beta-fructofuranosidase, insoluble protein
609926193-609929495	TraesCS4A01G321600	Beta-fructofuranosidase, insoluble protein
610034075-610036709	TraesCS4A01G321700	Cell wall invertase
610041237-610042322	TraesCS4A01G321800	Xyloglucan endotransglucosylase/hydrolase
610089743-610090656	TraesCS4A01G321900	F-box family protein
610130944-610132777	TraesCS4A01G322000	F-box family protein
610250073-610252980	TraesCS4A01G322100	SPFH domain/band 7 family protein
610261683-610263056	TraesCS4A01G322200	Myb factor
610484185-610490550	TraesCS4A01G322300	Heat shock transcription factor family protein
610493560-610496978	TraesCS4A01G322400	RNA-binding family protein, putative
610497225-610500400	TraesCS4A01G322500	Polyribonucleotide nucleotidyltransferase, putative
610618883-610629153	TraesCS4A01G322600	E3 ubiquitin-protein ligase listerin
610640193-610642379	TraesCS4A01G322700	F-box family protein
610706805-610709026	TraesCS4A01G322800	F-box family protein
610816015-610818670	TraesCS4A01G322900	F-box family protein

611025042-611028000	TraesCS4A01G323000	F-box family protein
611029706-611030686	TraesCS4A01G323100	Retrovirus-related Pol polyprotein from transposon TNT 1-94
611071297-611073599	TraesCS4A01G323200	F-box family protein
611201294-611203594	TraesCS4A01G323300	F-box family protein
611264184-611266257	TraesCS4A01G323400	Receptor protein kinase, putative
611268602-611271640	TraesCS4A01G323500	Protein kinase
611278023-611278475	TraesCS4A01G323600	30S ribosomal protein S9
611579698-611583177	TraesCS4A01G323700	ADP-ribosylation factor GTPase-activating protein
611592308-611592874	TraesCS4A01G323800	AIG2-like (Avirulence induced gene) family protein
611656148-611656933	TraesCS4A01G323900	WPP domain associated protein
611687436-611690317	TraesCS4A01G324000	Protein BCCIP homolog
611932162-611935806	TraesCS4A01G324100	ATP-dependent Clp protease ATP-binding subunit
611976530-611977103	TraesCS4A01G324200	ATP-dependent Clp protease ATP-binding subunit ClpB
612040266-612041864	TraesCS4A01G324300	Anthocyanin 3'-O-beta-glucosyltransferase
612059812-612061668	TraesCS4A01G324400	Transmembrane 70, mitochondrial
612062177-612065071	TraesCS4A01G324500	GC-rich sequence DNA-binding factor 1
612072756-612078364	TraesCS4A01G324600	Ribonuclease P protein subunit P38, related protein

612079125-612084522	TraesCS4A01G324700	Protein BREVIS RADIX
612110333-612114060	TraesCS4A01G324800	Cleavage and polyadenylation specificity factor subunit 3
612372451-612392100	TraesCS4A01G324900	Methyl-CpG-binding domain-containing protein 9
612426443-612433137	TraesCS4A01G325000	Ankyrin repeat family protein
612442435-612443827	TraesCS4A01G325100	Methyl-CpG-binding domain-containing protein 9
612531673-612533481	TraesCS4A01G325200	F-box family protein
613266770-613268275	TraesCS4A01G325300	Pentatricopeptide repeat-containing protein
613268358-613271776	TraesCS4A01G325400	Flavin-containing monooxygenase
613319821-613321985	TraesCS4A01G325500	Ycf20-like protein
613322410-613325605	TraesCS4A01G325600	Tumor necrosis factor receptor superfamily member 21
613330067-613333466	TraesCS4A01G325700	Receptor-like protein kinase
613367368-613367595	TraesCS4A01G325800	Hydroxyproline-rich glycoprotein
613398947-613400085	TraesCS4A01G325900	Protein kinase family protein
613401832-613406707	TraesCS4A01G326000	Nuclear factor Y, putative
613412237-613416220	TraesCS4A01G326100	Derlin
613417884-613419340	TraesCS4A01G326200	Pentatricopeptide repeat-containing protein, putative
613422860-613423415	TraesCS4A01G326300	Hydroxyproline-rich glycoprotein family protein

613543523-613544365	TraesCS4A01G326400	Ethylene-responsive transcription factor
613559481-613562327	TraesCS4A01G326500	Epstein-Barr virus EBNA-1-like protein
613568109-613568616	TraesCS4A01G326600	Hydroxyproline-rich glycoprotein family protein
613581481-613582994	TraesCS4A01G326700	Acyl-[acyl-carrier-protein] desaturase
613606291-613615092	TraesCS4A01G326800	Transducin/WD-like repeat-protein
613667357-613668904	TraesCS4A01G326900	Organic cation/carnitine transporter
613697285-613704538	TraesCS4A01G327000	carboxyl-terminal peptidase, putative (DUF239)
613740671-613742242	TraesCS4A01G327100	Receptor-like protein kinase
613742362-613744101	TraesCS4A01G327200	Leucine-rich repeat receptor kinase-like protein
613765165-613767888	TraesCS4A01G327300	Disease resistance protein RPM1
613804119-613812765	TraesCS4A01G327400	carboxyl-terminal peptidase, putative (DUF239)
613816530-613819414	TraesCS4A01G327500	Protein transport protein Sec61 subunit alpha
613855345-613861730	TraesCS4A01G327600	carboxyl-terminal peptidase, putative (DUF239)
613996678-613998876	TraesCS4A01G327700	Transposon protein, putative, Mutator sub-class
613999134-613999892	TraesCS4A01G327800	Transposon protein, putative, Mutator sub-class
614004317-614006188	TraesCS4A01G327900	Tropinone reductase-like protein
614014654-614016256	TraesCS4A01G328000	Tropinone reductase-like protein

614018608-614019552	TraesCS4A01G328100	Aspartic proteinase nepenthesin-1
614027490-614029756	TraesCS4A01G328200	Pantothenate synthetase
614029976-614030492	TraesCS4A01G328300	mechanosensitive channel of small conductance-like 9
614031503-614033643	TraesCS4A01G328400	Pentatricopeptide repeat-containing protein
614101649-614103184	TraesCS4A01G328500	Pentatricopeptide repeat-containing protein
614106743-614108525	TraesCS4A01G328600	Ankyrin repeat domain protein, putative
614111306-614116597	TraesCS4A01G328700	Transducin/WD-like repeat-protein
614228693-614230620	TraesCS4A01G328800	Laccase
614241223-614243111	TraesCS4A01G328900	cobalt ion-binding protein
614244840-614247194	TraesCS4A01G329000	FRIGIDA-like protein, putative
614252518-614258300	TraesCS4A01G329100	Disease resistance protein (NBS-LRR class) family
614401759-614406887	TraesCS4A01G329200	Protein ZINC INDUCED FACILITATOR-LIKE 1
614411595-614415624	TraesCS4A01G329300	U2 small nuclear ribonucleoprotein A
614416477-614426973	TraesCS4A01G329400	E3 ubiquitin-protein ligase SspH2
614609275-614612070	TraesCS4A01G329500	DEAD-box ATP-dependent RNA helicase 52A
614867396-614871236	TraesCS4A01G329600	26S proteasome non-ATPase regulatory subunit
614906939-614908430	TraesCS4A01G329700	F-box family protein

615032206-615040140	TraesCS4A01G329800	Protein kinase family protein
615099611-615101105	TraesCS4A01G329900	F-box family protein
615104066-615106167	TraesCS4A01G330000	protein kinase family protein
615106331-615107512	TraesCS4A01G330100	Leucine-rich repeat receptor-like protein kinase family protein
615193675-615198039	TraesCS4A01G330200	Monothiol glutaredoxin
615205524-615209815	TraesCS4A01G330300	Glutaredoxin
615212565-615216245	TraesCS4A01G330400	Elongation factor Tu
615219045-615222583	TraesCS4A01G330500	BED zinc finger,hAT family dimerization domain
615362649-615364826	TraesCS4A01G330600	F-box/RNI-like/FBD-like domains-containing protein
615372042-615372374	TraesCS4A01G330700	Rust resistance-like protein RP1
615437069-615439255	TraesCS4A01G330800	Shikimate dehydrogenase (NADP(+))
615445461-615447633	TraesCS4A01G330900	LRR-RLK
615455264-615457337	TraesCS4A01G331000	F-box family protein
615694100-615702325	TraesCS4A01G331100	Pleiotropic drug resistance ABC transporter
615941151-615942218	TraesCS4A01G331200	Dolichyl-diphosphooligosaccharide--protein glycosyltransferase subunit DAD1
615969199-615971265	TraesCS4A01G331300	Cytochrome b

616086874-616109275	TraesCS4A01G331400	meristematic receptor-like kinase
616229091-616229877	TraesCS4A01G331500	F-box domain containing protein
616230369-616234110	TraesCS4A01G331600	Alcohol dehydrogenase-like protein
616236683-616237678	TraesCS4A01G331700	F-box domain containing protein
616305755-616306168	TraesCS4A01G331800	18S pre-ribosomal assembly protein gar2-like protein
616310241-616313839	TraesCS4A01G331900	Cinnamoyl-CoA reductase 4
616501029-616501661	TraesCS4A01G332000	Serrate RNA effector molecule homolog
616507393-616508992	TraesCS4A01G332100	Nascent polypeptide-associated complex subunit beta
616611347-616612869	TraesCS4A01G332200	F-box and associated interaction domains-containing protein
616617242-616618711	TraesCS4A01G332300	F-box family protein
616662076-616664226	TraesCS4A01G332400	F-box family protein
616673096-616674421	TraesCS4A01G332500	F-box domain containing protein, expressed
616700796-616703444	TraesCS4A01G332600	FBD-associated F-box protein
616839912-616876772	TraesCS4A01G332700	F-box and associated interaction domains-containing protein
616875589-616876473	TraesCS4A01G332800	F-box domain containing protein
616884760-616886057	TraesCS4A01G332900	F-box and associated interaction domains-containing protein
616896068-616897536	TraesCS4A01G333000	F-box family protein

616900752-616903264	TraesCS4A01G333100	F-box family protein
616961344-616961814	TraesCS4A01G333200	Basic blue protein, putative
616977285-616977749	TraesCS4A01G333300	Basic blue protein
616981393-616985103	TraesCS4A01G333400	Transferase, transferring glycosyl groups
617001184-617002438	TraesCS4A01G333500	Sec14p-like phosphatidylinositol transfer family protein
617042496-617050788	TraesCS4A01G333600	Sec14p-like phosphatidylinositol transfer family protein
617049263-617049826	TraesCS4A01G333700	Ethylene-responsive transcription factor
617094599-617097896	TraesCS4A01G333800	Sec14p-like phosphatidylinositol transfer family protein
617230785-617234228	TraesCS4A01G333900	KH domain-containing protein
617281529-617282683	TraesCS4A01G334000	Glycine rich protein 3
617288987-617290506	TraesCS4A01G334100	Glycine rich protein 3
617349130-617350011	TraesCS4A01G334200	Glycine rich protein 3
617360412-617361293	TraesCS4A01G334300	Glycine rich protein 3
617445072-617446375	TraesCS4A01G334400	Integral membrane protein
617652694-617654247	TraesCS4A01G334500	Sucrose transporter
617657626-617659105	TraesCS4A01G334600	U3 small nucleolar RNA-associated protein 18-like protein
617849876-617853865	TraesCS4A01G334700	Actin-related protein 2/3 complex subunit 4

617856078-617859609	TraesCS4A01G334800	B-cell receptor-associated protein 31-like containing protein
617907195-617912173	TraesCS4A01G334900	Protein kinase family protein
617924650-617927527	TraesCS4A01G335000	lectin-receptor kinase
617930763-617932013	TraesCS4A01G335100	Zinc finger MYM-type-like protein
617933919-617940601	TraesCS4A01G335200	Protein kinase family protein
617942110-617944974	TraesCS4A01G335300	RPM1-interacting protein 4
617945575-617951208	TraesCS4A01G335400	Conserved oligomeric Golgi complex subunit 2
618084665-618089234	TraesCS4A01G335500	NBS-LRR disease resistance protein
618121544-618125040	TraesCS4A01G335600	NBS-LRR-like resistance protein
618158574-618175415	TraesCS4A01G335700	receptor kinase 1
618265643-618275366	TraesCS4A01G335800	Ankyrin repeat family protein
618411430-618413166	TraesCS4A01G335900	Pirin-like protein
618417500-618419188	TraesCS4A01G336000	Pirin-like protein
618463034-618464665	TraesCS4A01G336100	Pirin-like protein
618528523-618530057	TraesCS4A01G336200	Pirin-like protein
618564906-618567847	TraesCS4A01G336300	Nuclear RNA binding protein, putative
618620274-618627946	TraesCS4A01G336400	Long-chain-fatty-acid CoA ligase, putative

618660637-618674099	TraesCS4A01G336500	Long-chain-fatty-acid CoA ligase, putative
619147079-619160948	TraesCS4A01G336600	Cleavage and polyadenylation specificity factor subunit 3
619181209-619181706	TraesCS4A01G336700	B3 domain-containing protein
619184361-619188525	TraesCS4A01G336800	Kinase-like protein
619278543-619284761	TraesCS4A01G336900	Ubiquitin carboxyl-terminal hydrolase-related protein
619312282-619312990	TraesCS4A01G337000	CRIB domain-containing protein RIC4
619333716-619334321	TraesCS4A01G337100	CRIB domain-containing protein RIC4
619370063-619370655	TraesCS4A01G337200	ROP-interactive CRIB motif protein
619372946-619375424	TraesCS4A01G337300	UDP-4-amino-4-deoxy-L-arabinose--oxoglutarate aminotransferase
619582736-619583677	TraesCS4A01G337400	U3 small nucleolar RNA-associated protein 18-like protein
619590939-619591567	TraesCS4A01G337500	Serine/threonine-protein phosphatase 7 long form like
619686152-619686415	TraesCS4A01G337600	RING/FYVE/PHD-type zinc finger family protein
619948588-619950250	TraesCS4A01G337700	U3 small nucleolar RNA-associated protein 18-like protein
620189006-620194905	TraesCS4A01G337800	Aspartokinase
620260277-620261490	TraesCS4A01G337900	zinc finger/BTB domain protein, putative (DUF1644)
620289827-620290336	TraesCS4A01G338000	Invertase inhibitor
620323514-620325316	TraesCS4A01G338100	Pectinesterase

638240325-638241099	C6	TraesCS4A01G365100	SHAGGY-like kinase
638661695-638661817	*Xgpw2331*		
638791957-638796803	C7	TraesCS4A01G365200	Sn1-specific diacylglycerol lipase alpha
